# Supplementary material for: Ligand efficacy shifts a nuclear receptor conformational ensemble between transcriptionally active and repressive states
Source: Nat Commun. 2025 Feb 28;16:2065. doi: 10.1038/s41467-025-57325-4 (PMC11871303; doi:10.1038/s41467-025-57325-4)
Supplement: Supplementary file 1 — Supplementary Information [file 41467_2025_57325_MOESM1_ESM.pdf]

Supplementary Information

Ligand efficacy shifts a nuclear receptor conformational ensemble between transcriptionally active and repressive states

Brian MacTavish [1,#], Di Zhu [2,#], Jinsai Shang [1,3], Qianzhen Shao [4], Yuanjun He [2], Zhongyue J. Yang [4,5,6,7,8,9], Theodore M. Kamenecka [2], and Douglas J. Kojetin [1,2,5,6,7,10,*]

[1] Department of Integrative Structural and Computational Biology, Scripps Research and The Herbert Wertheim UF Scripps Institute for Biomedical Innovation & Technology, Jupiter, Florida 33458, United States

[2] Department of Molecular Medicine, Scripps Research and The Herbert Wertheim UF Scripps Institute for Biomedical Innovation & Technology, Jupiter, Florida 33458, United States

[3] School of Basic Medical Sciences, Guangzhou Laboratory, Guangzhou Medical University, Guangzhou, China.

[4] Department of Chemistry, Vanderbilt University, Nashville, Tennessee 37232, United States

[5] Center for Structural Biology, Vanderbilt University, Nashville, Tennessee 37232, United States

[6] Vanderbilt Institute of Chemical Biology, Vanderbilt University, Nashville, Tennessee 37232, United States

[7] Center for Applied AI in Protein Dynamics, Vanderbilt University, Nashville, Tennessee 37232, United States

[8] Data Science Institute, Vanderbilt University, Nashville, Tennessee 37232, United States

[9] Department of Chemical and Biomolecular Engineering, Vanderbilt University, Nashville, Tennessee 37232, United States

[10] Department of Biochemistry, Vanderbilt University, Nashville, Tennessee 37232, United States.

* Correspondence: douglas.kojetin@vanderbilt.edu

[#] Authors contributed equally

This document contains:

- Supplementary Figs. 1–9
- Supplementary Tables 1–2
- Supplementary Methods
    - Chemical synthesis and characterization

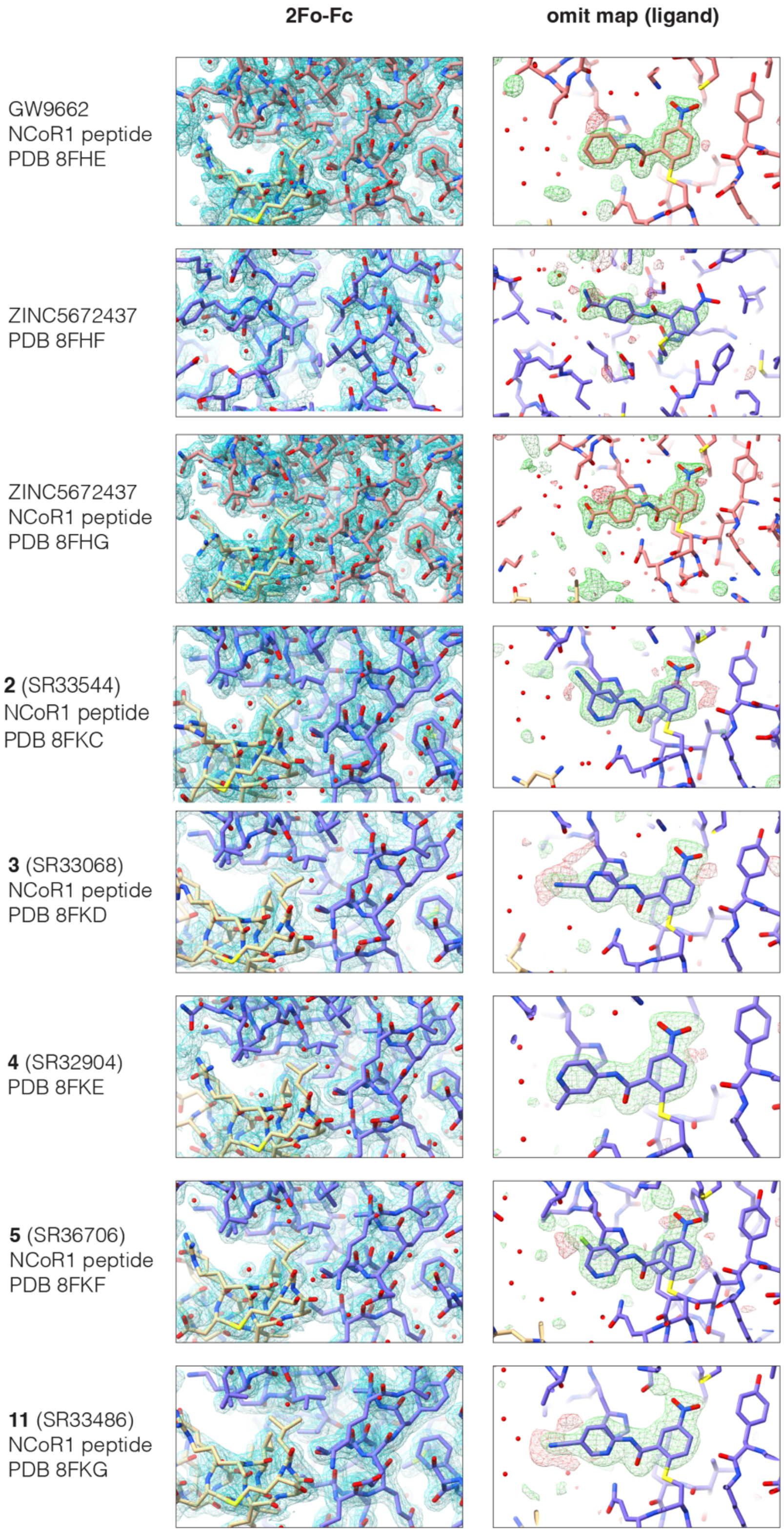

Supplementary Fig. 1. Representative 2Fo-Fc electron density maps contoured to 1σ showing a portion of the crystal structures and omit maps around the ligand contoured at 3σ for all new crystal structures reported in this study.

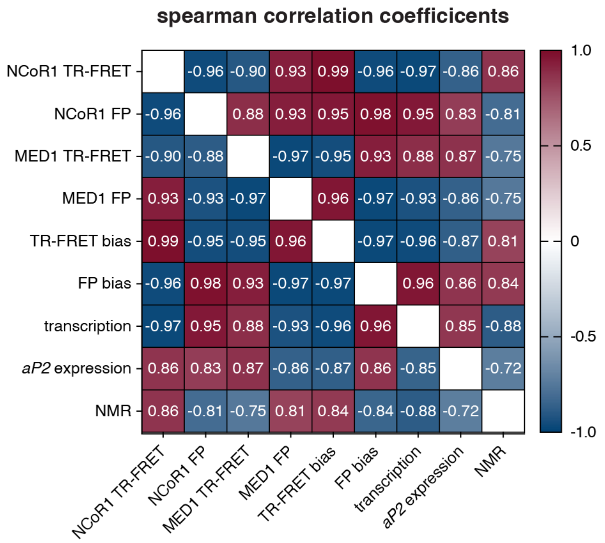

Supplementary Fig. 2. Spearman correlation coefficients calculated from pairwise comparisons of the ligand functional profiling and NMR structural data.

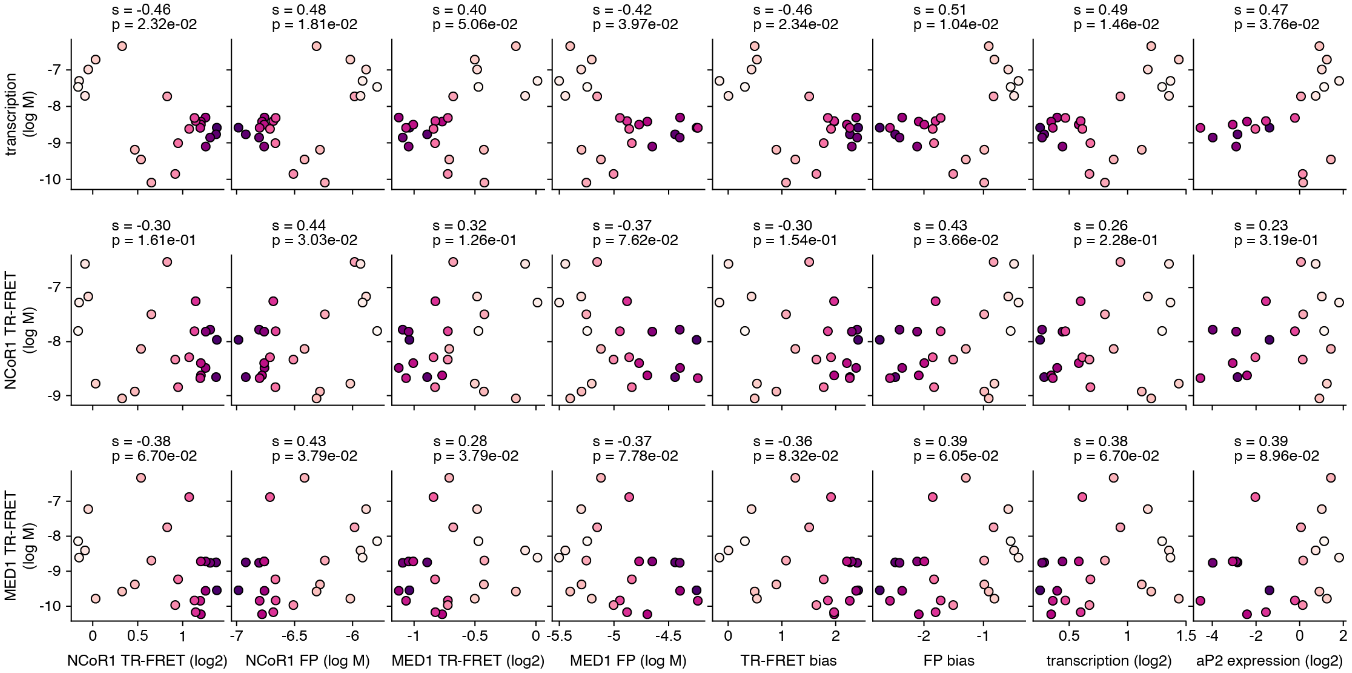

Supplementary Fig. 3. Pairwise correlation analysis reveals a poor correlation between ligand potency vs. efficacy in the compound profiling assay data. Spearman correlation coefficients and associated two-sided p-values are listed. Data are colored from white to purple according to the compound numbers displayed in Fig. 2 (i.e., ranking of functional efficacy in the NCoR1 TR-FRET assay from Fig. 3a).

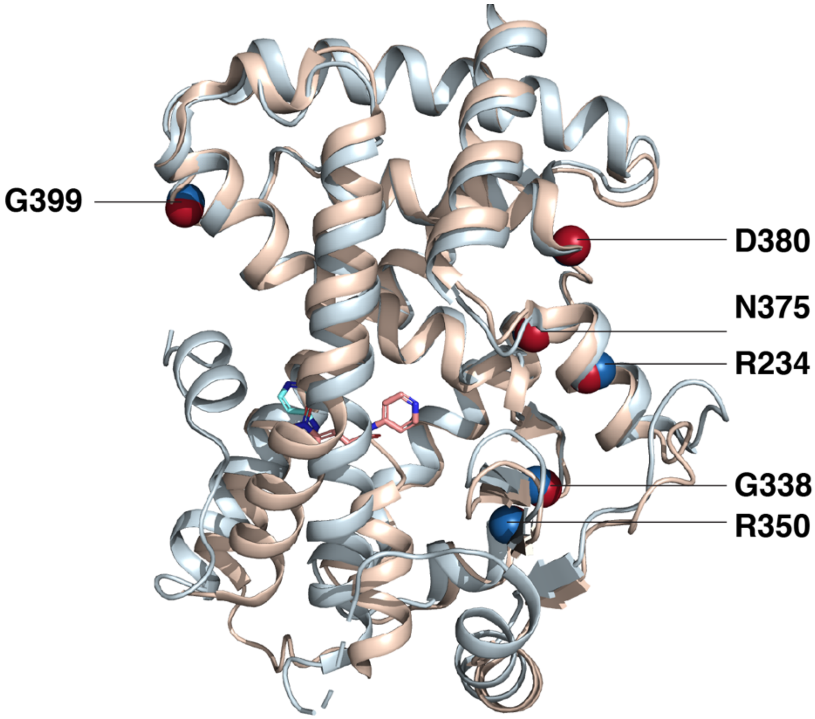

Supplementary Fig. 4. Select PPARγ LBD residues with well-dispersed NMR peaks that display ligand-dependent shifts between active-like and repressive-like states.

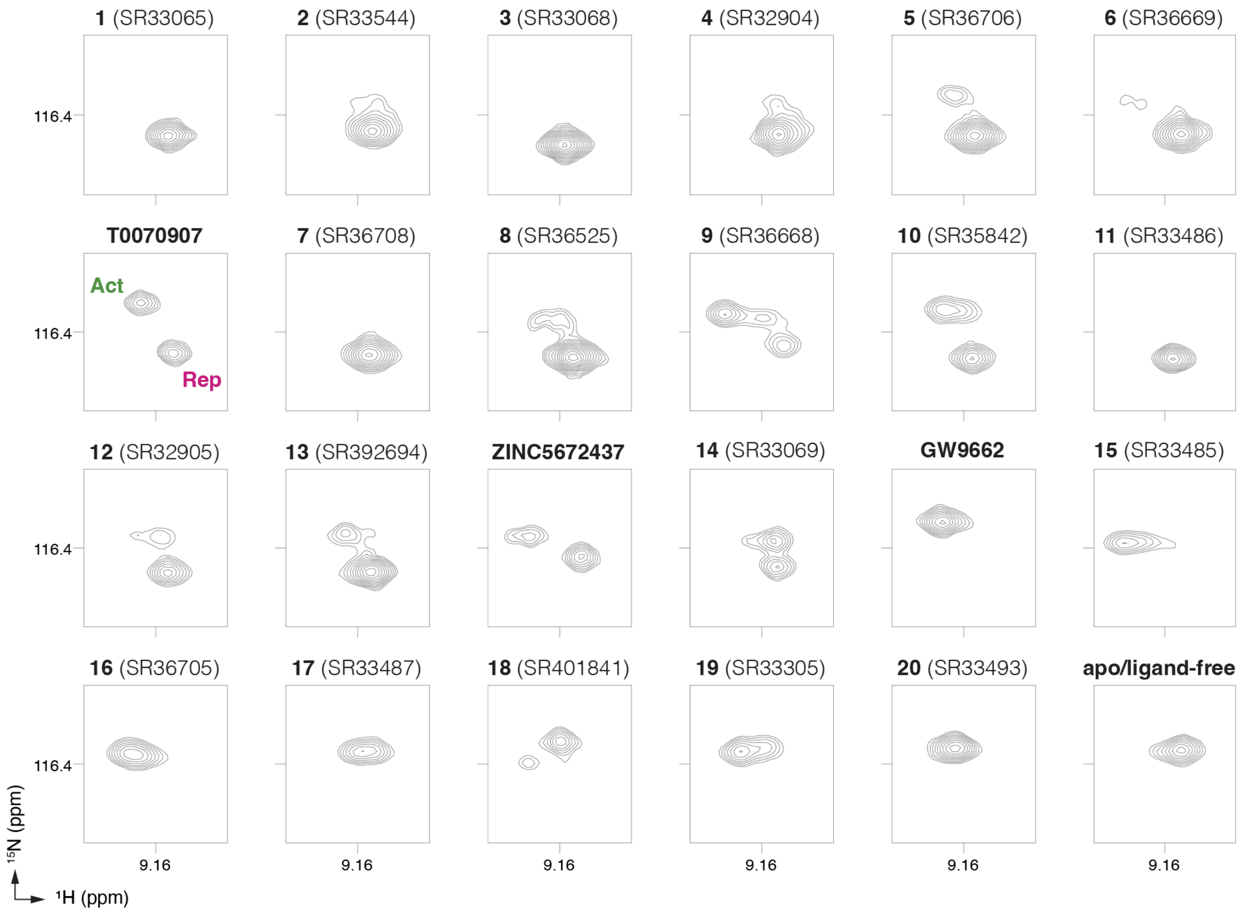

Supplementary Fig. 5. 2D [^{1}H,^{15}N]-TROSY-HSQC NMR focused on Arg234 of ^{15}N-labeled PPARγ LBD bound to compounds in the ligand series. The peak positions of the active-like (Act) and repressive-like (Rep) states populated by T0070907 are noted in green and magenta font color.

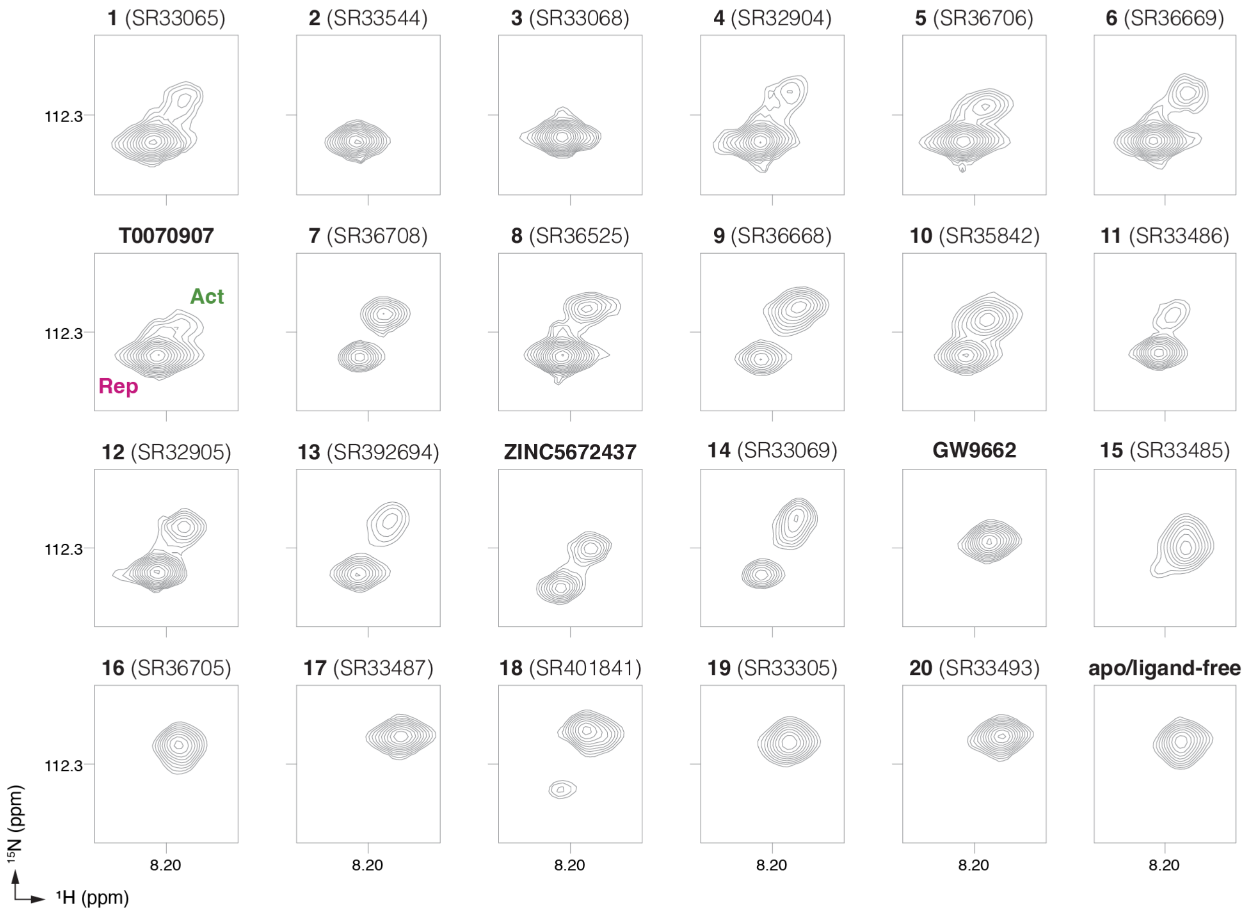

Supplementary Fig. 6. 2D [^{1}H,^{15}N]-TROSY-HSQC NMR focused on Gly338 of ^{15}N-labeled PPARγ LBD bound to compounds in the ligand series. The peak positions of the active-like (Act) and repressive-like (Rep) states populated by T0070907 are noted in green and magenta font color.

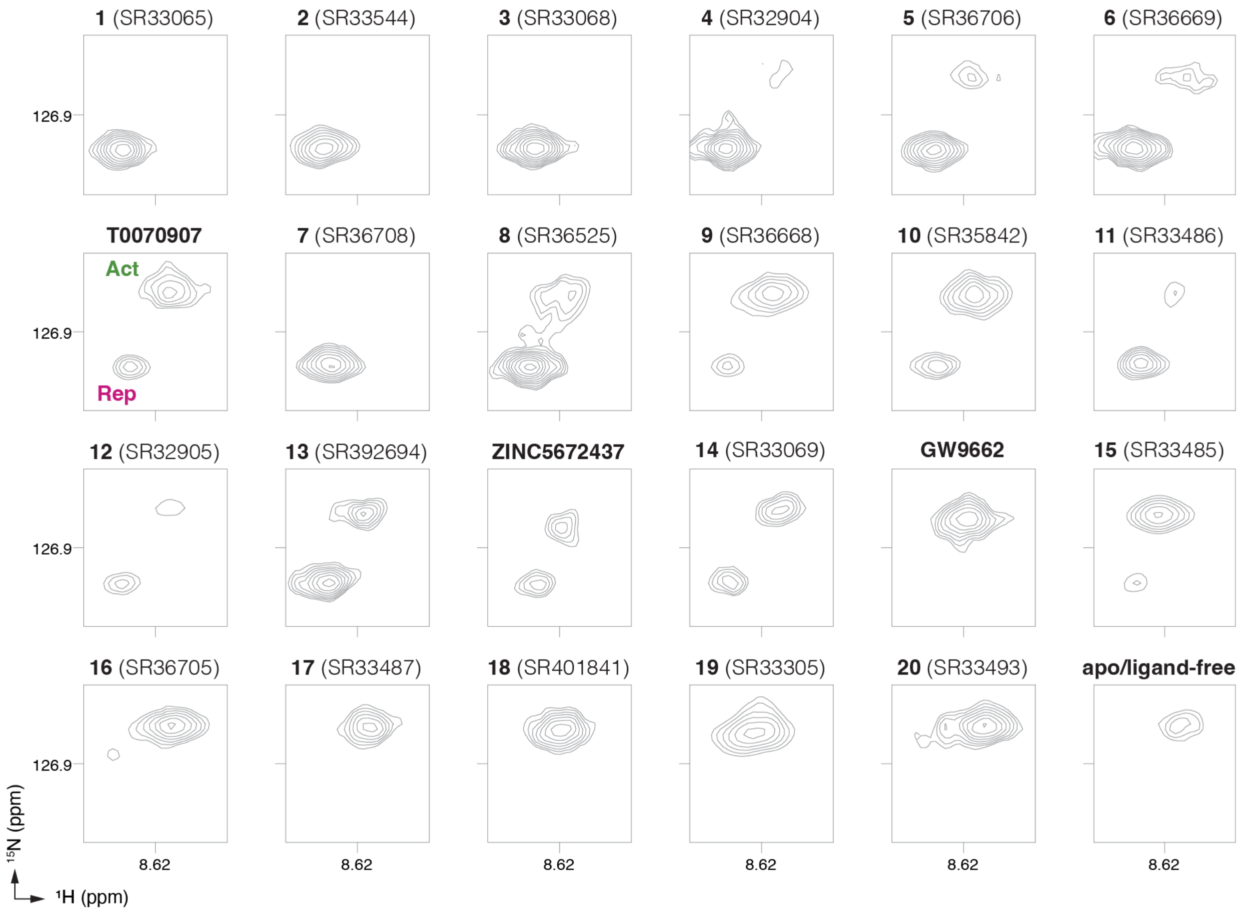

Supplementary Fig. 7. 2D [^{1}H,^{15}N]-TROSY-HSQC NMR focused on Arg350 of ^{15}N-labeled PPARγ LBD bound to compounds in the ligand series. The peak positions of the active-like (Act) and repressive-like (Rep) states populated by T0070907 are noted in green and magenta font color.

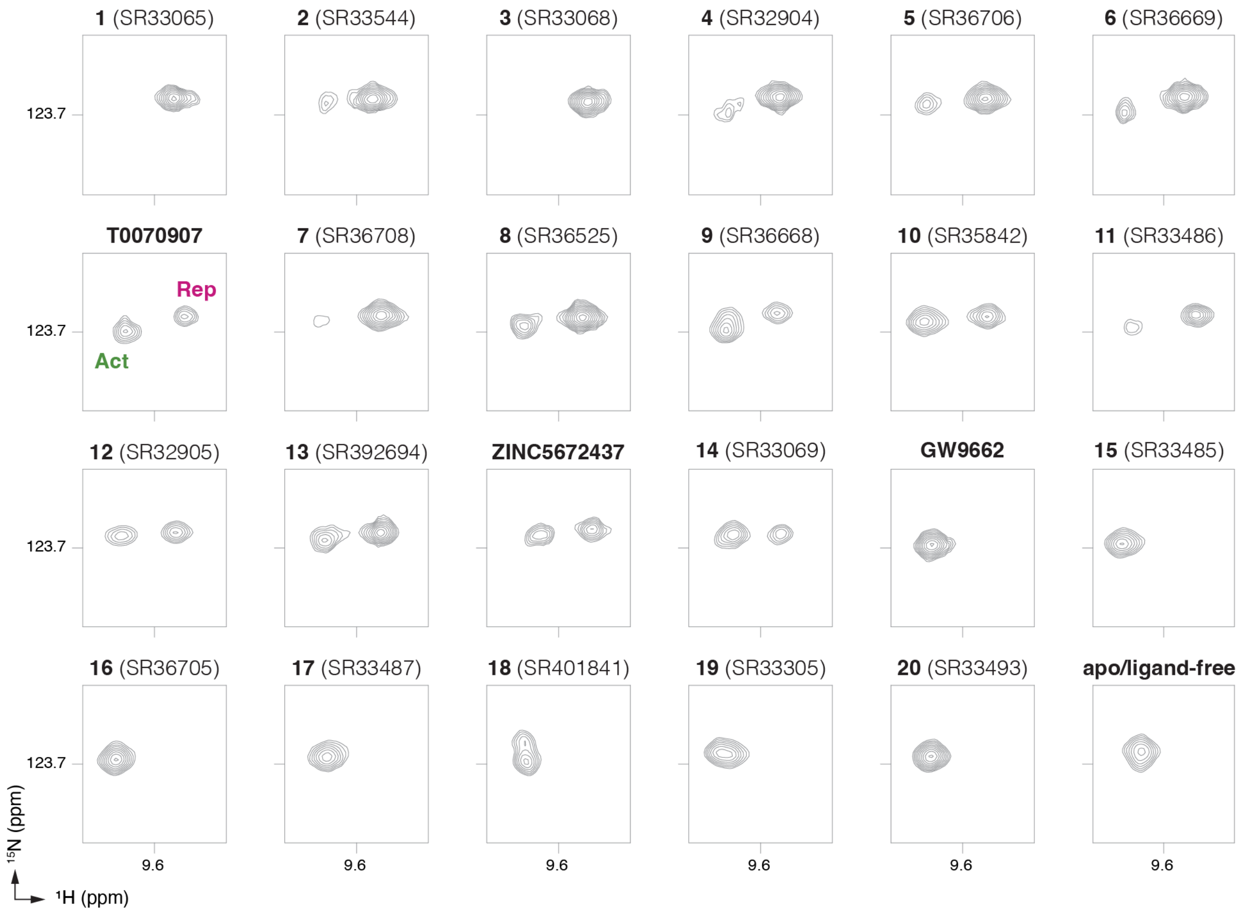

Supplementary Fig. 8. 2D [^{1}H,^{15}N]-TROSY-HSQC NMR focused on Asn375 of ^{15}N-labeled PPARγ LBD bound to compounds in the ligand series. The peak positions of the active-like (Act) and repressive-like (Rep) states populated by T0070907 are noted in green and magenta font color.

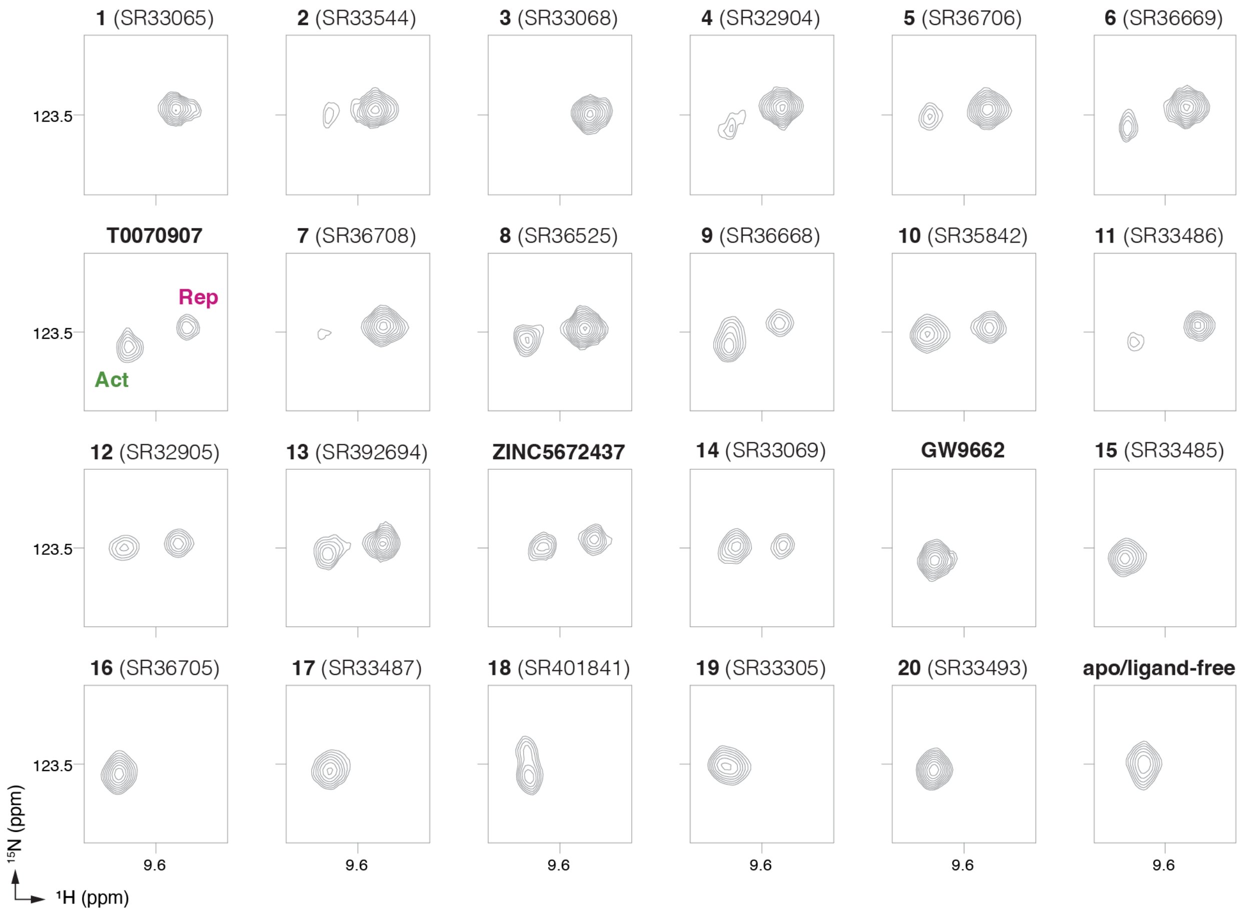

Supplementary Fig. 9. 2D [^{1}H,^{15}N]-TROSY-HSQC NMR focused on Asp380 of ^{15}N-labeled PPARγ LBD bound to compounds in the ligand series. The peak positions of the active-like (Act) and repressive-like (Rep) states populated by T0070907 are noted in green and magenta font color.

Supplementary Table 1. X-ray crystallography data collection and refinement statistics.

	PPARγ LBD + GW9662 and NCoR-D2	PPARγ LBD + ZINC5672437 and NCoR-D2	PPARγ LBD + ZINC5672437	PPARγ LBD + NCoR-D2 and SR33544	PPARγ LBD + NCoR-D2 and SR33068	PPARγ LBD + NCoR-D2 and SR32904	PPARγ LBD + NCoR-D2 and SR36706	PPARγ LBD + NCoR-D2 and SR33486
**Data collection**								
Space group	C 4_1 2_1 2_1	C 4_1 2_1 2_1	C 1 2 1	P 4_1 2_1 2	P 4_1 2_1 2	P 4_1 2_1 2	P 4_1 2_1 2	P 4_1 2_1 2
Cell dimensions								
a, *b*, *c* (Å)	61.72, 61.72, 163.92	61.05, 61.05, 159.29	91.63, 60.70, 117.08	62.054, 62.054, 160.719	62.125, 62.125, 162.209	61.888, 61.888, 164.288	61.765, 61.765, 161.08	61.968, 61.968, 163.608
α, β, γ (°)	90, 90, 90	90, 90, 90	90, 102.50, 90	90, 90, 90	90, 90, 90	90, 90, 90	90, 90, 90	90, 90, 90
Resolution (Å)	34.14-1.8 (1.864-1.8)	37.95-1.8 (1.864-1.8)	38.89-2.10 (2.175-2.10)	43.88-1.42 (1.468-1.42)	42.4-2.22 (2.302-2.22)	41.07-2.02 (2.088-2.02)	42.15-1.82 (1.89-1.82)	30.98-2.12 (2.195-2.12)
R_{merge}	0.013 (0.156)	0.020 (0.162)	0.016 (0.246)	0.03887 (1.065)	0.1217 (1.479)	0.07696 (1.073)	0.05594 (0.4607)	0.1486 (1.084)
$I / \sigma I$	28.00 (4.58)	19.45 (3.89)	16.87 (2.07)	44.98 (2.64)	13.50 (1.91)	23.01 (2.28)	39.82 (6.35)	29.48 (4.01)
Completeness (%)	99.93 (99.73)	99.94 (99.96)	97.43 (97.83)	99.87 (98.90)	98.61 (86.71)	99.66 (97.86)	99.83 (99.64)	99.90 (100.00)
Redundancy	2.0 (2.0)	2.0 (2.0)	2.0 (2.0)	25.5 (20.8)	12.4 (10.5)	12.6 (12.0)	25.1 (26.2)	25.2 (26.4)
**Refinement**								
Resolution (Å)	1.80	1.80	2.10	1.42	2.222	2.02	1.825	2.12
No. unique reflections	30317 (2980)	28829 (2799)	35970 (3565)	60517 (5857)	16390 (1571)	21902 (2109)	28621 (2792)	18917 (1851)
R_{work} / R_{free}	19.8/22.6	20.2/23.4	24.4/28.8	18.53/19.75	21.66/25.14	19.43/24.91	18.08/21.64	18.52/21.83
No. atoms								
Protein	2191	2230	4034	2190	2254	2258	2217	2214
Ligand/ion	18	21	64	24	32	27	41	70
Water	270	219	258	269	98	241	219	123
B-factors								
Protein	28.13	26.11	29.94	30.09	50.82	41.18	33.62	44.94
Ligand/ion	25.67	22.64	36.65	29.45	46.72	40.05	37.15	51.17
Water	36.12	32.96	30.08	37.90	44.89	42.76	38.49	45.65
R.m.s. deviations								
Bond lengths (Å)	0.009	0.008	0.011	0.011	0.021	0.002	0.010	0.020
Bond angles (°)	1.03	0.93	1.13	1.18	1.67	0.46	1.04	1.44
Ramachandran favored (%)	97.38	98.89	97.15	98.50	98.55	98.55	99.26	99.26
Ramachandran outliers (%)	0.37	0.00	0.00	0.00	0.00	0.00	0.00	0.00
PDB accession code	8FHE	8FHG	8FHF	8FKC	8FKD	8FKE	8FKF	8FKG

*Values in parentheses are for highest-resolution shell.

Supplementary Table 2. Density functional theory (DFT) quantum mechanical (QM) interaction free energies (ΔG_{bind}) between compounds and PPARγ LBD residues comprising the pi-stacking aromatic triad residues (His323, His449, Tyr473) and others nearby residues (Cys285, Gln286, Tyr327, Met364, Lys367).

Compound number	ΔG_{BIND} (crystallized conformation)	ΔG_{BIND} (flipped r_1 ring conformation)
T0070907	-15.5	n.d.
2 (SR33544)	-19.3	n.d.
3 (SR33068)	-17.4	-16.9
4 (SR32904)	-17.0	n.d.
5 (SR36706)	-17.2	n.d.
11 (SR33486)	-21.3	-27.5

Supplementary Methods

Chemical synthesis and characterization

General information:. NMR spectra were recorded in DMSO-*d6,* MeOD or deuterated chloroform ($CDCl_3$) on a Bruker AVANCE DPX-400 (400 MHz) and an AVANCE III 600 (600 MHz) spectrometer. Chemical shifts (δ) were calibrated relative to solvent peaks and are reported in parts per million (ppm), whereas the coupling constants (*J*) are reported in Hertz (Hz). Abbreviations for the peak multiplicities are as follows: s (singlet), brs (broad singlet), d (doublet), t (triplet), q (quartet), dd (doublet of doublets), dt (doublet of triplets) and m (multiplet). Mass spectrometry (MS) was performed using electrospray ionization on an Agilent 6230 TOF LC/MS mass spectrometer in the positive ion mode. The purity (≥ 95%) of all final synthesized compounds was determined by NMR. Compounds are listed by the chemical name (compound number, internal referencing code).

Chemical Synthesis of Analogs:

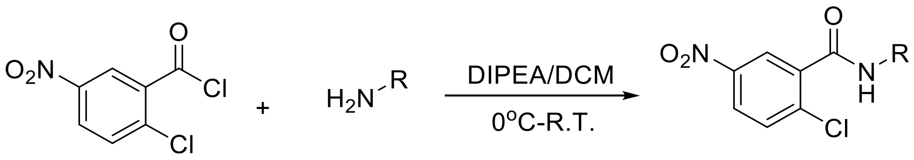

To a solution of aniline (1.1 mmol, 1.1 equiv.) and diisopropylethylamine (0.2 mL, 1.1 mmol, 1.1 equiv.) in dichloromethane (DCM, 1.5 mL) was added a solution of 2-chloro-5-nitrobenzoyl chloride (220 mg, 1mmol, 1.0 equiv.) in DCM (0.5L) dropwise in an ice-water bath. After 15 min, the reaction was allowed to warm to room temperature for 3-8 hours. The reaction mixture was diluted with ethyl acetate (EtOAc, 10 mL), washed with water (10 mL), saturated $NaHCO_3$ (10 mL) and brine (10 mL). The organic layer was dried over $MgSO_4$, filtered, and concentrated under reduced pressure. The crude material was purified by silica gel chromatography to yield the title compound as a white solid.

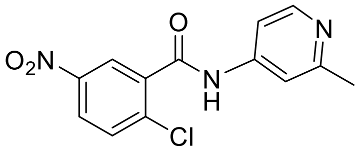

SR32904

2-chloro-N-(2-methylpyridin-4-yl)-5-nitrobenzamide

^{1}HNMR ($CDCl_3$, 600 MHz), δ(ppm): 8.61 (d, *J*=2.8 Hz, 1H), 8.47 (d, *J*=6.0 Hz, 1H), 8.30 (dd, J_1=2.8Hz, J_2=8.0 Hz, 1H), 8.02 (br, 1H), 7.70 (d, *J*=9.0 Hz, 1H), 7.51 (s, 1H), 7.36 (d, *J*=4.2 Hz, 1H), 2.58 (s, 3H). **^{13}CNMR ($CDCl_3$, 150 MHz)**, δ (ppm): 162.56, 160.18, 150.33, 146.75, 144.23, 137.41, 135.72, 131.84, 126.50, 125.48, 113.20, 111.26, 24.65. **LCMS (ESI)**: Expected mass for $C_{13}H_{11}ClN_3O_3$ $(M + H)^+$: 292.05 Da, found 292.05 Da.

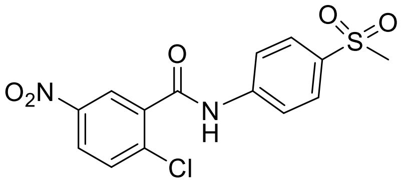

SR32905

2-chloro-N-(4-(methylsulfonyl)phenyl)-5-nitrobenzamide

^{1}HNMR (DMSO-d6, 400 MHz), δ (ppm): 11.16 (s, 1H), 8.55 (d, *J*=2.9 Hz, 1H), 8.37 (dd, J_1=2.9Hz, J_2=8.8 Hz, 1H), 7.95 (s, 4H), 7.92 (d, *J*=8.8 Hz, 1H), 3.20 (s, 3H); **^{13}CNMR ($CDCl_3$, 100 MHz)**, δ (ppm): 163.90, 146.63, 143.33, 137.54, 137.49, 136.14, 131.90, 128.80, 126.52, 124.43, 120.20, 44.24. **LCMS (ESI)**: Expected mass for $C_{14}H_{12}ClN_2O_5S$ $(M + H)^+$: 355.02 Da, found 355.31 Da.

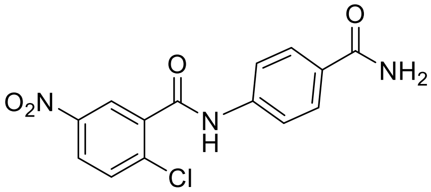

SR33064

N-(4-carbamoylphenyl)-2-chloro-5-nitrobenzamide

^{1}HNMR (DMSO-d6, 400 MHz), δ (ppm): 10.93 (s, 1H), 8.51 (d, *J*=2.7 Hz, 1H), 8.36 (dd, J_1=2.7Hz, J_2=8.9 Hz, 1H), 7.92 (br, 2 H), 7.91 (d, *J*=8.7 Hz, 2H), 7.77 (d, *J*=8.7Hz, 2H), 7.31 (br, 1H). **^{13}CNMR ($CDCl_3$, 100 MHz)**, δ (ppm): 168.30, 163.74, 146.58, 141.47, 137.62, 137.50, 131.87, 129.98, 128.97, 126.37, 124.23, 119.58. **LCMS (ESI)**: Expected mass for $C_{14}H_{11}ClN_3O_4$ $(M + H)^+$: 320.04 Da, found 320.04 Da.

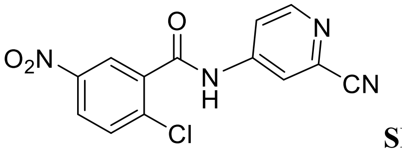

SR33065

2-chloro-N-(2-cyanopyridin-4-yl)-5-nitrobenzamide

^{1}HNMR (DMSO-d6, 400 MHz), δ (ppm): 11.50 (s, 1H), 8.70 (d, *J*=5.8 Hz, 1H), 8.61 (d, *J*=2.5Hz, 1H), 8.40 (dd, J_1=2.8Hz, J_2=8.4 Hz, 1H), 8.21 (d, *J*=1.8 Hz, 1H), 7.95 (d, *J*=8.6Hz, 1H), 7.91 (dd, J_1=2.2Hz, J_2=5.4 Hz, 1H); **^{13}CNMR ($CDCl_3$, 100 MHz)**, δ (ppm): 164.61, 153.03, 146.68, 146.61, 137.48, 136.67, 133.88, 132.04, 126.93, 124.61, 119.05, 117.86, 117.42. **LCMS (ESI)**: Expected mass for $C_{13}H_8ClN_4O_3$ $(M + H)^+$: 303.03 Da, found 303.03 Da.

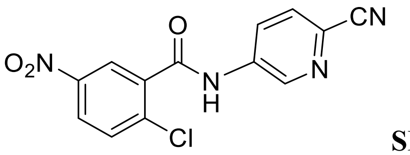

SR33068

2-chloro-N-(6-cyanopyridin-3-yl)-5-nitrobenzamide

^{1}HNMR (DMSO-d6, 400 MHz), δ (ppm): 11.44 (s, 1H), 8.96 (d, J=2.2Hz, 1H), 8.61 (d, J=2.9Hz, 1H), 8.40 (t, J=2.6Hz, 1H), 8.38 (t, J=2.6Hz, 1H), 8.08 (d, J=8.7 Hz, 1H), 7.94 (d, J=8.7 Hz, 1H). **^{13}CNMR ($CDCl_3$, 100 MHz)**, δ (ppm): 164.21, 146.63, 142.87, 138.91, 137.54, 136.99, 132.00, 130.24, 127.42, 127.22, 126.80, 124.69, 118.05. **LCMS (ESI)**: Expected mass for $C_{13}H_8ClN_4O_3$ $(M + H)^+$: 303.03 Da, found 303.18 Da.

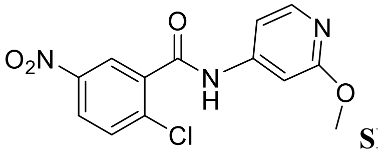

SR33069

2-chloro-N-(2-methoxypyridin-4-yl)-5-nitrobenzamide

^{1}HNMR (DMSO-d6, 400 MHz), δ (ppm): 11.02 (s, 1H), 8.53 (d, J=2.5 Hz, 1H), 8.37 (dd, J_1=2.8Hz, J_2=8.8 Hz, 1H), 8.11 (dd, J_1=1.9 Hz, J_2=5.5 Hz, 1H), 7.92 (d, J=8.8Hz, 1H), 7.20-7.21 (m, 2H), 3.86 (s, 3H). **^{13}CNMR ($CDCl_3$, 100 MHz)**, δ (ppm):165.08, 164.33, 148.58, 147.97, 146.60, 137.80, 137.46, 131.87, 126.36, 124.44, 109.23, 99.76,53.69. **LCMS (ESI)**: Expected mass for $C_{13}H_{11}ClN_3O_4$ $(M + H)^+$: 308.04 Da, found 308.04 Da.

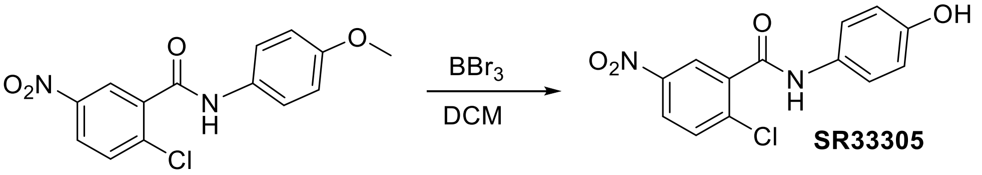

SR33305

2-chloro-N-(4-hydroxyphenyl)-5-nitrobenzamide

To a stirred solution of 2-chloro-N-(4-methoxyphenyl)-5-nitrobenzamide (0.12 g, 0.4 mmol) in dry DCM (5 mL), cooled to -78°C, was added slowly BBr_3 (0.8 mL of a 1M solution, 0.8 mmol) and the reaction was allowed to warm slowly to room temperature with stirring overnight before being quenched with water. The organic layer was separated and dried over $MgSO_4$, filtered, and concentrated under reduced pressure. The crude material was purified by silica gel chromatography to yield the title compound as a white solid. **^{1}HNMR (DMSO-d6, 400 MHz)**, δ (ppm): 11.02 (s, 1H), 8.54 (d, J=2.7 Hz, 1H), 8.37 (dd, J_1=2.6Hz, J_2=8.8 Hz, 1H), 7.92 (d, J=8.8Hz, 1H), 7.53 (d, J=7.2 Hz, 1H), 7.00 (s, 1H), 6.60 (d, J=6.9Hz, 1H). **^{13}C NMR ($CDCl_3$, 100 MHz)**, δ (ppm): 162.65, 154.54, 146.59, 138.45, 137.60, 131.77, 130.58, 125.98, 124.25, 122.01, 115.66. **LCMS (ESI)**: Expected mass for $C_{13}H_{10}ClN_2O_4$ $(M + H)^+$: 293.03 Da, found 293.07 Da.

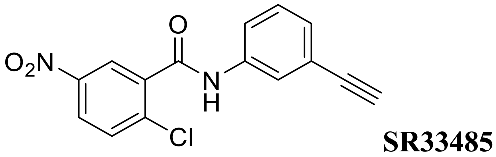

SR33485

2-chloro-N-(3-ethynylphenyl)-5-nitrobenzamide

^{1}HNMR (DMSO, 400 MHz), δ (ppm): 8.62 (d, J=2.7 Hz, 1H), 8.29 (dd, J_1=8.9Hz, J_2=2.8Hz, 1H), 7.85 (br, 1H), 7.76 (s, 1H), 7.70-7.66 (m, 2H), 7.39-7.33 (m, 2H), 3.11 (s, 1H). **^{13}CNMR (DMSO, 100 MHz)**, δ (ppm):163.48, 146.64, 139.13, 137.89, 137.53, 131.86, 129.90, 127.95, 126.32, 124.38, 123.09, 122.63, 120.89, 83.63, 81.33. **LCMS (ESI)**: Expected mass for $C_{15}H_{10}ClN_2O_3$ $(M + H)^+$: 301.04 Da, found 301.28 Da.

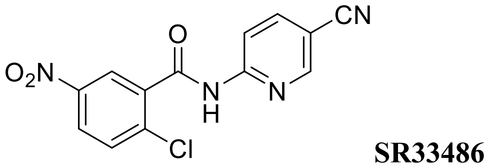

SR33486

2-chloro-N-(5-cyanopyridin-2-yl)-5-nitrobenzamide

^{1}HNMR (DMSO-d6, 400 MHz), δ (ppm): 11.78 (s, 1H), 8.85 (t, J=1.45 Hz, 1H), 8.55 (d, J=2.90Hz, 1H), 8.33-8.36 (m, 3H), 7.87 (d, J=9.3Hz, 1H). **^{13}C NMR (DMSO, 150 MHz)**, δ (ppm): 164.71, 154.51, 152.67, 146.48, 142.81, 137.48, 137.14, 131.71, 126.56, 124.75, 117.60, 114.23, 104.89. **LCMS (ESI)**: Expected mass for $C_{13}H_8ClN_4O_3$ $(M + H)^+$: 303.03 Da, found 303.25 Da.

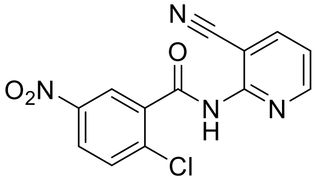

SR33487

2-chloro-N-(3-cyanopyridin-2-yl)-5-nitrobenzamide

^{1}HNMR (DMSO-d6, 400 MHz), δ (ppm): 11.71 (s, 1H), 8.77 (dd, J_1=1.7Hz, J_2=4.8 Hz, 1H), 8.45 (dd, J_1=1.9Hz, J_2=7.7 Hz, 1H), 8.38 (m, 2H), 7.93 (d, J=9.4Hz, 1H), 7.56 (dd, J_1=4.8Hz, J_2=7.8Hz, 1H). **^{13}C NMR (DMSO, 150 MHz)**, δ (ppm): 164.03, 153.29, 151.58, 146.54, 143.46, 137.78, 136.45, 132.18, 126.86, 124.47, 122.37, 115.96, 105.55. **LCMS (ESI)**: Expected mass for $C_{13}H_8ClN_4O_3$ $(M + H)^+$: 303.03 Da, found 303.25 Da.

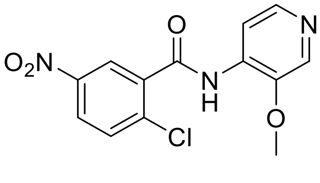

SR33493

2-chloro-N-(3-methoxypyridin-4-yl)-5-nitrobenzamide

^{1}H NMR (DMSO-d6, 400 MHz), δ (ppm): 10.42 (s, 1H), 8.44 (d, J=2.7 Hz, 1H), 8.40 (br, 1H), 8.33 (dd, J_1=2.7Hz, J_2=8.8 Hz, 1H), 8.21 (m, 2H), 7.86 (d, J=8.9 Hz, 1H), 3.93 (s, 3H). **^{13}CNMR (DMSO, 100 MHz)**, δ (ppm): 164.49, 146.47, 143.40, 137.71, 137.52, 134.62, 134.04, 131.55, 126.19, 124.59, 57.00. **LCMS (ESI)**: Expected mass for $C_{13}H_{11}ClN_3O_4$ $(M + H)^+$: 308.04 Da, found 308.04 Da.

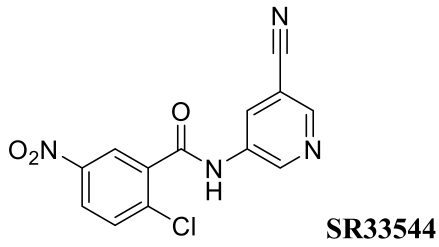

SR33544

2-chloro-N-(5-cyanopyridin-3-yl)-5-nitrobenzamide

^{1}HNMR (DMSO-d6, 400 MHz), δ (ppm): 11.33 (s, 1H), 9.03 (d, J=2.5 Hz, 1H), 8.83 (d, J=2.5Hz, 1H), 8.59 (t, J=2.1 Hz, 2H), 8.39 (dd, J_1=2.9Hz, J_2=8.9 Hz, 1H), 7.94 (d, J=8.9 Hz, 1H). **^{13}CNMR (MeOH-d4, 100 MHz)**, δ (ppm): 199.47, 164.63, 147.18, 146.62, 144.27, 139.29, 137.53, 136.56, 131.95, 131.30, 129.64, 126.02, 125.87, 125.47, 123.80, 115.78, 37.48. **LCMS (ESI)**: Expected mass for $C_{13}H_8ClN_4O_3$ $(M + H)^+$: 303.03 Da, found 303.25 Da.

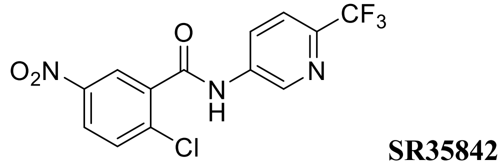

SR35842

2-chloro-5-nitro-N-(6-(trifluoromethyl)pyridin-3-yl)benzamide

^{1}HNMR (DMSO-d6, 400 MHz), δ (ppm): 11.35 (s, 1H), 8.97 (d, J=2.82Hz, 1H), 8.44 (dd, J_1=2.07 Hz, J_2=8.46Hz), 8.39 (dd, J_1=2.07Hz, J_2=8.46Hz, 1H), 7.97 (d, J=8.96Hz, 1H), 7.94 (d, J=8.96Hz, 1H). **^{13}C NMR ($CDCl_3$, 100 MHz)**, δ (ppm): 164.21, 146.63, 141.74, 138.57, 137.55, 137.14, 131.98, 127.97, 126.72, 124.64, 121.92. **LCMS (ESI)**: Expected mass for $C_{13}H_8ClF_3N_3O_3$ $(M + H)^+$: 346.02 Da, found 346.02 Da.

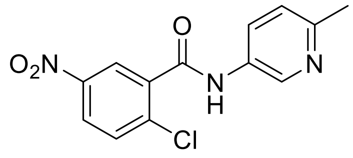

SR36525

2-chloro-N-(6-methylpyridin-3-yl)-5-nitrobenzamide

^{1}HNMR (DMSO-d6, 400 MHz), δ (ppm): 10.85 (s, 1H), 8.71 (d, J=2.4 Hz, 1H), 8.51 (d, J=2.7 Hz, 1H), 8.35 (dd, J_1=8.8 Hz, J_2=2.7 Hz, 1H), 8.02 (dd, J_1=8.4 Hz, J_2=2.7 Hz, 1H), 7.90 (d, J=8.9 Hz, 1H), 7.29 (d, J= 8.3Hz, 1H), 2.46 (s, 3H). **^{13}CNMR ($CDCl_3$, 100 MHz)**, δ (ppm): 163.52, 153.93, 146.61, 141.05, 137.73, 137.56, 133.20, 131.88, 127.97, 126.36, 124.44, 123.43, 23.90. **LCMS (ESI)**: Expected mass for $C_{13}H_{11}ClN_3O_3$ $(M + H)^+$: 292.05 Da, found 292.05 Da.

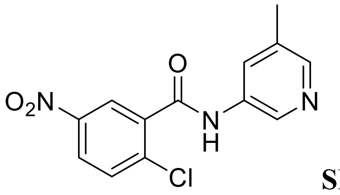

SR36668

2-chloro-N-(5-methylpyridin-3-yl)-5-nitrobenzamide

^{1}HNMR (MeOD-d4, 400 MHz), δ (ppm): 9.33 (s, 1H), 8.56 (d, *J*=2.78Hz, 1H), 8.54 (br, 1H), 8.42 (d, *J*=2.78Hz, 1H), 8.40 (d, *J*=2.62Hz, 1H), 7.86 (d, *J*=8.83Hz, 1H), 2.60 (s, 3H). **^{13}CNMR (MeOD-d4, 100 MHz)**, δ (ppm): 164.55, 146.61, 137.56, 136.07, 135.14, 131.41, 130.61, 126.12, 123.87, 17.10. **LCMS (ESI)**: Expected mass for $C_{13}H_{11}ClN_3O_3$ $(M + H)^+$: 292.04 Da, found 292.04 Da.

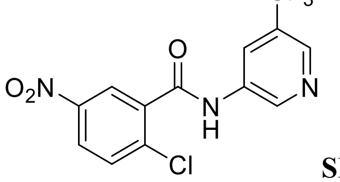

SR36669

2-chloro-5-nitro-N-(5-(trifluoromethyl)pyridin-3-yl)benzamide

[1] HNMR (MeOD-d4, 400 MHz), δ (ppm): 9.03 (d, *J*=2.03Hz, 1H), 8.70 (s, 1H), 8.67 (t, *J*=1.85Hz, 1H), 8.56 (d, *J*=2.49Hz, 1H), 8.39 (dd, J_1=2.49Hz, J_2=8.78Hz, 1H), 7.85 (d, *J*=8.78Hz, 1H). **^{13}C NMR (MeOD-d4, 100 MHz)**, δ (ppm): 164.67, 146.63, 144.18, 141.11, 141.07, 137.55, 136.64, 135.68, 131.28, 126.88, 125.83, 123.79, 122.00. **LCMS (ESI)**: Expected mass for $C_{13}H_8ClF_3N_3O_3$ $(M +H)^+$: 346.02 Da, found 346.02 Da.

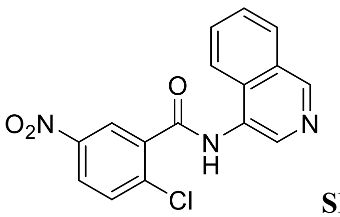

SR36705

2-chloro-N-(isoquinolin-4-yl)-5-nitrobenzamide

^{1}HNMR (DMSO-d6, 400 MHz), δ (ppm): 11.08 (s, 1H), 8.91 (d, *J*=4.93 Hz, 1H), 8.64 (d, *J*=2.78 Hz, 1H), 8.39 (dd, J_1=2.78 Hz, J_2=8.64 Hz, 1H), 8.34 (dd, J_1=0.85 Hz, J_2= 8.78 Hz, 1H), 8.15 (d, *J*=4.58 Hz, 1H), 8.06 (dd, J_1= 0.62Hz, J_2= 8.40Hz, 1H), 7.94 (d, *J*= 8.77Hz, 1H), 7.81 (dt, J_1= 1.24Hz, J_2= 8.31Hz, 1H), 7.65 (dt, J_1= 1.24Hz, J_2= 8.31Hz, 1H). **^{13}CNMR ($CDCl_3$, 100 MHz)**, δ (ppm): 164.70, 151.30, 149.19, 146.68, 141.32, 137.88, 137.55, 131.72, 130.18, 129.87, 126.72, 126.40, 124.71, 123.13, 121.77, 113.96. **LCMS (ESI)**: Expected mass for $C_{16}H_{11}ClN_3O_3$ $(M + H)^+$: 328.05 Da, found 328.25 Da.

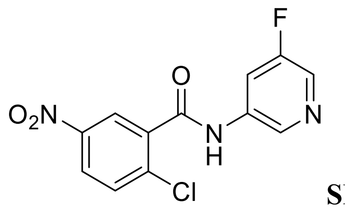

SR36706

2-chloro-N-(5-fluoropyridin-3-yl)-5-nitrobenzamide

^{1}HNMR (DMSO-d6, 400 MHz), δ (ppm): 11.22 (br, 1H), 8.69 (br, 1H), 8.85 (d, *J*=2.70 Hz, 1H), 8.41 (br, 1H), 8.37 (dd, J_1=2.70 Hz, J_2=8.70 Hz, 1H), 8.14 (d, *J*=10.90 Hz, 1H), 7.92 (d, *J*= 8.66Hz, 1H). **^{13}C NMR ($CDCl_3$, 100 MHz)**, δ (ppm): 164.05, 146.63, 137.86, 137.54, 137.22, 131.97, 126.66, 124.57, 114.27, 114.05. **LCMS (ESI)**: Expected mass for $C_{12}H_8ClFN_3O_3$ $(M + H)^+$: 296.02 Da, found 296.02 Da.

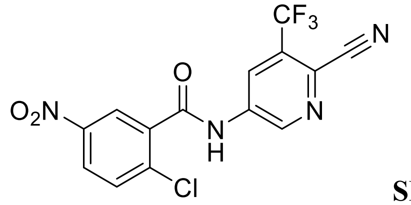

SR36708

2-chloro-N-(6-cyano-5-(trifluoromethyl)pyridin-3-yl)-5-nitrobenzamide

^{1}HNMR (DMSO-d6, 400 MHz), δ (ppm): 11.78 (s, 1H), 9.14 (d, *J*=1.84 Hz, 1H), 8.76 (d, *J*=2.12 Hz, 1H), 8.64 (d, *J*=2.74 Hz, 1H), 8.41 (dd, J_1=2.87 Hz, J_2= 8.78 Hz, 1H), 7.95 (d, *J*=8.86 Hz, 1H). **^{13}CNMR ($CDCl_3$, 100 MHz)**, δ (ppm): 164.60, 146.61, 145.15, 138.91, 137.60, 136.38, 132.15, 129.80, 127.14, 124.84, 124.21, 123.79, 115.21. **LCMS (ESI)**: Expected mass for $C_{14}H_7ClF_3N_4O_3$ $(M + H)^+$: 371.02 Da, found 371.25 Da.

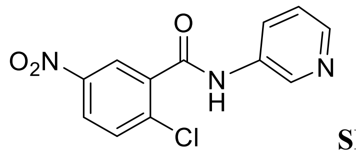

SR1000392694

2-chloro-5-nitro-N-(pyridin-3-yl)benzamide

^{1}HNMR ($CDCl_3$, 400 MHz), δ (ppm): 8.67 (d, *J*=2.7 Hz, 1H), 8.63 (d, *J*=2.7 Hz, 1H), 8.44 (d, *J*=4.5 Hz, 1H), 8.29 (dd, J_1=3.2Hz, J_2=9.1Hz, 2H), 8.16 (br, 1H), 7.69 (d, *J*=8.6 Hz, 1H), 7.37 (d, *J*=3.2Hz, 2H). **^{13}CNMR ($CDCl_3$, 100 MHz)**, δ (ppm): 163.72, 146.63, 145.65, 141.79, 137.65, 137.55, 135.67, 131.90, 127.33, 126.44, 124.48, 124.29. **LCMS (ESI)**: Expected mass for $C_{12}H_9ClN_3O_3$ $(M + H)^+$: 278.03 Da, found 278.03 Da.

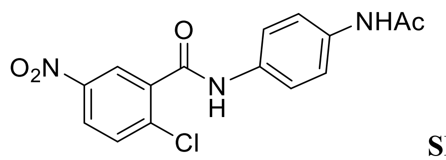

SR1000401841

N-(4-acetamidophenyl)-2-chloro-5-nitrobenzamide

^{1}HNMR (DMSO-d6, 400 MHz), δ (ppm): 10.65 (br, 1H), 9.96 (br, 1H), 8.42 (d, *J*=2.55 Hz, 1H), 8.33 (dd, J_1=2.55Hz, J_2=8.92 Hz, 1H), 7.87 (d, *J*=8.92Hz, 1H), 7.55-7.62 (m, 4H), 2.03 (s, 3H). **^{13}CNMR (DMSO-d6, 100 MHz)**, δ (ppm): 168.65, 162.96, 146.60, 138.21, 137.57, 136.14, 134.11, 131.80, 126.11, 124.30, 120.69, 119.86, 24.35. **LCMS (ESI)**: Expected mass for $C_{15}H_{13}ClN_3O_4$ $(M + H)^+$: 334.06 Da, found 334.23 Da.

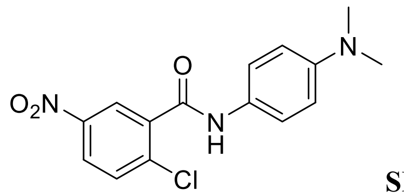

SR33066

2-chloro-N-(4-(dimethylamino)phenyl)-5-nitrobenzamide

^{1}H NMR (DMSO-d6, 600 MHz), δ (ppm): 10.42 (s, 1H), 8.44 (s, 1H), 8.43 (d, J=4.0 Hz, 1H), 8.35 (dd, J=8.0, 4.0 Hz, 1H), 7.91 (d, J=8.0 Hz, 1H), 7.56 (d, J= 4.0 Hz, 2H), 6.77(d, J=4.0 Hz, 2H), 2.9 (s, 6H). **^{13}C NMR (DMSO-d6, 150MHz)**, δ (ppm): 161.95, 147.62. 146.09, 138.07. 137.15, 131.28, 128.17, 125.44, 123.79, 121.11 (2C), 112.50 (2C),40.39 (2C). **LRMS (ESI)**: Expected mass for $C_{15}H_{14}ClN_3O_3$ $(M + H)^+$: 320.07 Da, found 319.67 Da.

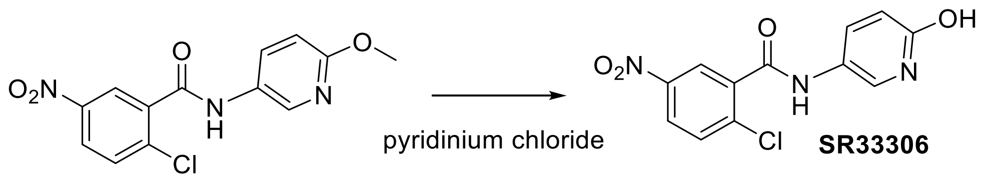

SR33306

2-chloro-N-(6-hydroxypyridin-3-yl)-5-nitrobenzamide

The mixture of 2-chloro-N-(6-methoxypyridin-3-yl)-5-nitrobenzamide (0.129 g, 0.42 mmol) and pyridinium chloride (5.3 g, 46.2 mmol) was heated at 150°C oil bath for 30 min. The reaction was cool to room temperature before being quenched with water, followed by addition of EtOAc. The organic layer was separated and washed with sat'd $NaHCO_3$ and brine and dried over $MgSO_4$, filtered, and concentrated under reduced pressure. The crude material was purified by silica gel chromatography to yield the title compound as a white solid. **^{1}HNMR (DMSO-d6, 600 MHz)**, δ (ppm): 11.52 (broad s, 1H), 10.51 (s, 1H), 8.51 (d, J=4.0 Hz, 1H), 8.37 (dd, J=8.0, 4.0 Hz, 1H), 8.0 (d, J=4.0 Hz, 1H), 7.92 (d, J= 8.0 Hz, 1H), 7.58 (dd, J=8.0 Hz, 4.0 Hz, 1H), 6.45 (d, J=4.0 Hz,1H). **^{13}C NMR (DMSO-d6, 150 MHz)**, δ (ppm): 162.43, 160.60, 146.10, 137.22, 137.13, 136.33, 131.38 (2C), 125.81, 123.97(2C), 119.58. **LCMS (ESI)**: Expected mass for $C_{12}H_8ClN_3O_4$ $(M + H)^+$: 294.03 Da, found 293.49 Da.

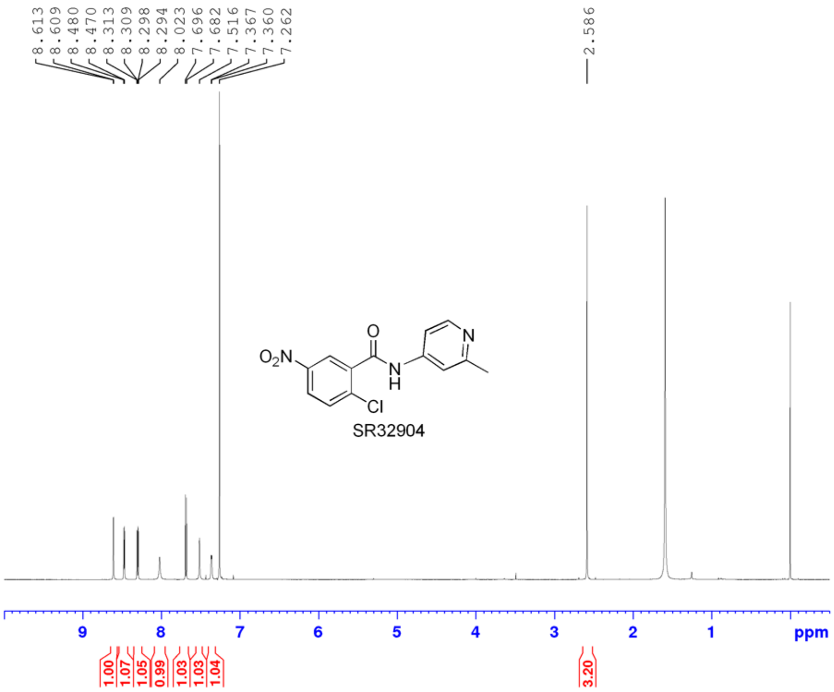

Current Data Parameters
NAME zd-SR32904
EXPNO 10
PROCNO 1

F2 - Acquisition Parameters
Date_ 20220814
Time 15.05 h
INSTRUM CAB AV4 600 MHZ BASIC
PROBHD Z161159_0005 (
PULPROG zg30
TD 65536
SOLVENT CDCl3
NS 16
DS 2
SWH 11904.762 Hz
FIDRES 0.363304 Hz
AQ 2.7525120 sec
RG 19.5602
DW 42.000 usec
DE 14.42 usec
TE 298.1 K
D1 2.00000000 sec
TD0 1
SFO1 600.1837061 MHz
NUC1 1H
P0 2.60 usec
P1 7.80 usec
PLW1 5.68470001 W

F2 - Processing parameters
SI 65536
SF 600.1800134 MHz
WDW EM
SSB 0
LB 0.30 Hz
GB 0
PC 1.00

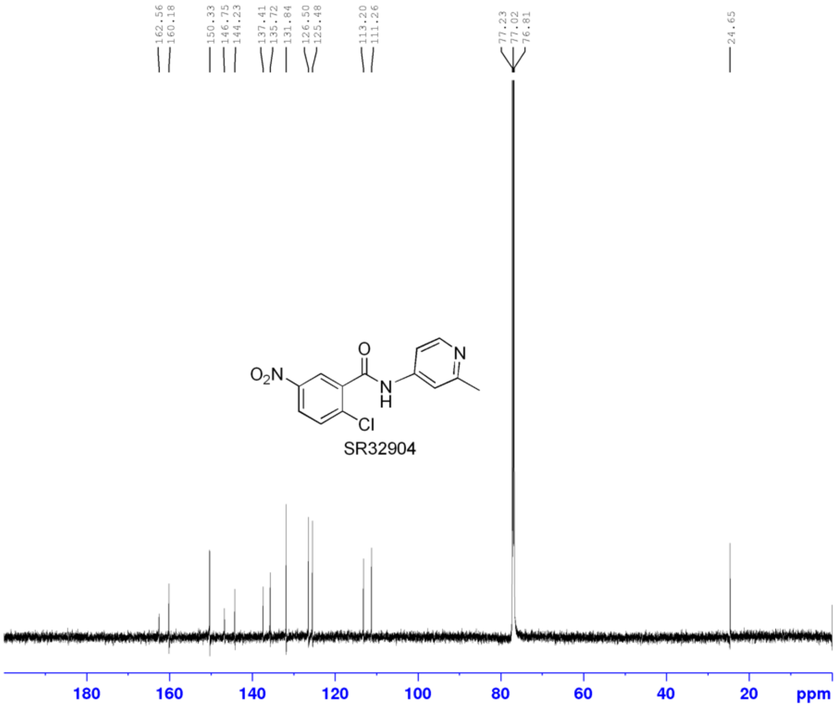

Current Data Parameters
NAME zd-SR32904
EXPNO 11
PROCNO 1

F2 - Acquisition Parameters
Date_ 20220814
Time 15.57 h
INSTRUM CAB AV4 600 MHZ BASIC
PROBHD Z161159_0005 (
PULPROG zgpg30
TD 65536
SOLVENT CDCl3
NS 1024
DS 4
SWH 35714.285 Hz
FIDRES 1.089913 Hz
AQ 0.9175040 sec
RG 27.0833
DW 14.000 usec
DE 18.00 usec
TE 298.2 K
D1 2.00000000 sec
D11 0.03000000 sec
TD0 1
SFO1 150.9304726 MHz
NUC1 13C
P0 3.97 usec
P1 11.90 usec
PLW1 87.09600067 W
SFO2 600.1824007 MHz
NUC2 1H
CPDPRG[2 waltz65
PCPD2 70.00 usec
PLW2 5.68470001 W
PLW12 0.07058300 W
PLW13 0.03550300 W

F2 - Processing parameters
SI 32768
SF 150.9153810 MHz
WDW EM
SSB 0
LB 1.00 Hz
GB 0
PC 1.40

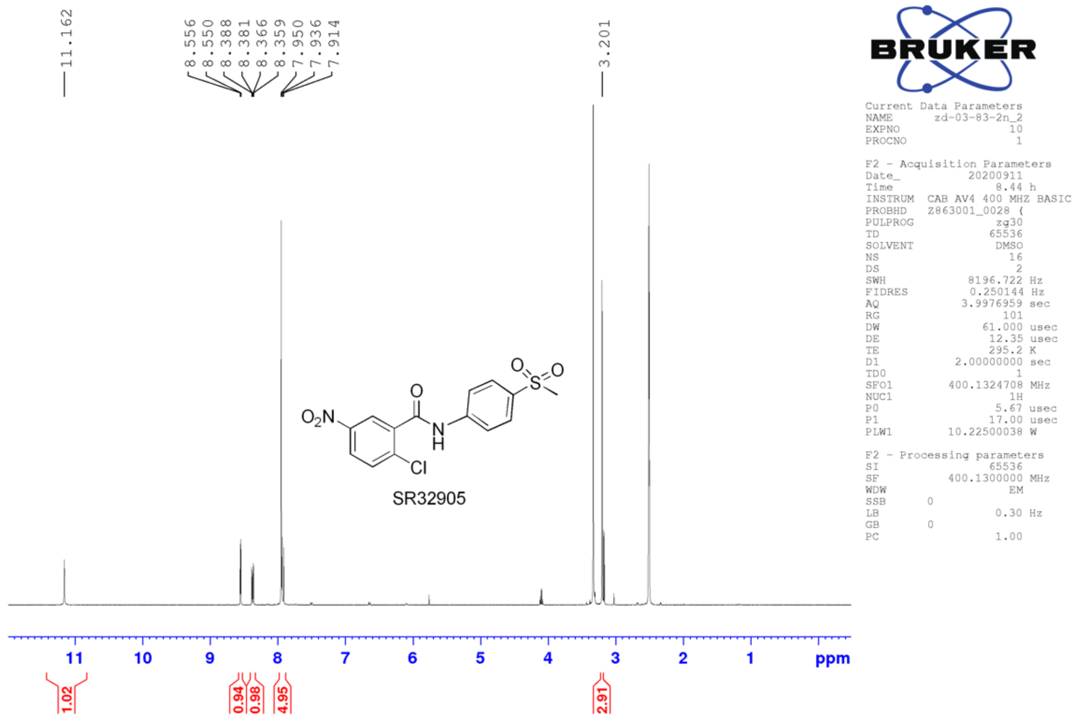
11.162
8.556
8.550
8.388
8.381
8.366
8.359
7.950
7.936
7.914
3.201
SR32905
11 10 9 8 7 6 5 4 3 2 1 ppm
1.02
0.94
0.98
4.95
2.91
BRUKER
Current Data Parameters
NAME zd-03-83-2n_2
EXPNO 10
PROCNO 1
F2 - Acquisition Parameters
Date_ 20200911
Time 8.44 h
INSTRUM CAB AV4 400 MHZ BASIC
PROBHD Z863001_0028 (
PULPROG zg30
TD 65536
SOLVENT DMSO
NS 16
DS 2
SWH 8196.722 Hz
FIDRES 0.250144 Hz
AQ 3.9976959 sec
RG 101
DW 61.000 usec
DE 12.35 usec
TE 295.2 K
D1 2.00000000 sec
TD0 1
SFO1 400.1324708 MHz
NUC1 1H
P0 5.67 usec
P1 17.00 usec
PLW1 10.22500038 W
F2 - Processing parameters
SI 65536
SF 400.1300000 MHz
WDW EM
SSB 0
LB 0.30 Hz
GB 0
PC 1.00

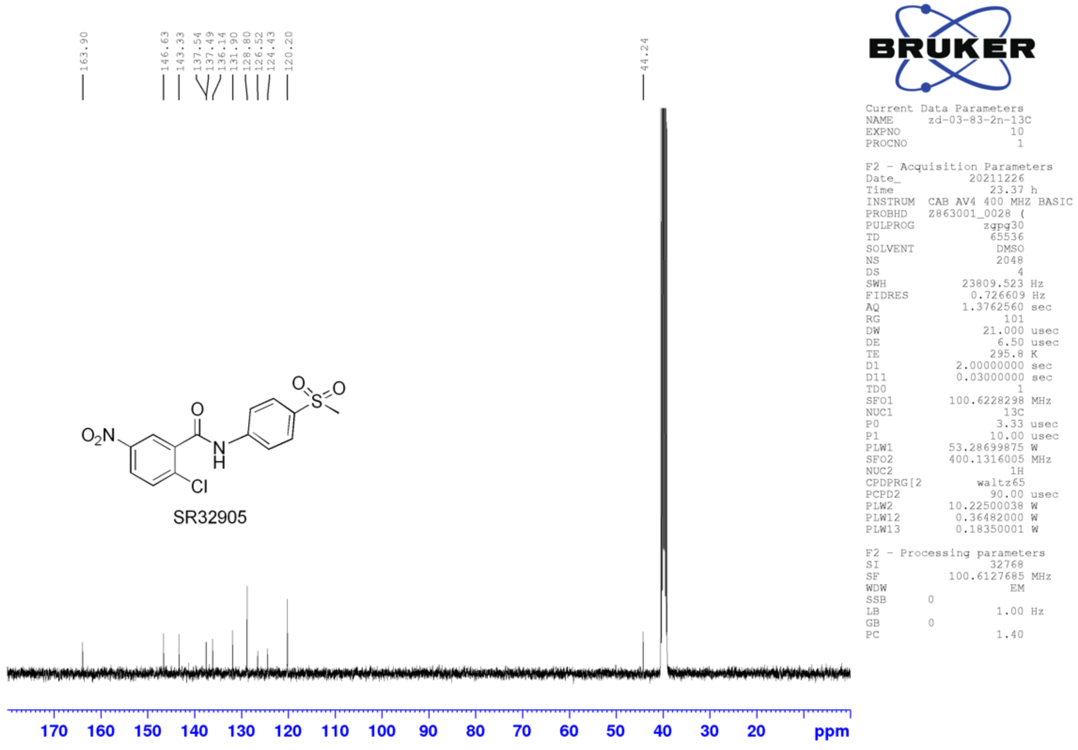
163.90
146.63
143.33
137.54
137.49
136.14
131.90
128.80
126.52
124.43
120.20
44.24
SR32905
170 160 150 140 130 120 110 100 90 80 70 60 50 40 30 20 ppm
BRUKER
Current Data Parameters
NAME zd-03-83-2n-13C
EXPNO 10
PROCNO 1
F2 - Acquisition Parameters
Date_ 20211226
Time 23.37 h
INSTRUM CAB AV4 400 MHZ BASIC
PROBHD Z863001_0028 (
PULPROG zgpg30
TD 65536
SOLVENT DMSO
NS 2048
DS 4
SWH 23809.523 Hz
FIDRES 0.726609 Hz
AQ 1.3762560 sec
RG 101
DW 21.000 usec
DE 6.50 usec
TE 295.8 K
D1 2.00000000 sec
D11 0.03000000 sec
TD0 1
SFO1 100.6228298 MHz
NUC1 13C
P0 3.33 usec
P1 10.00 usec
PLW1 53.28699875 W
SFO2 400.1316005 MHz
NUC2 1H
CPDPRG[2 waltz65
PCPD2 90.00 usec
PLW2 10.22500038 W
PLW12 0.36482000 W
PLW13 0.18350001 W
F2 - Processing parameters
SI 32768
SF 100.6127685 MHz
WDW EM
SSB 0
LB 1.00 Hz
GB 0
PC 1.40

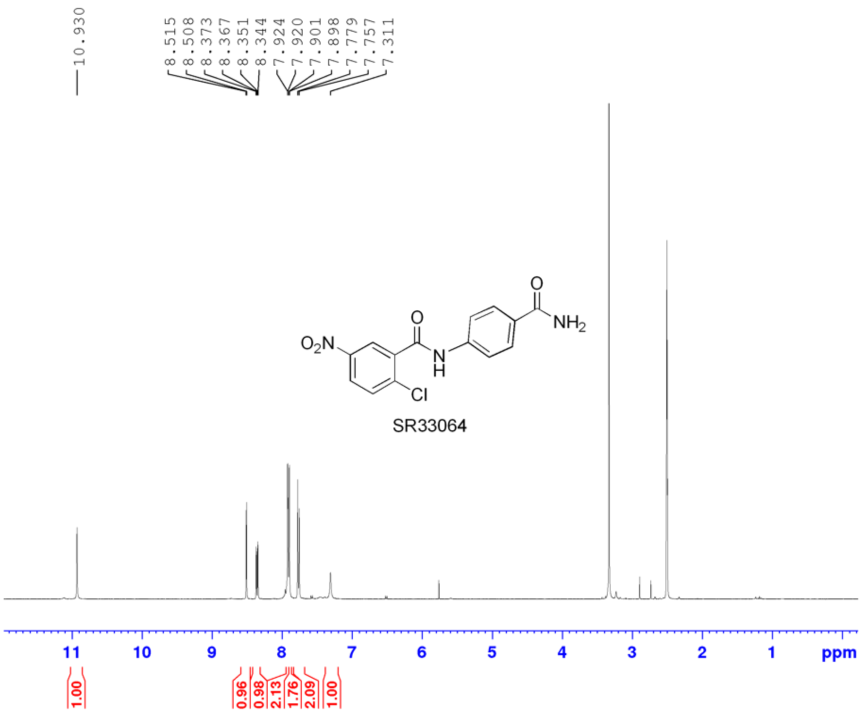

BRUKER

```
Current Data Parameters
NAME          zd-03-96-4n
EXPNO                  10
PROCNO                  1

F2 - Acquisition Parameters
Date_            20200906
Time                18.07 h
INSTRUM  CAB AV4 400 MHZ BASIC
PROBHD   Z863001_0028 (
PULPROG              zg30
TD                  65536
SOLVENT              DMSO
NS                     16
DS                      2
SWH              8196.722 Hz
FIDRES           0.250144 Hz
AQ              3.9976959 sec
RG                    101
DW                 61.000 usec
DE                  12.35 usec
TE                  295.4 K
D1             2.00000000 sec
TD0                     1
SFO1          400.1324708 MHz
NUC1                   1H
P0                   5.67 usec
P1                  17.00 usec
PLW1          10.22500038 W

F2 - Processing parameters
SI                  65536
SF            400.1300000 MHz
WDW                    EM
SSB      0
LB                   0.30 Hz
GB       0
PC                   1.00
```

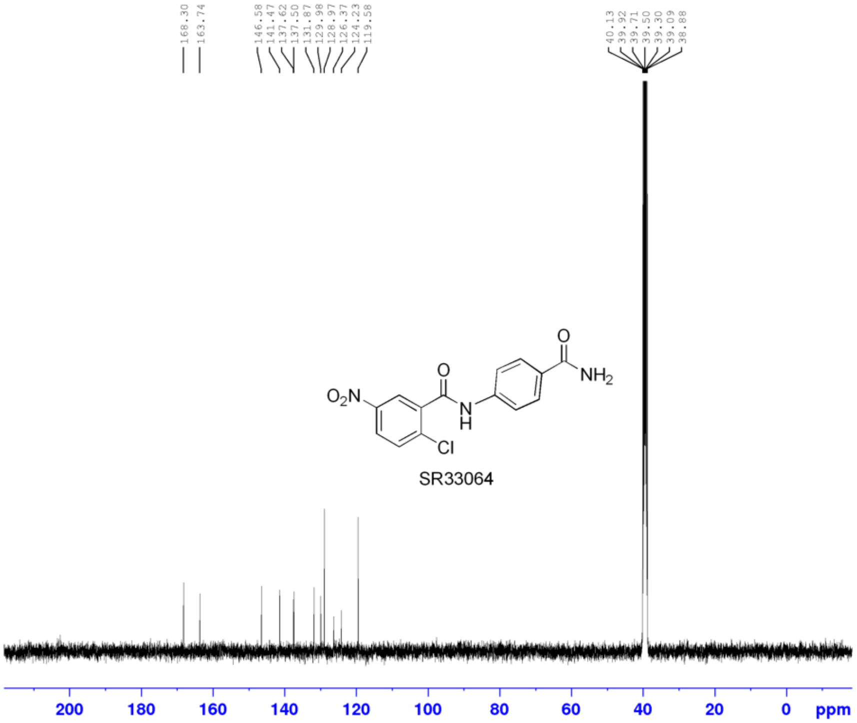

BRUKER

```
Current Data Parameters
NAME         zd-03-96-4-13C
EXPNO                  10
PROCNO                  1

F2 - Acquisition Parameters
Date_            20211226
Time                15.40 h
INSTRUM  CAB AV4 400 MHZ BASIC
PROBHD   Z863001_0028 (
PULPROG            zgpg30
TD                  65536
SOLVENT              DMSO
NS                   2048
DS                      4
SWH             23809.523 Hz
FIDRES           0.726609 Hz
AQ              1.3762560 sec
RG                    101
DW                 21.000 usec
DE                   6.50 usec
TE                  295.7 K
D1             2.00000000 sec
D11            0.03000000 sec
TD0                     1
SFO1          100.6228298 MHz
NUC1                  13C
P0                   3.33 usec
P1                  10.00 usec
PLW1          53.28699875 W
SFO2          400.1316005 MHz
NUC2                   1H
CPDPRG[2           waltz65
PCPD2               90.00 usec
PLW2          10.22500038 W
PLW12          0.36482000 W
PLW13          0.18350001 W

F2 - Processing parameters
SI                  32768
SF            100.6127685 MHz
WDW                    EM
SSB      0
LB                   1.00 Hz
GB       0
PC                   1.40
```

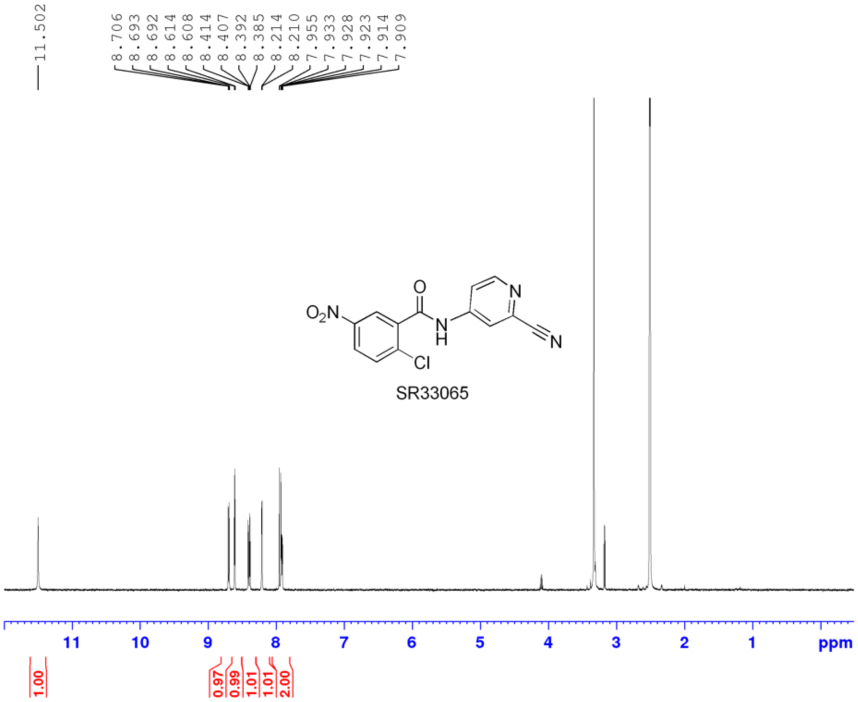

BRUKER

```
Current Data Parameters
NAME          zd-03-96-5n
EXPNO                  10
PROCNO                  1

F2 - Acquisition Parameters
Date_            20200911
Time                 8.40 h
INSTRUM  CAB AV4 400 MHZ BASIC
PROBHD   Z863001_0028 (
PULPROG              zg30
TD                  65536
SOLVENT              DMSO
NS                     16
DS                      2
SWH              8196.722 Hz
FIDRES           0.250144 Hz
AQ              3.9976959 sec
RG                    101
DW                 61.000 usec
DE                  12.35 usec
TE                  295.2 K
D1             2.00000000 sec
TD0                     1
SFO1          400.1324708 MHz
NUC1                   1H
P0                   5.67 usec
P1                  17.00 usec
PLW1          10.22500038 W

F2 - Processing parameters
SI                  65536
SF            400.1300000 MHz
WDW                    EM
SSB      0
LB                   0.30 Hz
GB       0
PC                   1.00
```

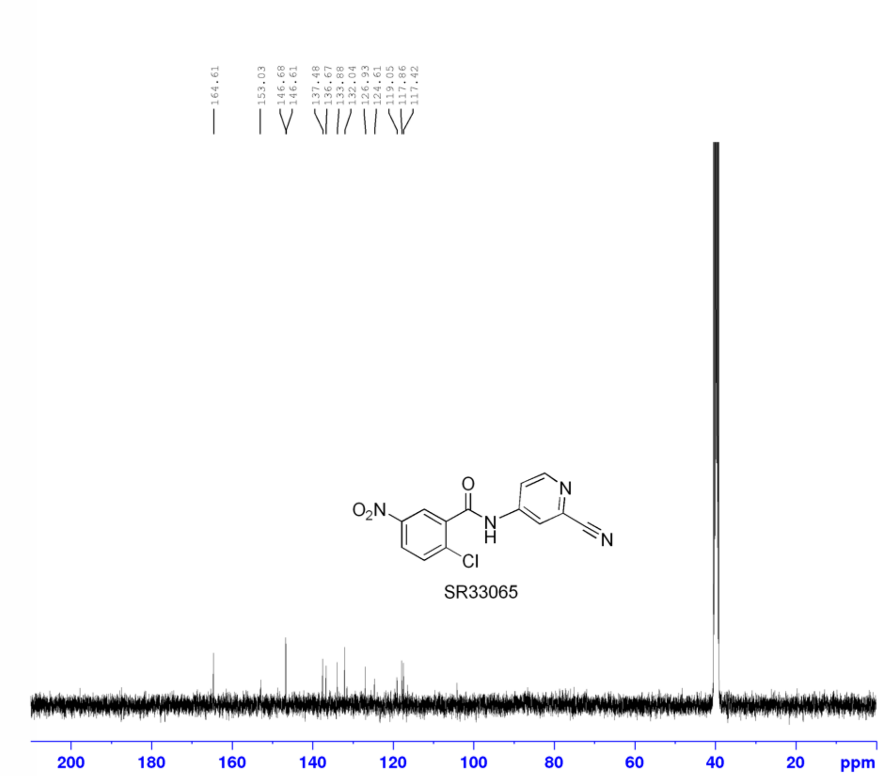

BRUKER

```
Current Data Parameters
NAME      zd-03-96-5-13C
EXPNO                 10
PROCNO                 1

F2 - Acquisition Parameters
Date_           20211227
Time                1.36 h
INSTRUM  CAB AV4 400 MHZ BASIC
PROBHD   Z863001_0028 (
PULPROG           zgpg30
TD                 65536
SOLVENT             DMSO
NS                  2048
DS                     4
SWH            23809.523 Hz
FIDRES          0.726609 Hz
AQ             1.3762560 sec
RG                   101
DW                21.000 usec
DE                  6.50 usec
TE                 295.7 K
D1            2.00000000 sec
D11           0.03000000 sec
TD0                    1
SFO1         100.6228298 MHz
NUC1                 13C
P0                  3.33 usec
P1                 10.00 usec
PLW1         53.28699875 W
SFO2         400.1316005 MHz
NUC2                  1H
CPDPRG[2         waltz65
PCPD2              90.00 usec
PLW2         10.22500038 W
PLW12         0.36482000 W
PLW13         0.18350001 W

F2 - Processing parameters
SI                 32768
SF           100.6127685 MHz
WDW                   EM
SSB      0
LB                  1.00 Hz
GB       0
PC                  1.40
```

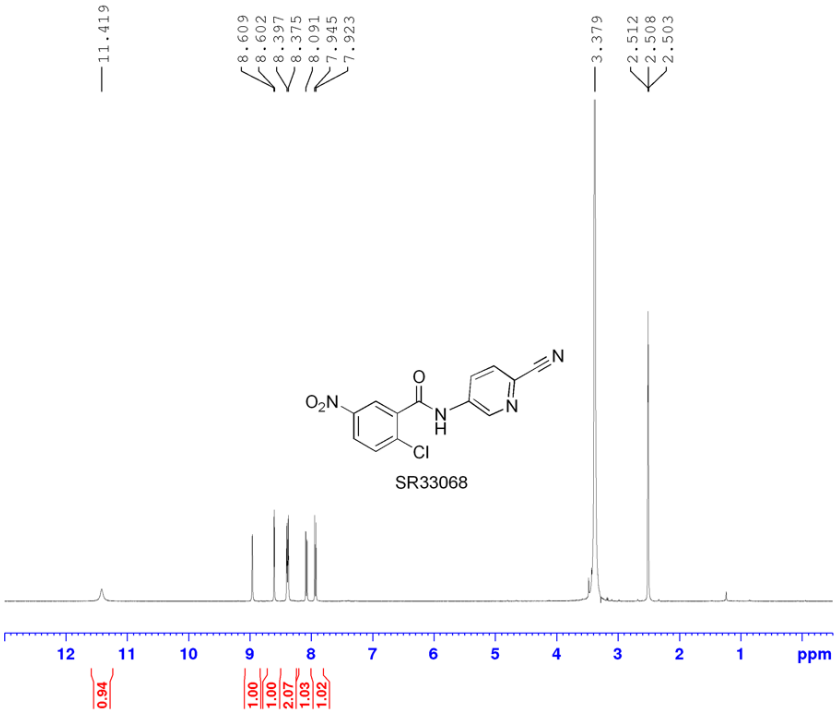


```
Current Data Parameters
NAME          zd-SR33068
EXPNO                 10
PROCNO                 1

F2 - Acquisition Parameters
Date_           20220814
Time               16.37 h
INSTRUM  CAB AV4 400 MHZ BASIC
PROBHD   Z863001_0028 (
PULPROG             zg30
TD                 65536
SOLVENT             DMSO
NS                    16
DS                     2
SWH             8196.722 Hz
FIDRES          0.250144 Hz
AQ             3.9976959 sec
RG                   101
DW                61.000 usec
DE                 12.35 usec
TE                 295.7 K
D1            2.00000000 sec
TD0                    1
SFO1         400.1324708 MHz
NUC1                  1H
P0                  5.67 usec
P1                 17.00 usec
PLW1         10.22500038 W

F2 - Processing parameters
SI                 65536
SF           400.1300000 MHz
WDW                   EM
SSB      0
LB                  0.30 Hz
GB       0
PC                  1.00
```

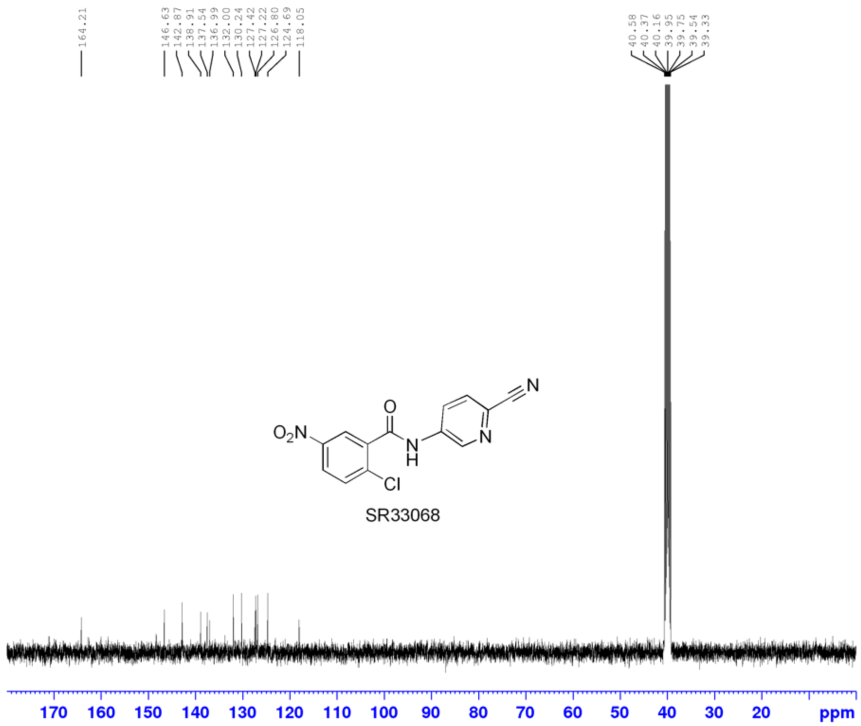


```
Current Data Parameters
NAME          zd-SR33068
EXPNO                 11
PROCNO                 1

F2 - Acquisition Parameters
Date_           20220814
Time               17.06 h
INSTRUM  CAB AV4 400 MHZ BASIC
PROBHD   Z863001_0028 (
PULPROG          zgpg30
TD                 65536
SOLVENT             DMSO
NS                   500
DS                     4
SWH            23809.523 Hz
FIDRES          0.726609 Hz
AQ             1.3762560 sec
RG                   101
DW                21.000 usec
DE                  6.50 usec
TE                 296.8 K
D1            2.00000000 sec
D11           0.03000000 sec
TD0                    1
SFO1         100.6228298 MHz
NUC1                 13C
P0                  3.33 usec
P1                 10.00 usec
PLW1         53.28699875 W
SFO2         400.1316005 MHz
NUC2                  1H
CPDPRG[2         waltz65
PCPD2              90.00 usec
PLW2         10.22500038 W
PLW12         0.36482000 W
PLW13         0.18350001 W

F2 - Processing parameters
SI                 32768
SF           100.6127685 MHz
WDW                   EM
SSB      0
LB                  1.00 Hz
GB       0
PC                  1.40
```

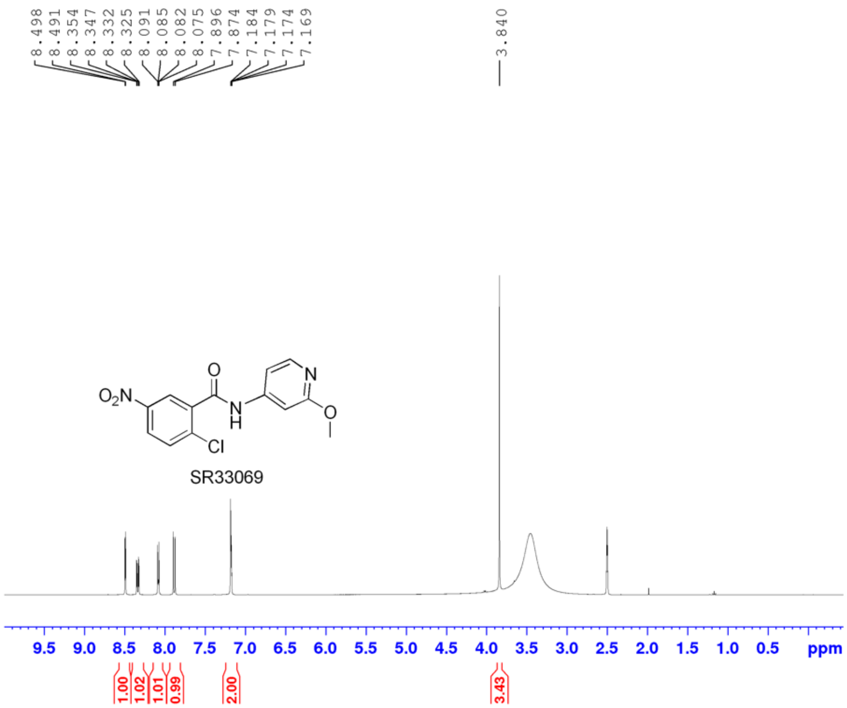


```
Current Data Parameters
NAME          zd-SR33069
EXPNO                 10
PROCNO                 1

F2 - Acquisition Parameters
Date_           20220831
Time               10.29 h
INSTRUM  CAB AV4 400 MHZ BASIC
PROBHD   Z863001_0028 (
PULPROG             zg30
TD                 65536
SOLVENT             DMSO
NS                    16
DS                     2
SWH             8196.722 Hz
FIDRES          0.250144 Hz
AQ             3.9976959 sec
RG                   101
DW                61.000 usec
DE                 12.35 usec
TE                 295.3 K
D1            2.00000000 sec
TD0                    1
SFO1         400.1324708 MHz
NUC1                  1H
P0                  5.67 usec
P1                 17.00 usec
PLW1         10.22500038 W

F2 - Processing parameters
SI                 65536
SF           400.1300030 MHz
WDW                   EM
SSB      0
LB                  0.30 Hz
GB       0
PC                  1.00
```

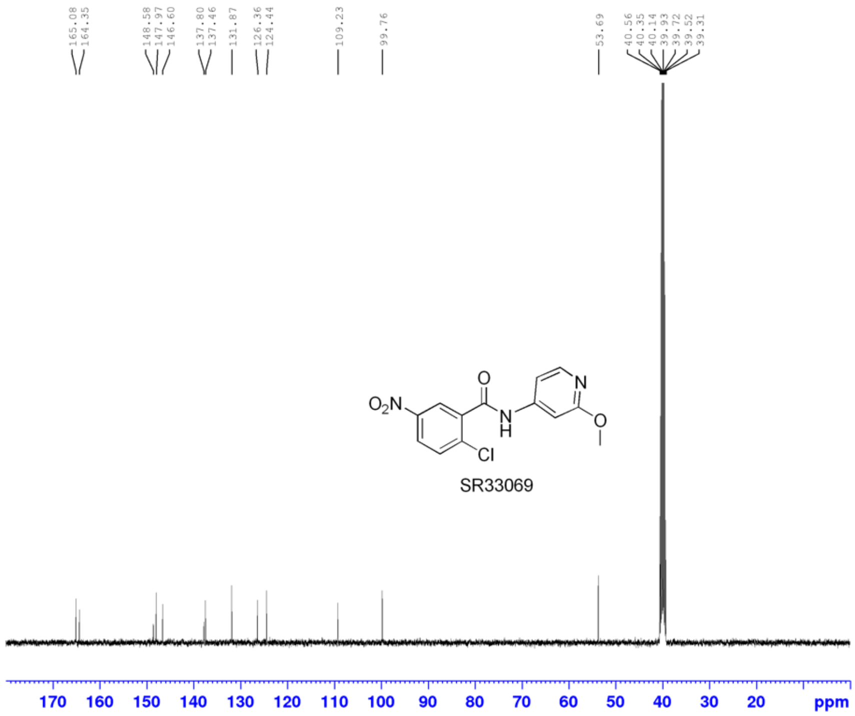


```
Current Data Parameters
NAME          zd-SR33069
EXPNO                 11
PROCNO                 1

F2 - Acquisition Parameters
Date_           20220831
Time               10.53 h
INSTRUM  CAB AV4 400 MHZ BASIC
PROBHD   Z863001_0028 (
PULPROG           zgpg30
TD                 65536
SOLVENT             DMSO
NS                   400
DS                     4
SWH            23809.523 Hz
FIDRES          0.726609 Hz
AQ             1.3762560 sec
RG                   101
DW                21.000 usec
DE                  6.50 usec
TE                 296.4 K
D1            2.00000000 sec
D11           0.03000000 sec
TD0                    1
SFO1         100.6228298 MHz
NUC1                 13C
P0                  3.33 usec
P1                 10.00 usec
PLW1         53.28699875 W
SFO2         400.1316005 MHz
NUC2                  1H
CPDPRG[2         waltz65
PCPD2              90.00 usec
PLW2         10.22500038 W
PLW12         0.36482000 W
PLW13         0.18350001 W

F2 - Processing parameters
SI                 32768
SF           100.6127685 MHz
WDW                   EM
SSB      0
LB                  1.00 Hz
GB       0
PC                  1.40
```

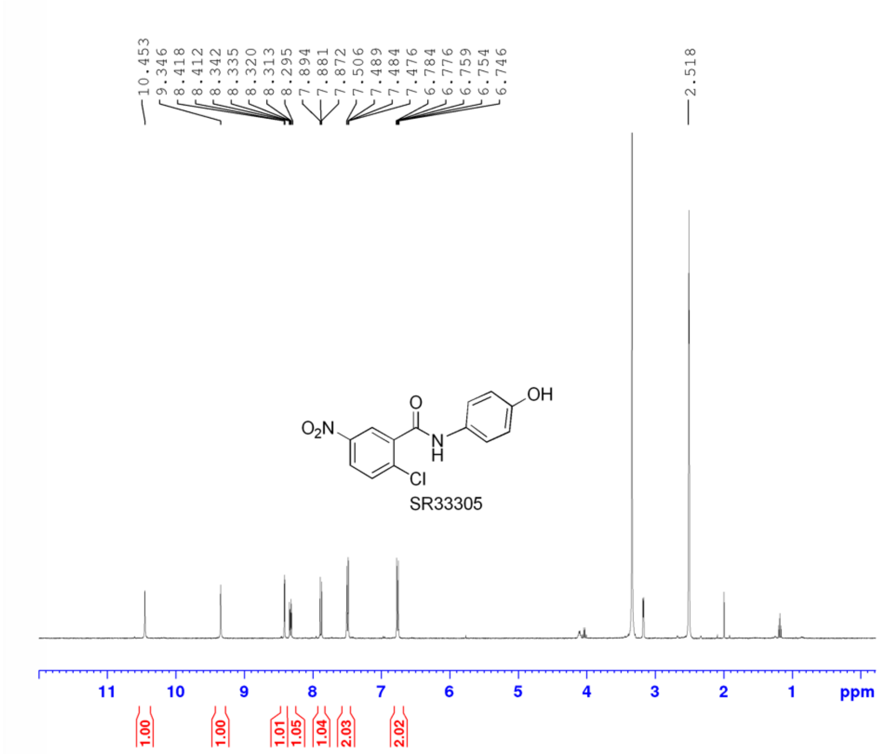


```
Current Data Parameters
NAME          zd-03-99-2n
EXPNO                  10
PROCNO                  1

F2 - Acquisition Parameters
Date_            20200921
Time                11.09
INSTRUM             spect
PROBHD   5 mm PAQNP 13C
PULPROG              zg30
TD                  58188
SOLVENT              DMSO
NS                     16
DS                      2
SWH              7183.908 Hz
FIDRES           0.123460 Hz
AQ              4.0498848 sec
RG                  574.7
DW                 69.600 usec
DE                   6.00 usec
TE                  296.1 K
D1             1.50000000 sec
TD0                     1

======== CHANNEL f1 ========
NUC1                   1H
P1                  15.00 usec
PL1       0 dB
PL1W           9.31909847 W
SFO1          400.1324710 MHz

F2 - Processing parameters
SI                  32768
SF            400.1300000 MHz
WDW                    EM
SSB       0
LB                   0.30 Hz
GB        0
PC                   1.00
```

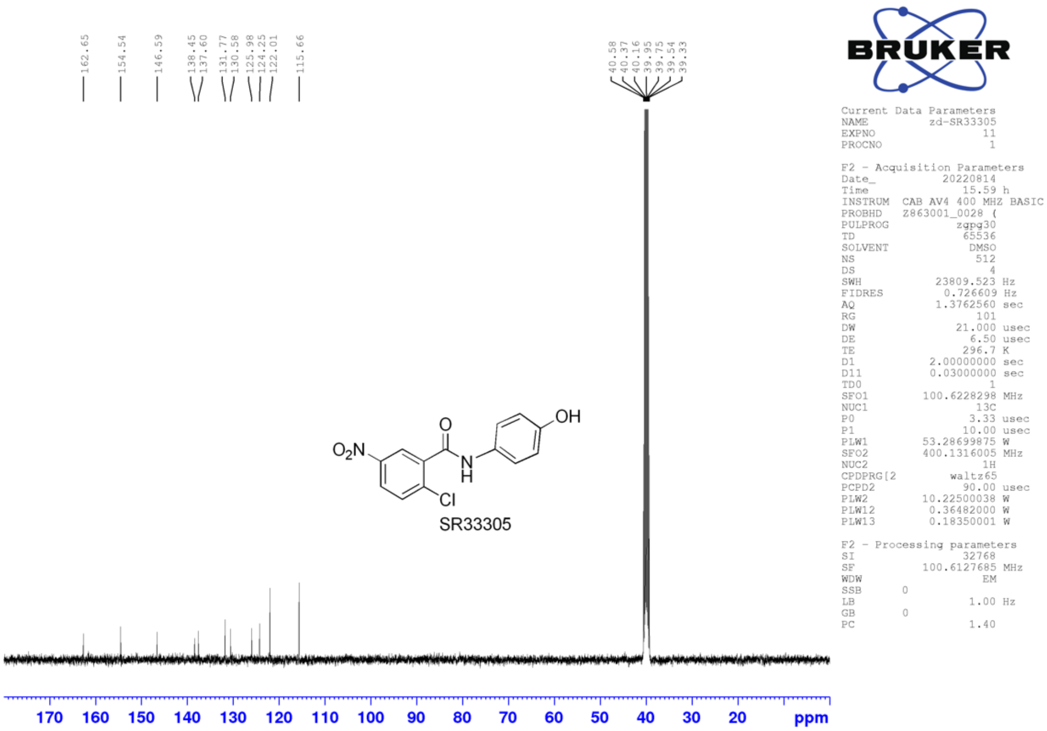
162.65
154.54
146.59
138.45
137.60
131.77
130.58
125.98
124.25
122.01
115.66
40.58
40.37
40.16
39.95
39.75
39.54
39.33
BRUKER
Current Data Parameters
NAME zd-SR33305
EXPNO 11
PROCNO 1
F2 - Acquisition Parameters
Date_ 20220814
Time 15.59 h
INSTRUM CAB AV4 400 MHZ BASIC
PROBHD Z863001_0028 (
PULPROG zgpg30
TD 65536
SOLVENT DMSO
NS 512
DS 4
SWH 23809.523 Hz
FIDRES 0.726609 Hz
AQ 1.3762560 sec
RG 101
DW 21.000 usec
DE 6.50 usec
TE 296.7 K
D1 2.00000000 sec
D11 0.03000000 sec
TD0 1
SFO1 100.6228298 MHz
NUC1 13C
P0 3.33 usec
P1 10.00 usec
PLW1 53.28699875 W
SFO2 400.1316005 MHz
NUC2 1H
CPDPRG[2 waltz65
PCPD2 90.00 usec
PLW2 10.22500038 W
PLW12 0.36482000 W
PLW13 0.18350001 W
F2 - Processing parameters
SI 32768
SF 100.6127685 MHz
WDW EM
SSB 0
LB 1.00 Hz
GB 0
PC 1.40
O_2N
O
N
H
OH
Cl
SR33305
170 160 150 140 130 120 110 100 90 80 70 60 50 40 30 20 ppm

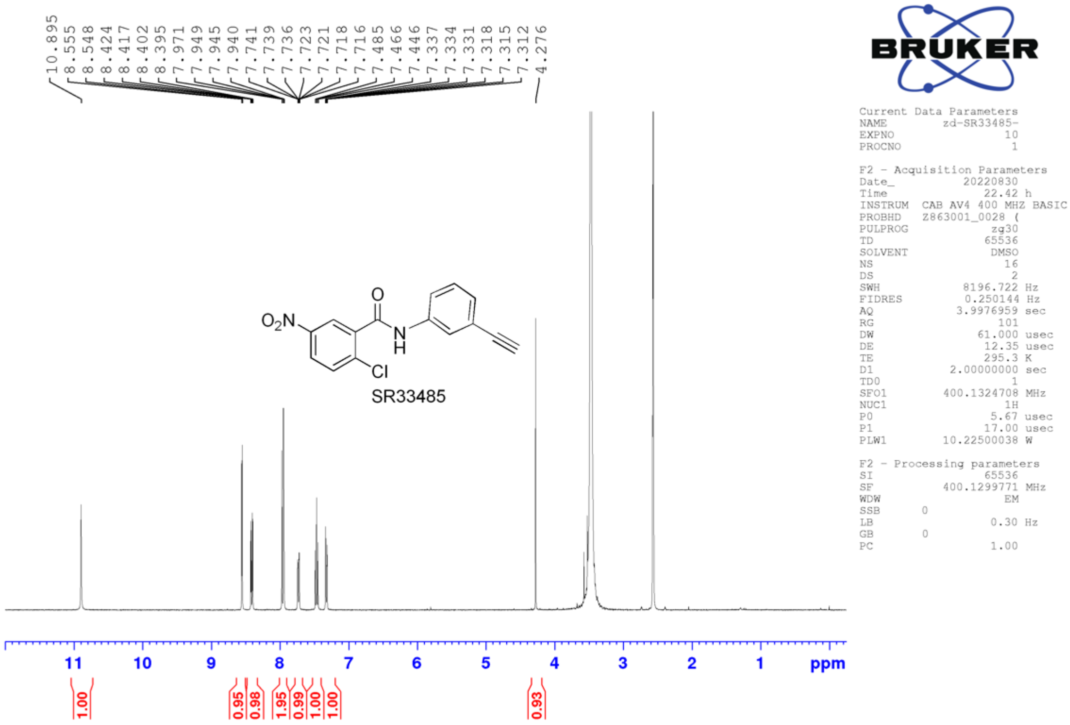
10.895
8.555
8.548
8.424
8.417
8.402
8.395
7.971
7.949
7.945
7.940
7.741
7.739
7.736
7.723
7.721
7.718
7.716
7.485
7.466
7.446
7.337
7.334
7.331
7.318
7.315
7.312
4.276
BRUKER
Current Data Parameters
NAME zd-SR33485-
EXPNO 10
PROCNO 1
F2 - Acquisition Parameters
Date_ 20220830
Time 22.42 h
INSTRUM CAB AV4 400 MHZ BASIC
PROBHD Z863001_0028 (
PULPROG zg30
TD 65536
SOLVENT DMSO
NS 16
DS 2
SWH 8196.722 Hz
FIDRES 0.250144 Hz
AQ 3.9976959 sec
RG 101
DW 61.000 usec
DE 12.35 usec
TE 295.3 K
D1 2.00000000 sec
TD0 1
SFO1 400.1324708 MHz
NUC1 1H
P0 5.67 usec
P1 17.00 usec
PLW1 10.22500038 W
F2 - Processing parameters
SI 65536
SF 400.1299771 MHz
WDW EM
SSB 0
LB 0.30 Hz
GB 0
PC 1.00
O_2N
O
N
H
Cl
SR33485
11 10 9 8 7 6 5 4 3 2 1 ppm
1.00
0.95
0.98
1.95
0.99
1.00
1.00
0.93

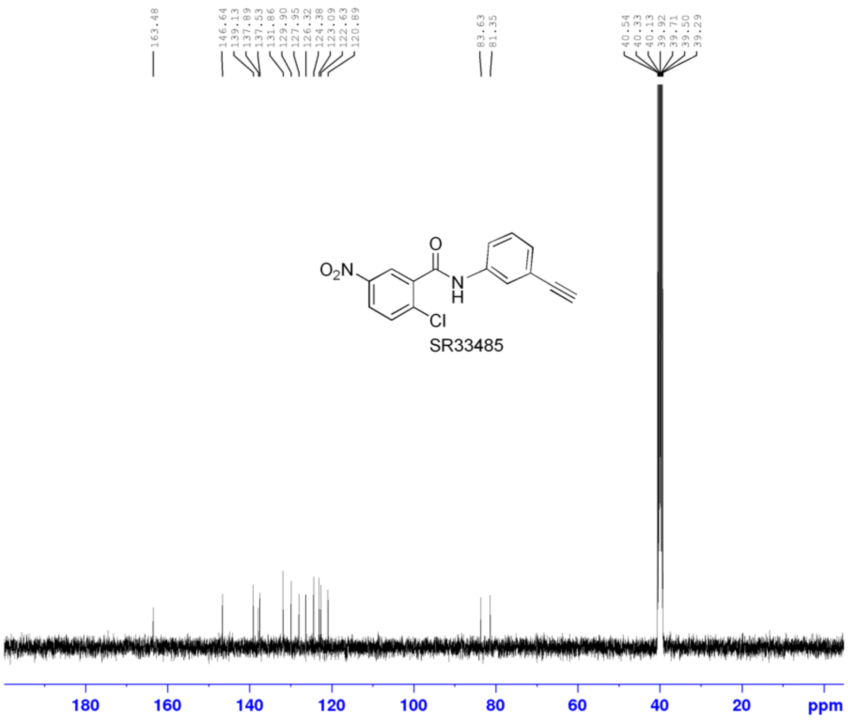


```
Current Data Parameters
NAME          zd-SR33485-
EXPNO                  11
PROCNO                  1

F2 - Acquisition Parameters
Date_            20220830
Time                23.42 h
INSTRUM  CAB AV4 400 MHZ BASIC
PROBHD   Z863001_0028 (
PULPROG            zgpg30
TD                  65536
SOLVENT              DMSO
NS                   1024
DS                      4
SWH             23809.523 Hz
FIDRES           0.726609 Hz
AQ              1.3762560 sec
RG                    101
DW                 21.000 usec
DE                   6.50 usec
TE                  296.3 K
D1             2.00000000 sec
D11            0.03000000 sec
TD0                     1
SFO1          100.6228298 MHz
NUC1                  13C
P0                   3.33 usec
P1                  10.00 usec
PLW1          53.28699875 W
SFO2          400.1316005 MHz
NUC2                   1H
CPDPRG[2          waltz65
PCPD2               90.00 usec
PLW2          10.22500038 W
PLW12          0.36482000 W
PLW13          0.18350001 W

F2 - Processing parameters
SI                  32768
SF            100.6127685 MHz
WDW                    EM
SSB      0
LB                   1.00 Hz
GB       0
PC                   1.40
```

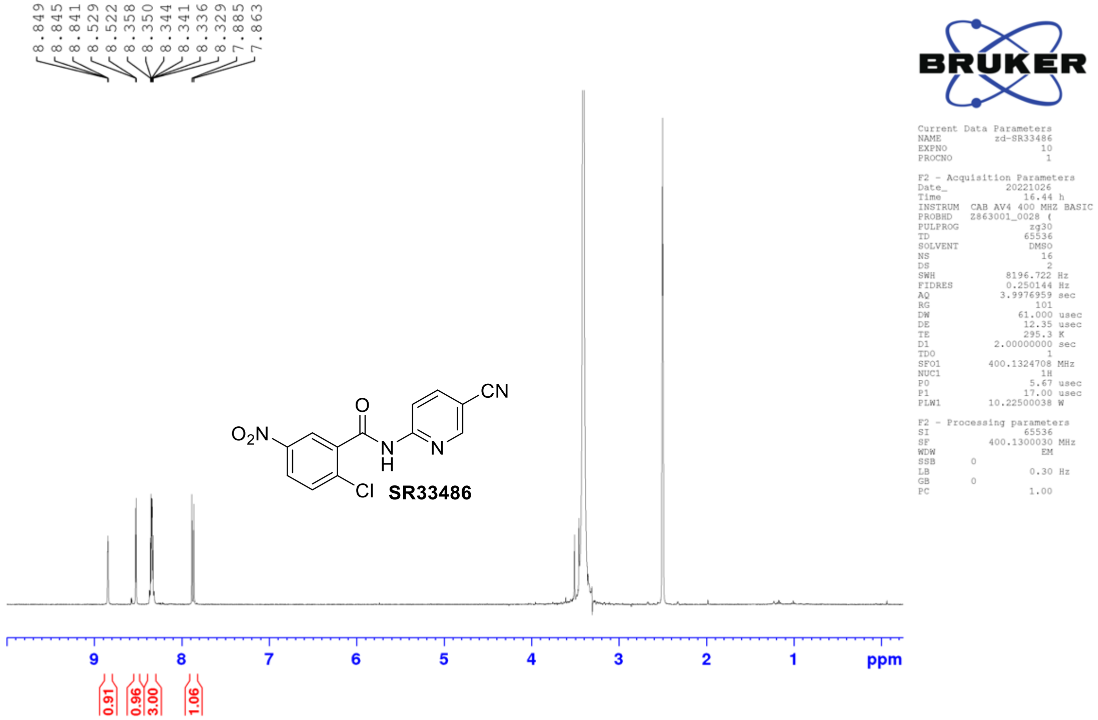
8.849
8.845
8.841
8.529
8.522
8.358
8.350
8.344
8.341
8.336
8.329
7.885
7.863
O
CN
O_2N
N
H
N
Cl
SR33486
9
8
7
6
5
4
3
2
1
ppm
0.91
0.96
3.00
1.06
BRUKER
Current Data Parameters
NAME zd-SR33486
EXPNO 10
PROCNO 1
F2 - Acquisition Parameters
Date_ 20221026
Time 16.44 h
INSTRUM CAB AV4 400 MHZ BASIC
PROBHD Z863001_0028 (
PULPROG zg30
TD 65536
SOLVENT DMSO
NS 16
DS 2
SWH 8196.722 Hz
FIDRES 0.250144 Hz
AQ 3.9976959 sec
RG 101
DW 61.000 usec
DE 12.35 usec
TE 295.3 K
D1 2.00000000 sec
TD0 1
SFO1 400.1324708 MHz
NUC1 1H
P0 5.67 usec
P1 17.00 usec
PLW1 10.22500038 W
F2 - Processing parameters
SI 65536
SF 400.1300030 MHz
WDW EM
SSB 0
LB 0.30 Hz
GB 0
PC 1.00

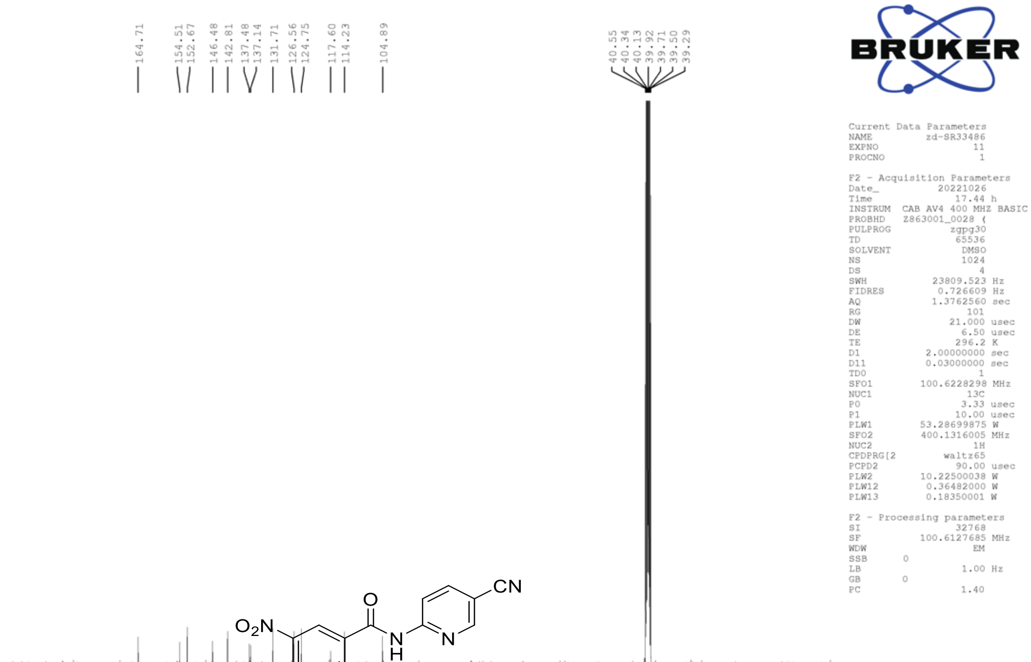
164.71
154.51
152.67
146.48
142.81
137.48
137.14
131.71
126.56
124.75
117.60
114.23
104.89
40.55
40.34
40.13
39.92
39.71
39.50
39.29
O
CN
O_2N
N
H
N
BRUKER
Current Data Parameters
NAME zd-SR33486
EXPNO 11
PROCNO 1
F2 - Acquisition Parameters
Date_ 20221026
Time 17.44 h
INSTRUM CAB AV4 400 MHZ BASIC
PROBHD Z863001_0028 (
PULPROG zgpg30
TD 65536
SOLVENT DMSO
NS 1024
DS 4
SWH 23809.523 Hz
FIDRES 0.726609 Hz
AQ 1.3762560 sec
RG 101
DW 21.000 usec
DE 6.50 usec
TE 296.2 K
D1 2.00000000 sec
D11 0.03000000 sec
TD0 1
SFO1 100.6228298 MHz
NUC1 13C
P0 3.33 usec
P1 10.00 usec
PLW1 53.28699875 W
SFO2 400.1316005 MHz
NUC2 1H
CPDPRG[2 waltz65
PCPD2 90.00 usec
PLW2 10.22500038 W
PLW12 0.36482000 W
PLW13 0.18350001 W
F2 - Processing parameters
SI 32768
SF 100.6127685 MHz
WDW EM
SSB 0
LB 1.00 Hz
GB 0
PC 1.40

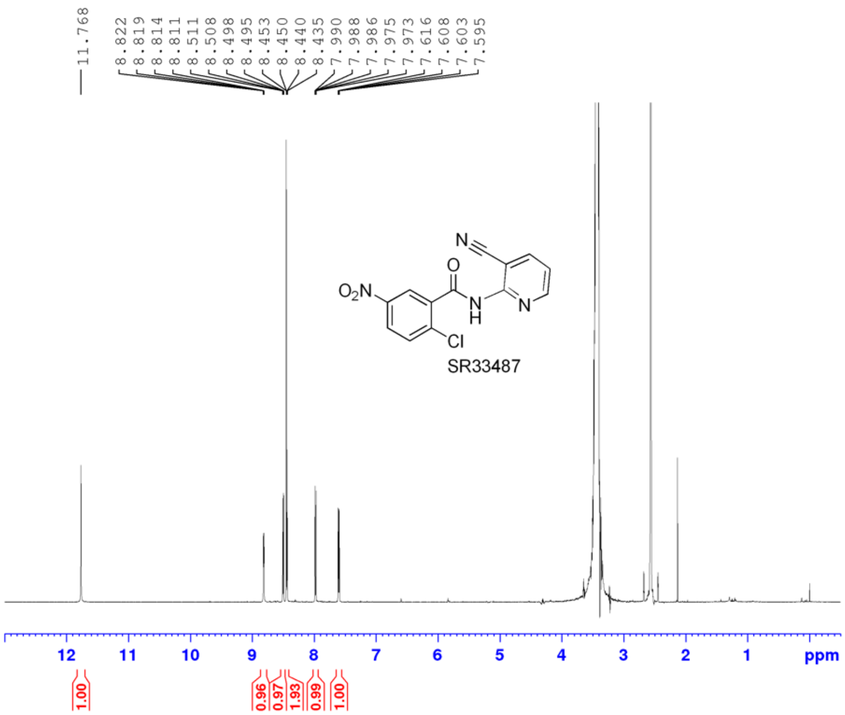

Current Data Parameters
NAME zd-SR33487
EXPNO 10
PROCNO 1

F2 - Acquisition Parameters
Date_ 20220815
Time 11.44 h
INSTRUM CAB AV4 600 MHZ BASIC
PROBHD Z161159_0005 (
PULPROG zg30
TD 65536
SOLVENT DMSO
NS 16
DS 2
SWH 11904.762 Hz
FIDRES 0.363304 Hz
AQ 2.7525120 sec
RG 19.5602
DW 42.000 usec
DE 14.42 usec
TE 298.1 K
D1 2.00000000 sec
TD0 1
SFO1 600.1837061 MHz
NUC1 1H
P0 2.60 usec
P1 7.80 usec
PLW1 5.68470001 W

F2 - Processing parameters
SI 65536
SF 600.1799662 MHz
WDW EM
SSB 0
LB 0.30 Hz
GB 0
PC 1.00

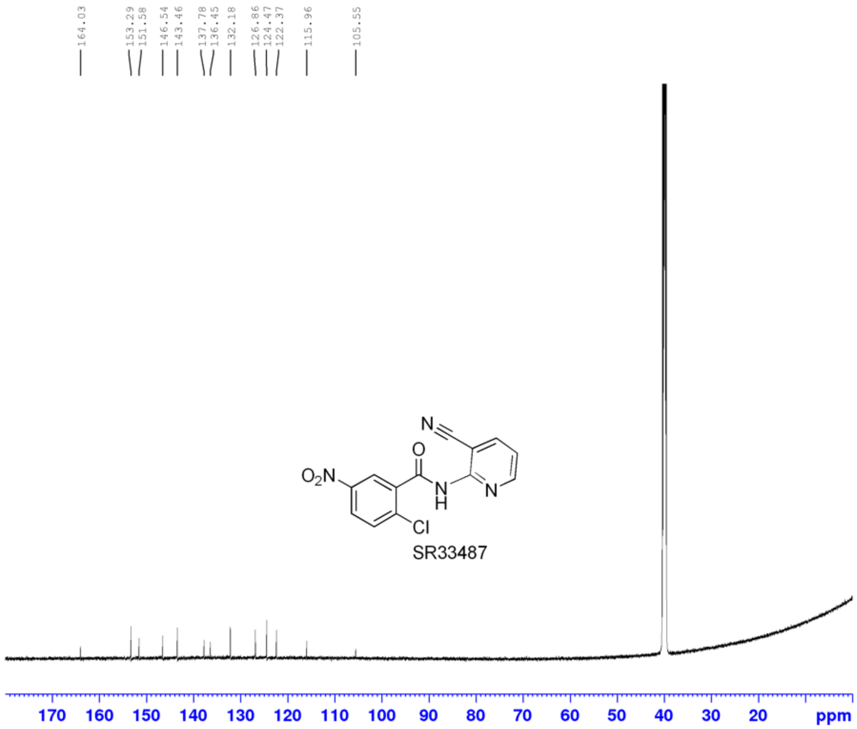

Current Data Parameters
NAME zd-SR33487
EXPNO 11
PROCNO 1

F2 - Acquisition Parameters
Date_ 20220815
Time 15.56 h
INSTRUM CAB AV4 600 MHZ BASIC
PROBHD Z161159_0005 (
PULPROG zgpg30
TD 65536
SOLVENT DMSO
NS 5048
DS 4
SWH 35714.285 Hz
FIDRES 1.089913 Hz
AQ 0.9175040 sec
RG 29.3403
DW 14.000 usec
DE 18.00 usec
TE 298.1 K
D1 2.00000000 sec
D11 0.03000000 sec
TD0 1
SFO1 150.9304726 MHz
NUC1 13C
P0 3.97 usec
P1 11.90 usec
PLW1 87.09600067 W
SFO2 600.1824007 MHz
NUC2 1H
CPDPRG[2 waltz65
PCPD2 70.00 usec
PLW2 5.68470001 W
PLW12 0.07058300 W
PLW13 0.03550300 W

F2 - Processing parameters
SI 32768
SF 150.9153810 MHz
WDW EM
SSB 0
LB 1.00 Hz
GB 0
PC 1.40

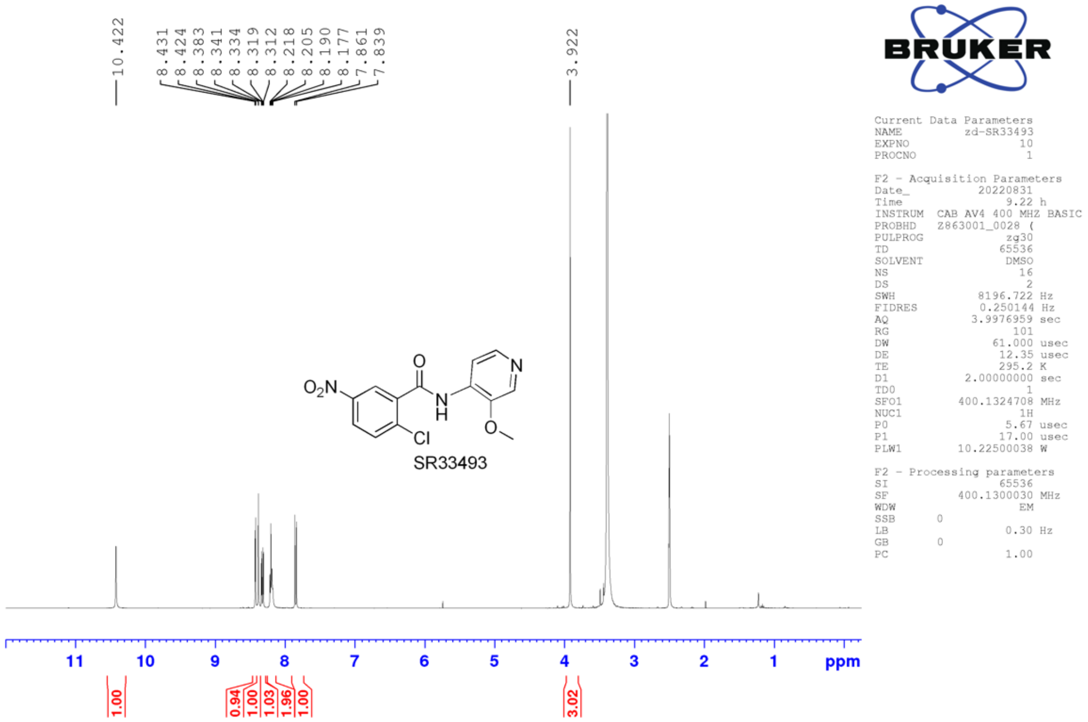
10.422
8.431
8.424
8.383
8.341
8.334
8.319
8.312
8.218
8.205
8.190
8.177
7.861
7.839
3.922
O2N
O
N
H
N
Cl
O
SR33493
11 10 9 8 7 6 5 4 3 2 1 ppm
1.00
0.94
1.00
1.03
1.96
1.00
3.02
BRUKER
Current Data Parameters
NAME zd-SR33493
EXPNO 10
PROCNO 1
F2 - Acquisition Parameters
Date_ 20220831
Time 9.22 h
INSTRUM CAB AV4 400 MHZ BASIC
PROBHD Z863001_0028 (
PULPROG zg30
TD 65536
SOLVENT DMSO
NS 16
DS 2
SWH 8196.722 Hz
FIDRES 0.250144 Hz
AQ 3.9976959 sec
RG 101
DW 61.000 usec
DE 12.35 usec
TE 295.2 K
D1 2.00000000 sec
TD0 1
SFO1 400.1324708 MHz
NUC1 1H
P0 5.67 usec
P1 17.00 usec
PLW1 10.22500038 W
F2 - Processing parameters
SI 65536
SF 400.1300030 MHz
WDW EM
SSB 0
LB 0.30 Hz
GB 0
PC 1.00

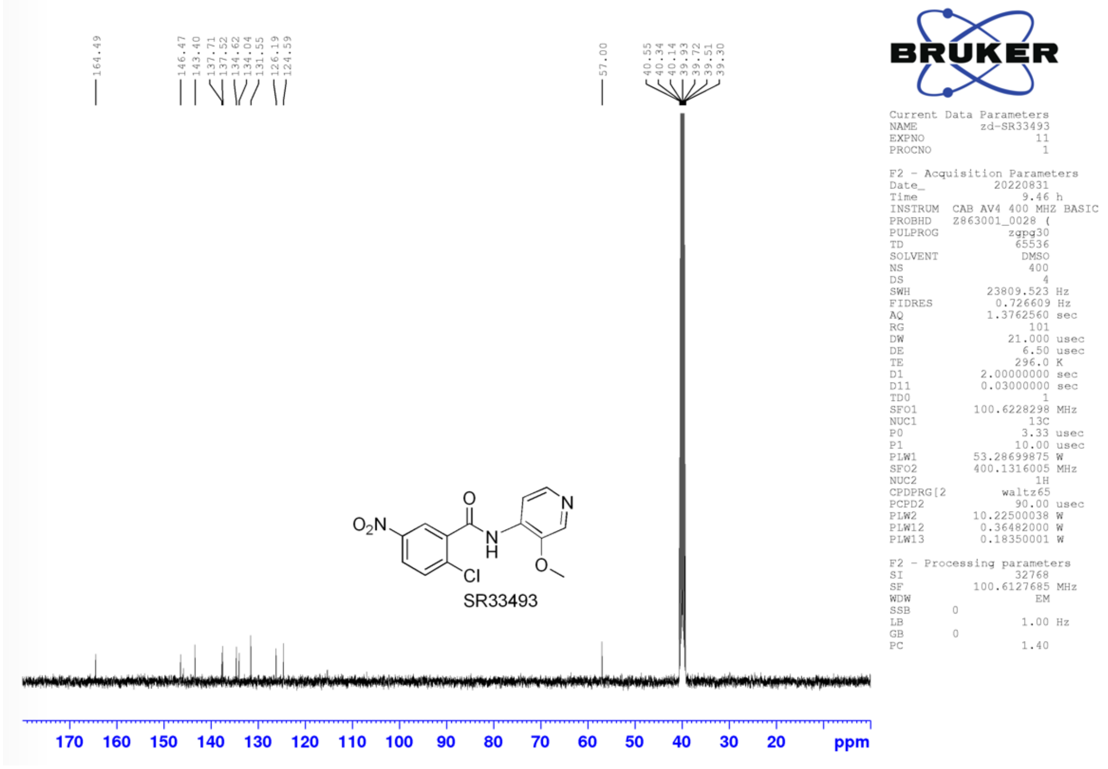
164.49
146.47
143.40
137.71
137.52
134.62
134.04
131.55
126.19
124.59
57.00
40.55
40.34
40.14
39.93
39.72
39.51
39.30
O2N
O
N
H
N
Cl
O
SR33493
170 160 150 140 130 120 110 100 90 80 70 60 50 40 30 20 ppm
BRUKER
Current Data Parameters
NAME zd-SR33493
EXPNO 11
PROCNO 1
F2 - Acquisition Parameters
Date_ 20220831
Time 9.46 h
INSTRUM CAB AV4 400 MHZ BASIC
PROBHD Z863001_0028 (
PULPROG zgpg30
TD 65536
SOLVENT DMSO
NS 400
DS 4
SWH 23809.523 Hz
FIDRES 0.726609 Hz
AQ 1.3762560 sec
RG 101
DW 21.000 usec
DE 6.50 usec
TE 296.0 K
D1 2.00000000 sec
D11 0.03000000 sec
TD0 1
SFO1 100.6228298 MHz
NUC1 13C
P0 3.33 usec
P1 10.00 usec
PLW1 53.28699875 W
SFO2 400.1316005 MHz
NUC2 1H
CPDPRG[2 waltz65
PCPD2 90.00 usec
PLW2 10.22500038 W
PLW12 0.36482000 W
PLW13 0.18350001 W
F2 - Processing parameters
SI 32768
SF 100.6127685 MHz
WDW EM
SSB 0
LB 1.00 Hz
GB 0
PC 1.40

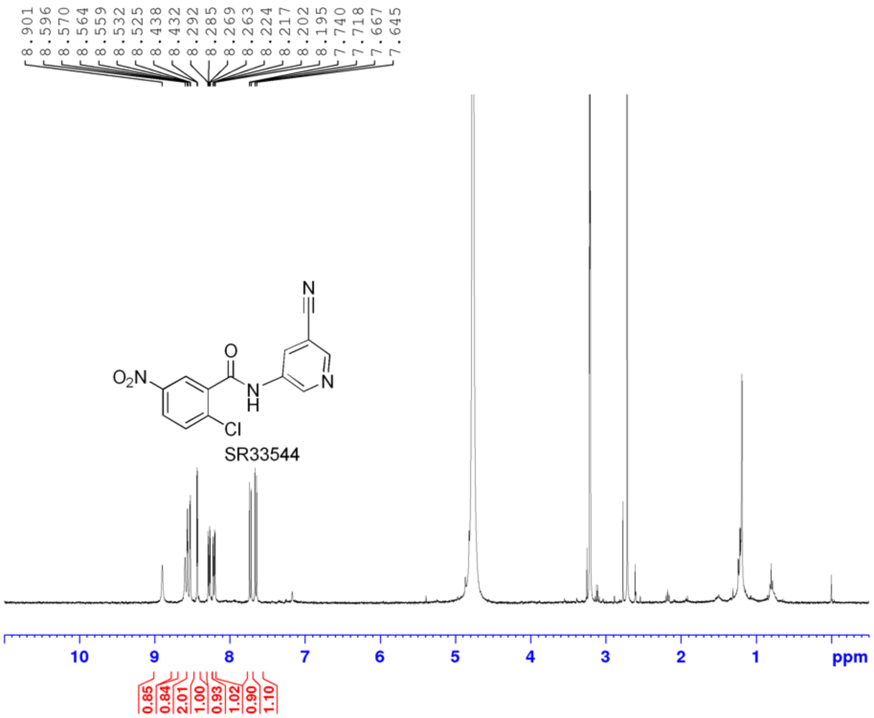


```
Current Data Parameters
NAME          zd-SR33544
EXPNO                 10
PROCNO                 1

F2 - Acquisition Parameters
Date_           20220831
Time               11.35 h
INSTRUM  CAB AV4 400 MHZ BASIC
PROBHD   Z863001_0028 (
PULPROG             zg30
TD                 65536
SOLVENT             MeOD
NS                    16
DS                     2
SWH             8196.722 Hz
FIDRES          0.250144 Hz
AQ             3.9976959 sec
RG                   101
DW                61.000 usec
DE                 12.35 usec
TE                 295.3 K
D1            2.00000000 sec
TD0                    1
SFO1         400.1324708 MHz
NUC1                  1H
P0                  5.67 usec
P1                 17.00 usec
PLW1         10.22500038 W

F2 - Processing parameters
SI                 65536
SF           400.1300476 MHz
WDW                   EM
SSB      0
LB                  0.30 Hz
GB       0
PC                  1.00
```

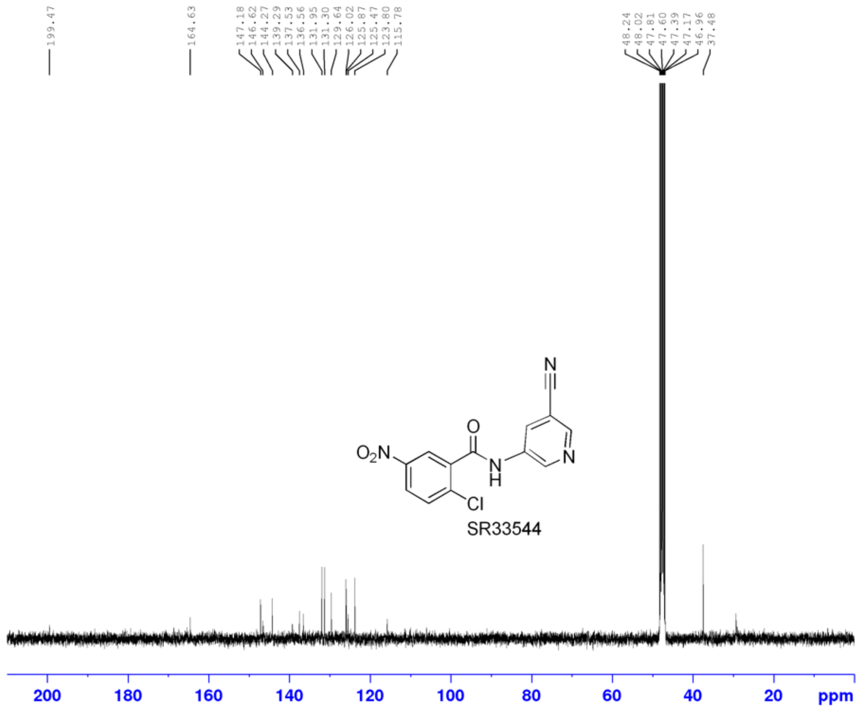


```
Current Data Parameters
NAME          zd-SR33544
EXPNO                 21
PROCNO                 1

F2 - Acquisition Parameters
Date_           20220831
Time               19.54 h
INSTRUM  CAB AV4 400 MHZ BASIC
PROBHD   Z863001_0028 (
PULPROG          zgpg30
TD                 65536
SOLVENT             MeOD
NS                  3042
DS                     4
SWH            23809.523 Hz
FIDRES          0.726609 Hz
AQ             1.3762560 sec
RG                   101
DW                21.000 usec
DE                  6.50 usec
TE                 295.8 K
D1            2.00000000 sec
D11           0.03000000 sec
TD0                    1
SFO1         100.6228298 MHz
NUC1                 13C
P0                  3.33 usec
P1                 10.00 usec
PLW1         53.28699875 W
SFO2         400.1316005 MHz
NUC2                  1H
CPDPRG[2          waltz65
PCPD2              90.00 usec
PLW2         10.22500038 W
PLW12         0.36482000 W
PLW13         0.18350001 W

F2 - Processing parameters
SI                 32768
SF           100.6127685 MHz
WDW                   EM
SSB      0
LB                  1.00 Hz
GB       0
PC                  1.40
```

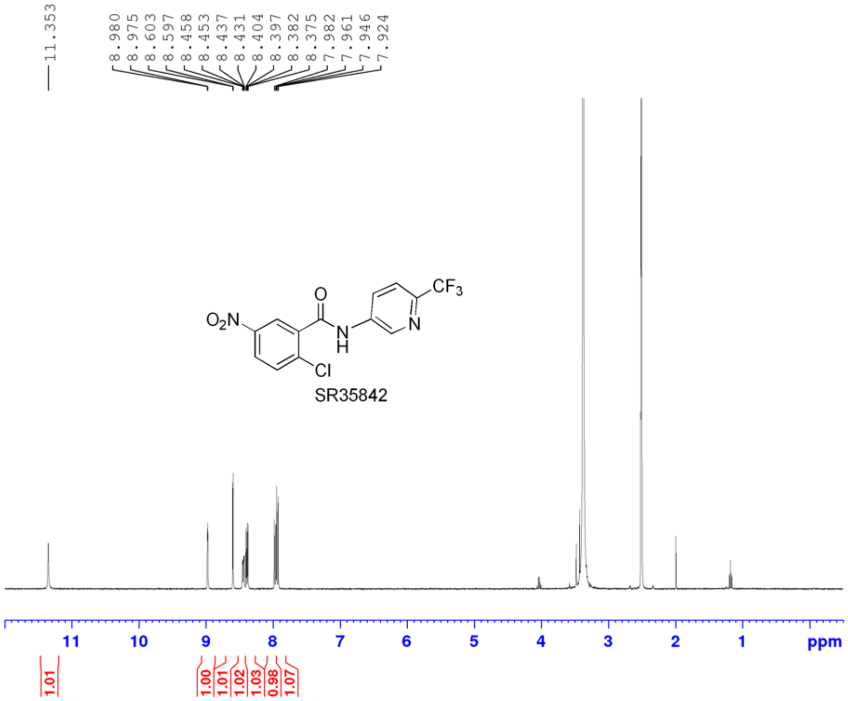

BRUKER

```
Current Data Parameters
NAME          zd-SR35842
EXPNO                 10
PROCNO                 1

F2 - Acquisition Parameters
Date_           20220815
Time             22.38 h
INSTRUM   CAB AV4 400 MHZ BASIC
PROBHD    Z863001_0028 (
PULPROG             zg30
TD                 65536
SOLVENT             DMSO
NS                    16
DS                     2
SWH             8196.722 Hz
FIDRES          0.250144 Hz
AQ             3.9976959 sec
RG                   101
DW                61.000 usec
DE                 12.35 usec
TE                 295.5 K
D1            2.00000000 sec
TD0                    1
SFO1         400.1324708 MHz
NUC1                  1H
P0                  5.67 usec
P1                 17.00 usec
PLW1         10.22500038 W

F2 - Processing parameters
SI                 65536
SF           400.1300000 MHz
WDW                   EM
SSB      0
LB                  0.30 Hz
GB       0
PC                  1.00
```

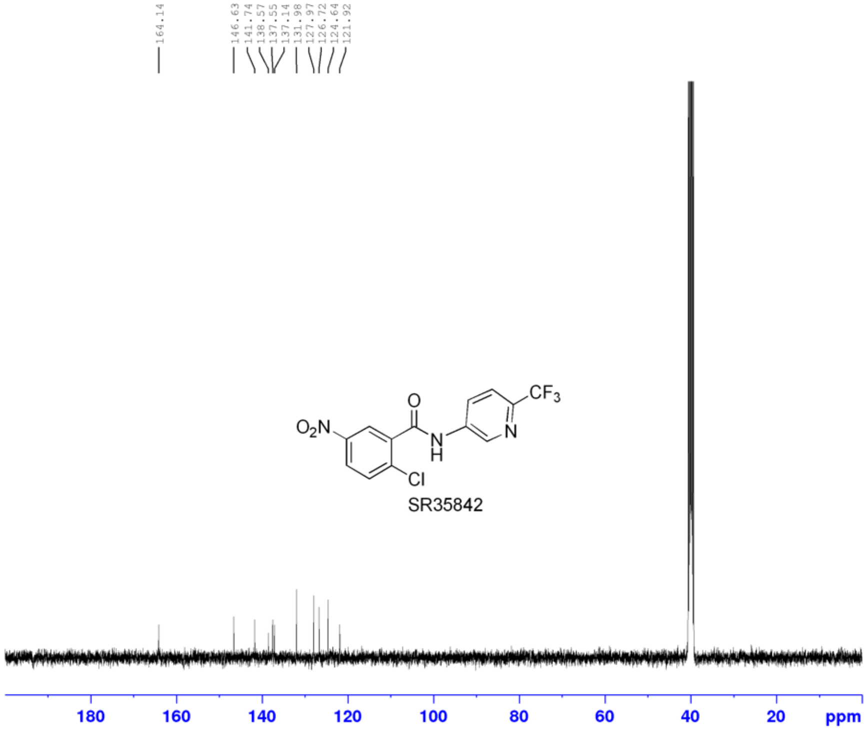

BRUKER

```
Current Data Parameters
NAME          zd-SR35842
EXPNO                 11
PROCNO                 1

F2 - Acquisition Parameters
Date_           20220816
Time              0.36 h
INSTRUM   CAB AV4 400 MHZ BASIC
PROBHD    Z863001_0028 (
PULPROG           zgpg30
TD                 65536
SOLVENT             DMSO
NS                  2048
DS                     4
SWH            23809.523 Hz
FIDRES          0.726609 Hz
AQ             1.3762560 sec
RG                   101
DW                21.000 usec
DE                  6.50 usec
TE                 296.6 K
D1            2.00000000 sec
D11           0.03000000 sec
TD0                    1
SFO1         100.6228298 MHz
NUC1                 13C
P0                  3.33 usec
P1                 10.00 usec
PLW1         53.28699875 W
SFO2         400.1316005 MHz
NUC2                  1H
CPDPRG[2         waltz65
PCPD2              90.00 usec
PLW2         10.22500038 W
PLW12         0.36482000 W
PLW13         0.18350001 W

F2 - Processing parameters
SI                 32768
SF           100.6127685 MHz
WDW                   EM
SSB      0
LB                  1.00 Hz
GB       0
PC                  1.40
```

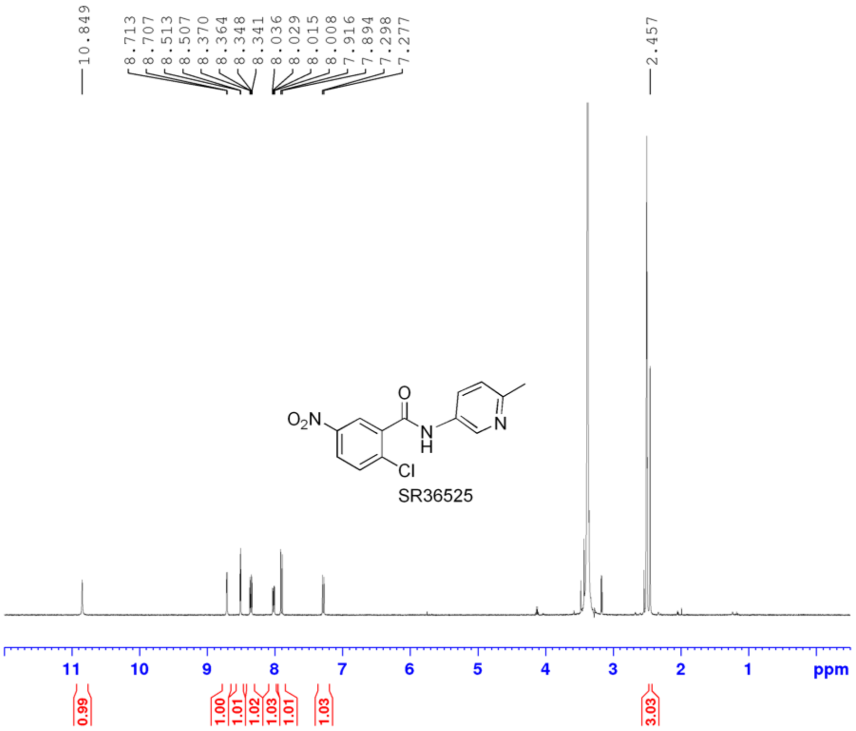


```
Current Data Parameters
NAME        zd-05-118-1
EXPNO                10
PROCNO                1

F2 - Acquisition Parameters
Date_          20211130
Time              15.34 h
INSTRUM  CAB AV4 400 MHZ BASIC
PROBHD   Z863001_0028 (
PULPROG            zg30
TD                65536
SOLVENT            DMSO
NS                   16
DS                    2
SWH            8196.722 Hz
FIDRES         0.250144 Hz
AQ            3.9976959 sec
RG                  101
DW               61.000 usec
DE                12.35 usec
TE                295.2 K
D1           2.00000000 sec
TD0                   1
SFO1        400.1324708 MHz
NUC1                 1H
P0                 5.67 usec
P1                17.00 usec
PLW1        10.22500038 W

F2 - Processing parameters
SI                65536
SF          400.1300000 MHz
WDW                  EM
SSB      0
LB                 0.30 Hz
GB       0
PC                 1.00
```

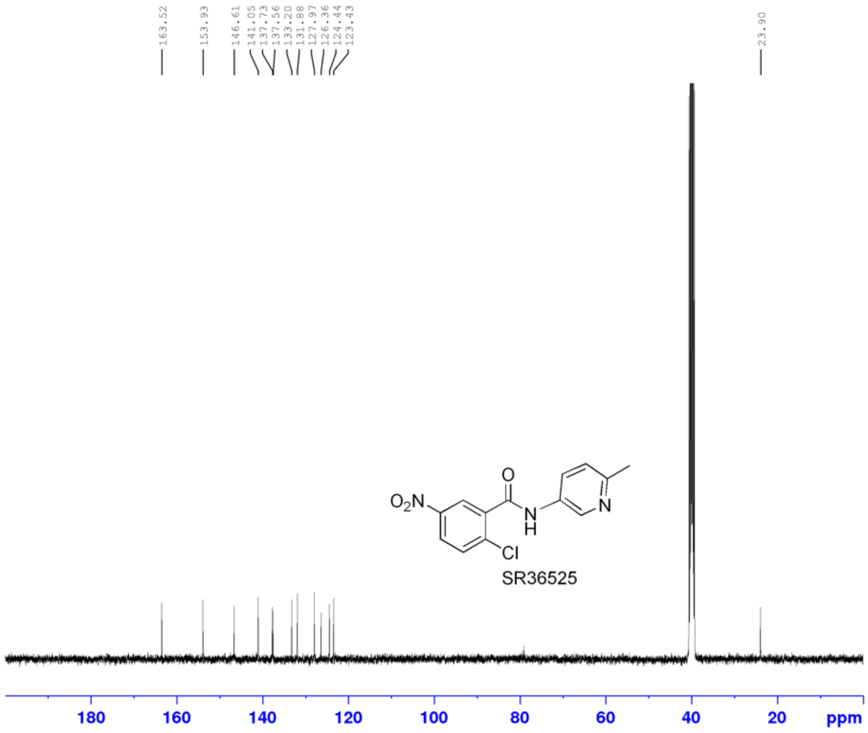


```
Current Data Parameters
NAME          zd-SR36525
EXPNO                 21
PROCNO                 1

F2 - Acquisition Parameters
Date_           20220831
Time               23.52 h
INSTRUM  CAB AV4 400 MHZ BASIC
PROBHD   Z863001_0028 (
PULPROG           zgpg30
TD                 65536
SOLVENT             DMSO
NS                  4096
DS                     4
SWH            23809.523 Hz
FIDRES          0.726609 Hz
AQ             1.3762560 sec
RG                   101
DW                21.000 usec
DE                  6.50 usec
TE                 296.0 K
D1            2.00000000 sec
D11           0.03000000 sec
TD0                    1
SFO1         100.6228298 MHz
NUC1                 13C
P0                  3.33 usec
P1                 10.00 usec
PLW1         53.28699875 W
SFO2         400.1316005 MHz
NUC2                  1H
CPDPRG[2         waltz65
PCPD2              90.00 usec
PLW2         10.22500038 W
PLW12         0.36482000 W
PLW13         0.18350001 W

F2 - Processing parameters
SI                 32768
SF           100.6127685 MHz
WDW                   EM
SSB      0
LB                  1.00 Hz
GB       0
PC                  1.40
```

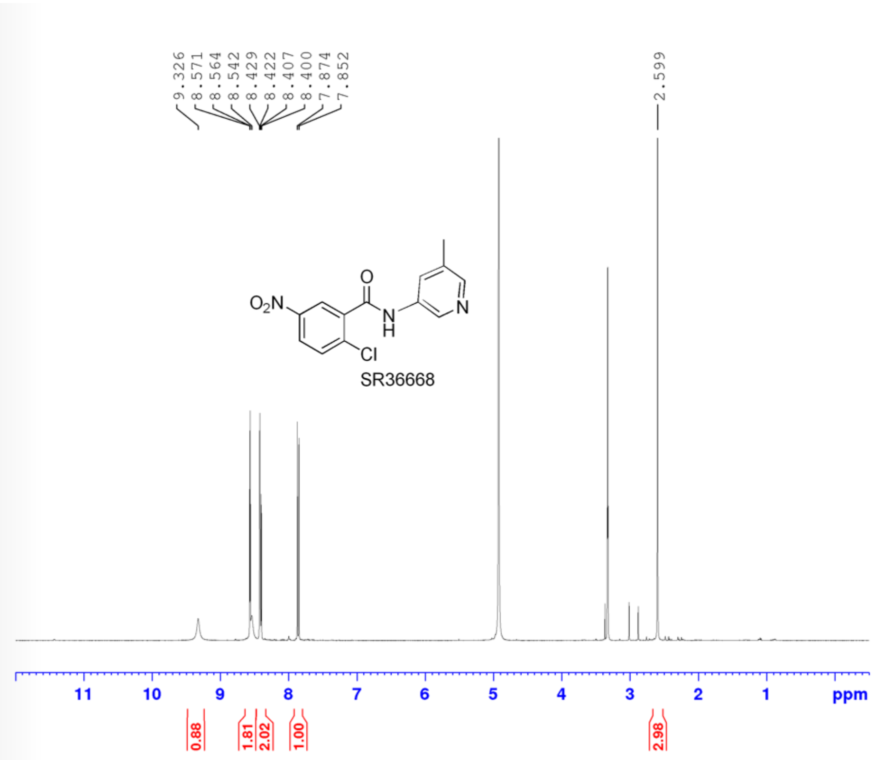

Current Data Parameters

NAME	zd-05-118-3n-RT=1.414
EXPNO	10
PROCNO	1

F2 - Acquisition Parameters

Date_	20220106
Time	18.56 h
INSTRUM	CAB AV4 400 MHZ BASIC
PROBHD	Z863001_0028 (
PULPROG	zg30
TD	65536
SOLVENT	MeOD
NS	16
DS	2
SWH	8196.722 Hz
FIDRES	0.250144 Hz
AQ	3.9976959 sec
RG	101
DW	61.000 usec
DE	12.35 usec
TE	295.3 K
D1	2.00000000 sec
TD0	1
SFO1	400.1324708 MHz
NUC1	1H
P0	5.67 usec
P1	17.00 usec
PLW1	10.22500038 W

F2 - Processing parameters

SI	65536
SF	400.1300000 MHz
WDW	EM
SSB	0
LB	0.30 Hz
GB	0
PC	1.00

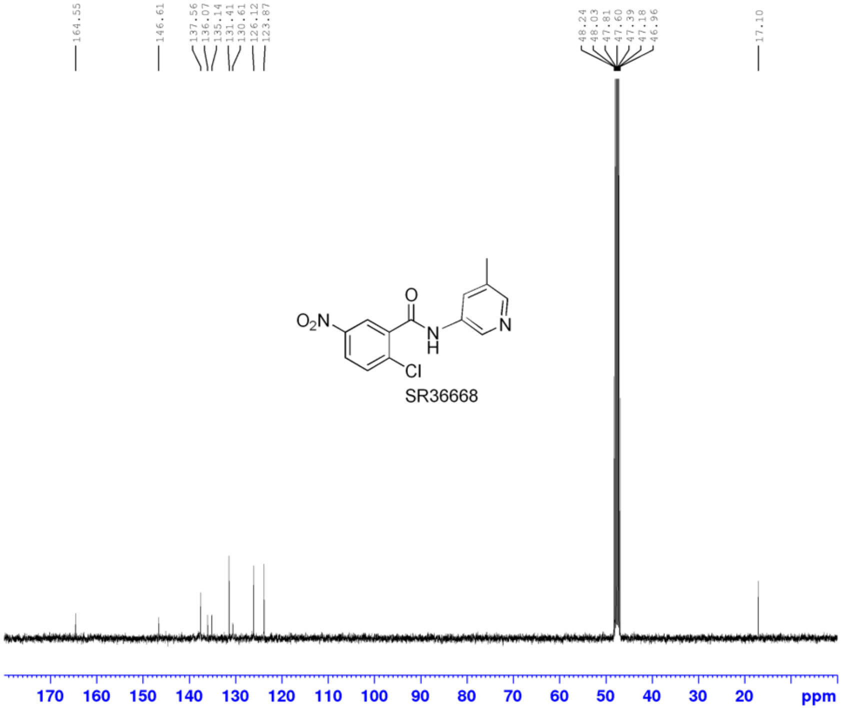

Current Data Parameters

NAME	zd-05-118-3n-RT=1.414
EXPNO	11
PROCNO	1

F2 - Acquisition Parameters

Date_	20220106
Time	19.28 h
INSTRUM	CAB AV4 400 MHZ BASIC
PROBHD	Z863001_0028 (
PULPROG	zgpg30
TD	65536
SOLVENT	MeOD
NS	450
DS	4
SWH	23809.523 Hz
FIDRES	0.726609 Hz
AQ	1.3762560 sec
RG	101
DW	21.000 usec
DE	6.50 usec
TE	296.5 K
D1	2.00000000 sec
D11	0.03000000 sec
TD0	1
SFO1	100.6228298 MHz
NUC1	13C
P0	3.33 usec
P1	10.00 usec
PLW1	53.28699875 W
SFO2	400.1316005 MHz
NUC2	1H
CPDPRG[2	waltz65
PCPD2	90.00 usec
PLW2	10.22500038 W
PLW12	0.36482000 W
PLW13	0.18350001 W

F2 - Processing parameters

SI	32768
SF	100.6127685 MHz
WDW	EM
SSB	0
LB	1.00 Hz
GB	0
PC	1.40

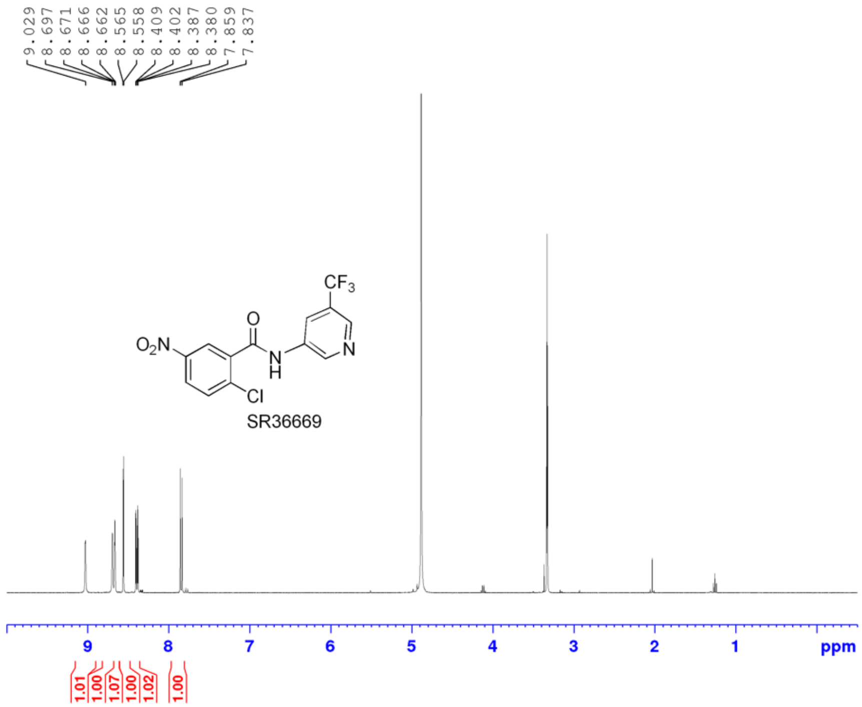


```
Current Data Parameters
NAME          zd-05-118-4
EXPNO                  10
PROCNO                  1

F2 - Acquisition Parameters
Date_            20220106
Time                15.07 h
INSTRUM  CAB AV4 400 MHZ BASIC
PROBHD   Z863001_0028 (
PULPROG              zg30
TD                  65536
SOLVENT              MeOD
NS                     16
DS                      2
SWH              8196.722 Hz
FIDRES           0.250144 Hz
AQ              3.9976959 sec
RG                    101
DW                 61.000 usec
DE                  12.35 usec
TE                  295.3 K
D1             2.00000000 sec
TD0                     1
SFO1          400.1324708 MHz
NUC1                   1H
P0                   5.67 usec
P1                  17.00 usec
PLW1          10.22500038 W

F2 - Processing parameters
SI                  65536
SF            400.1300000 MHz
WDW                    EM
SSB      0
LB                   0.30 Hz
GB       0
PC                   1.00
```

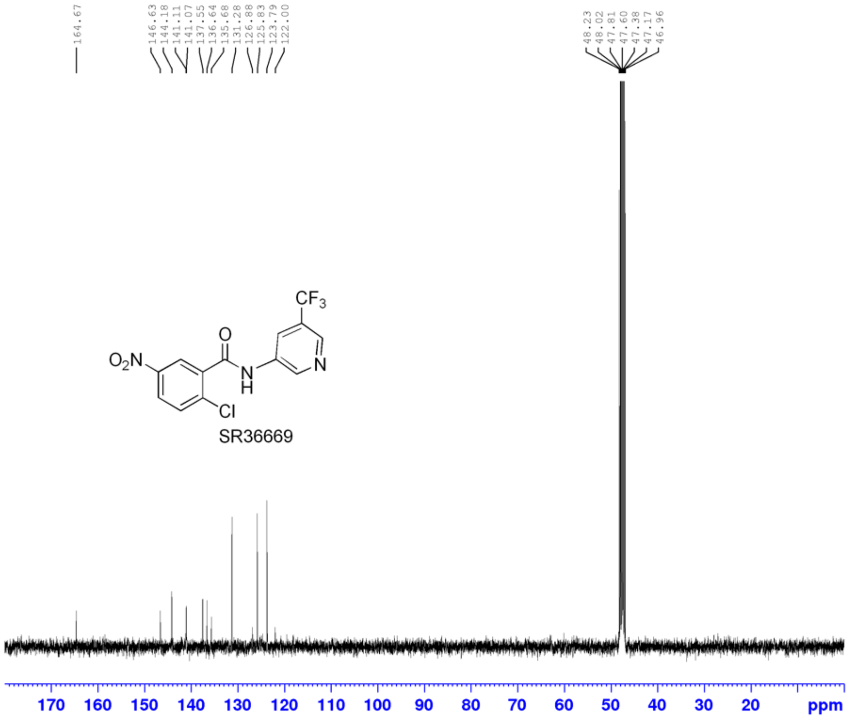


```
Current Data Parameters
NAME          zd-05-118-4
EXPNO                  20
PROCNO                  1

F2 - Acquisition Parameters
Date_            20220106
Time                14.06 h
INSTRUM  CAB AV4 400 MHZ BASIC
PROBHD   Z863001_0028 (
PULPROG            zgpg30
TD                  65536
SOLVENT              MeOD
NS                    400
DS                      4
SWH             23809.523 Hz
FIDRES           0.726609 Hz
AQ              1.3762560 sec
RG                    101
DW                 21.000 usec
DE                   6.50 usec
TE                  296.5 K
D1             2.00000000 sec
D11            0.03000000 sec
TD0                     1
SFO1          100.6228298 MHz
NUC1                  13C
P0                   3.33 usec
P1                  10.00 usec
PLW1          53.28699875 W
SFO2          400.1316005 MHz
NUC2                   1H
CPDPRG[2          waltz65
PCPD2               90.00 usec
PLW2          10.22500038 W
PLW12          0.36482000 W
PLW13          0.18350001 W

F2 - Processing parameters
SI                  32768
SF            100.6127685 MHz
WDW                    EM
SSB      0
LB                   1.00 Hz
GB       0
PC                   1.40
```

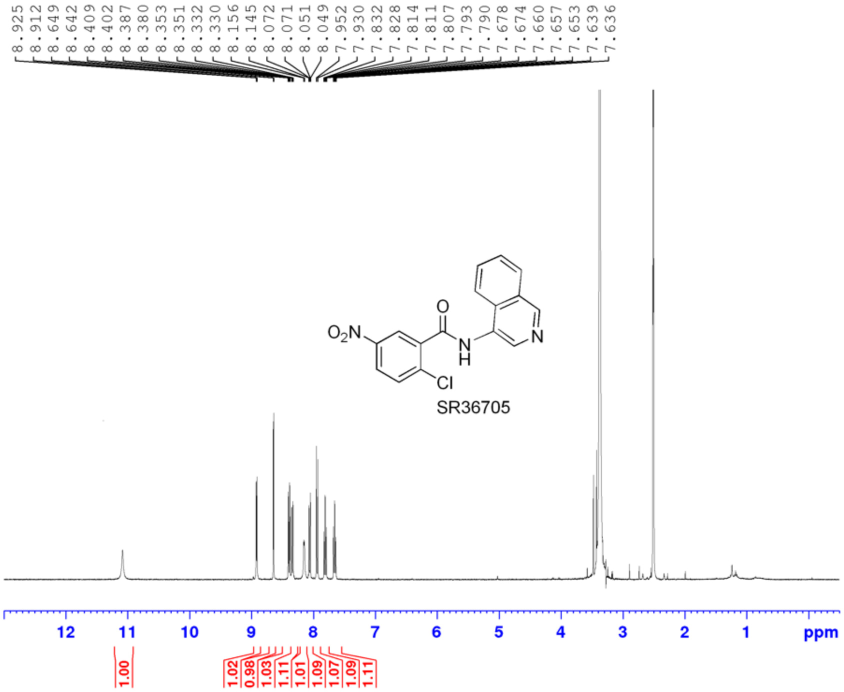


```
Current Data Parameters
NAME          zd-05-131-3
EXPNO                  10
PROCNO                  1

F2 - Acquisition Parameters
Date_            20220113
Time                 0.05 h
INSTRUM   CAB AV4 400 MHZ BASIC
PROBHD    Z863001_0028 (
PULPROG              zg30
TD                  65536
SOLVENT              DMSO
NS                     16
DS                      2
SWH              8196.722 Hz
FIDRES           0.250144 Hz
AQ              3.9976959 sec
RG                    101
DW                 61.000 usec
DE                  12.35 usec
TE                  295.7 K
D1             2.00000000 sec
TD0                     1
SFO1          400.1324708 MHz
NUC1                   1H
P0                   5.67 usec
P1                  17.00 usec
PLW1          10.22500038 W

F2 - Processing parameters
SI                  65536
SF            400.1300000 MHz
WDW                    EM
SSB      0
LB                   0.30 Hz
GB       0
PC                   1.00
```

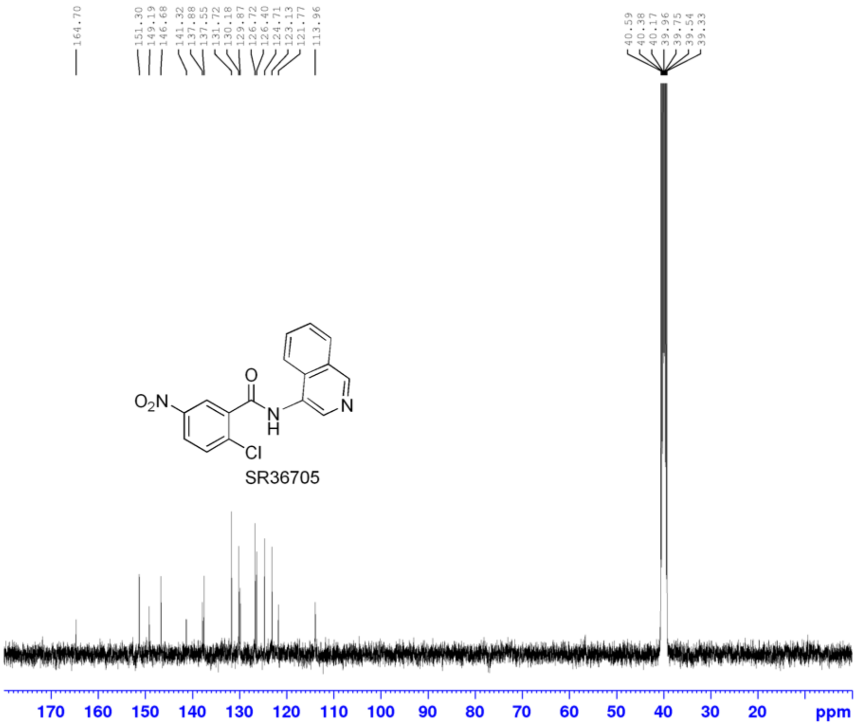

Current Data Parameters

NAME	zd-05-131-3	
EXPNO	11	
PROCNO	1	

F2 - Acquisition Parameters

Date_	20220113	
Time	3.55	h
INSTRUM	CAB AV4 400 MHZ BASIC	
PROBHD	Z863001_0028 (	
PULPROG	zgpg30	
TD	65536	
SOLVENT	DMSO	
NS	4000	
DS	4	
SWH	23809.523	Hz
FIDRES	0.726609	Hz
AQ	1.3762560	sec
RG	101	
DW	21.000	usec
DE	6.50	usec
TE	296.8	K
D1	2.00000000	sec
D11	0.03000000	sec
TD0	1	
SFO1	100.6228298	MHz
NUC1	13C	
P0	3.33	usec
P1	10.00	usec
PLW1	53.28699875	W
SFO2	400.1316005	MHz
NUC2	1H	
CPDPRG[2	waltz65	
PCPD2	90.00	usec
PLW2	10.22500038	W
PLW12	0.36482000	W
PLW13	0.18350001	W

F2 - Processing parameters

SI	32768	
SF	100.6127685	MHz
WDW	EM	
SSB	0	
LB	1.00	Hz
GB	0	
PC	1.40	

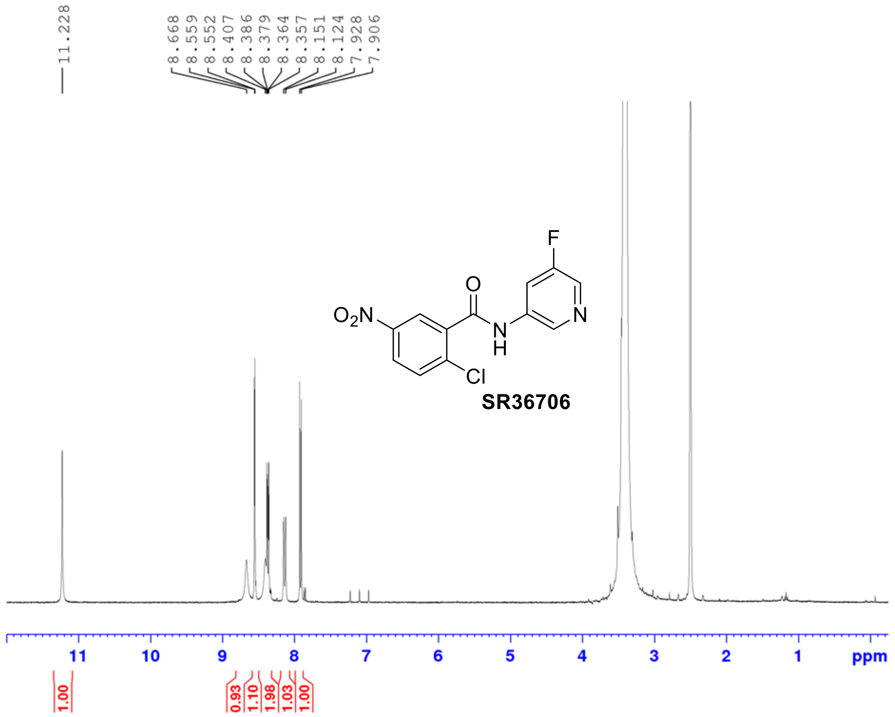


```
Current Data Parameters
NAME          zd-SR36706
EXPNO                 10
PROCNO                 1

F2 - Acquisition Parameters
Date_           20221026
Time              17.50 h
INSTRUM  CAB AV4 400 MHZ BASIC
PROBHD   Z863001_0028 (
PULPROG             zg30
TD                 65536
SOLVENT             DMSO
NS                    16
DS                     2
SWH            8196.722 Hz
FIDRES         0.250144 Hz
AQ            3.9976959 sec
RG                   101
DW                61.000 usec
DE                 12.35 usec
TE                 295.5 K
D1            2.00000000 sec
TD0                    1
SFO1         400.1324708 MHz
NUC1                  1H
P0                  5.67 usec
P1                 17.00 usec
PLW1         10.22500038 W

F2 - Processing parameters
SI                 65536
SF           400.1300028 MHz
WDW                   EM
SSB      0
LB                  0.30 Hz
GB       0
PC                  1.00
```

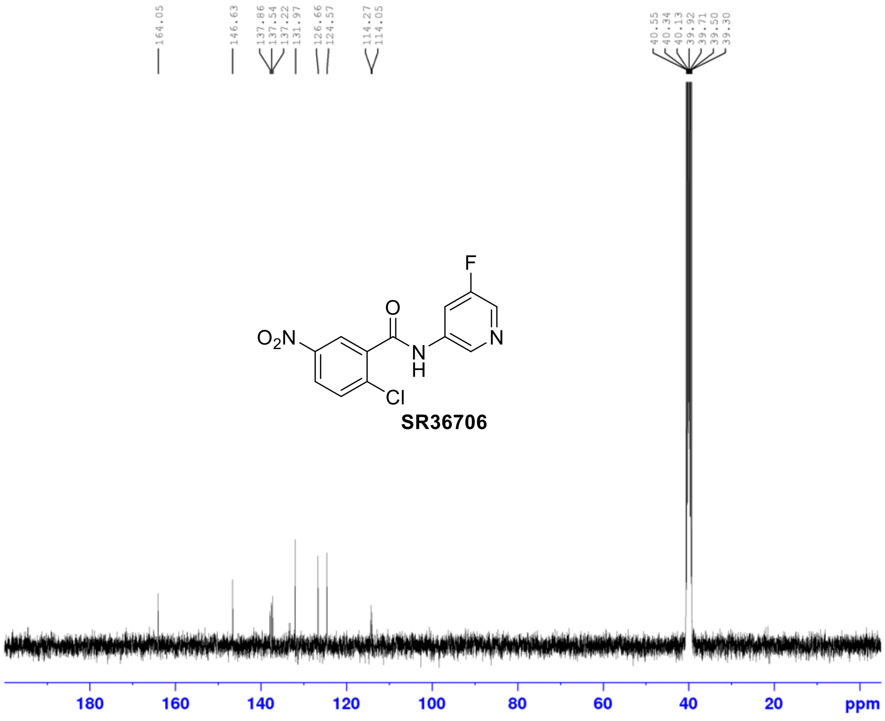


```
Current Data Parameters
NAME          zd-SR36706
EXPNO                 11
PROCNO                 1

F2 - Acquisition Parameters
Date_           20221026
Time              18.49 h
INSTRUM  CAB AV4 400 MHZ BASIC
PROBHD   Z863001_0028 (
PULPROG           zgpg30
TD                 65536
SOLVENT             DMSO
NS                  1024
DS                     4
SWH           23809.523 Hz
FIDRES         0.726609 Hz
AQ            1.3762560 sec
RG                   101
DW                21.000 usec
DE                  6.50 usec
TE                 296.6 K
D1            2.00000000 sec
D11           0.03000000 sec
TD0                    1
SFO1         100.6228298 MHz
NUC1                 13C
P0                  3.33 usec
P1                 10.00 usec
PLW1         53.28699875 W
SFO2         400.1316005 MHz
NUC2                  1H
CPDPRG[2         waltz65
PCPD2              90.00 usec
PLW2         10.22500038 W
PLW12         0.36482000 W
PLW13         0.18350001 W

F2 - Processing parameters
SI                 32768
SF           100.6127685 MHz
WDW                   EM
SSB      0
LB                  1.00 Hz
GB       0
PC                  1.40
```

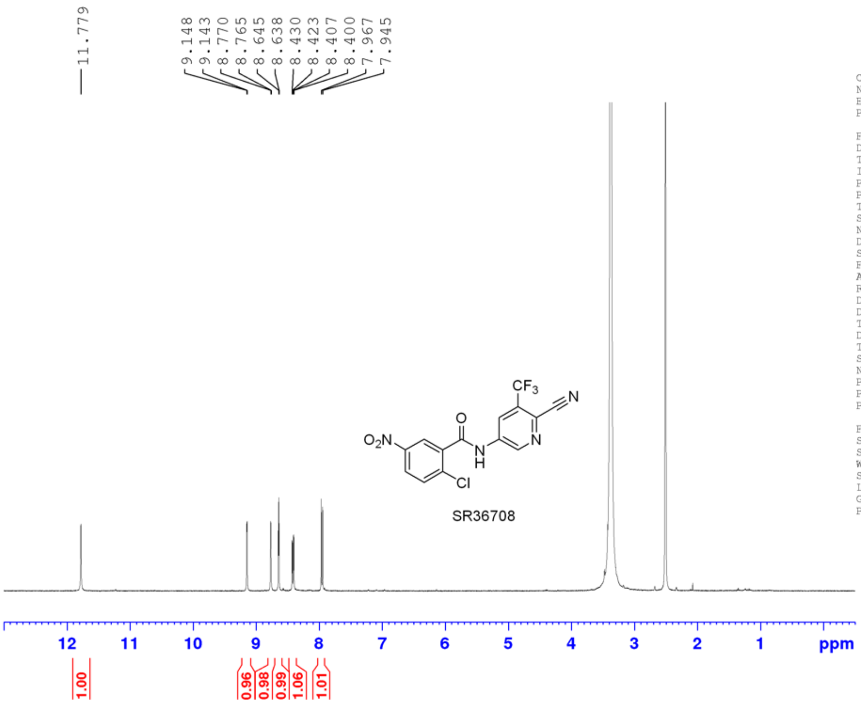


```
Current Data Parameters
NAME          zd-05-132-3
EXPNO                  10
PROCNO                  1

F2 - Acquisition Parameters
Date_            20220118
Time                10.56 h
INSTRUM  CAB AV4 400 MHZ BASIC
PROBHD   Z863001_0028 (
PULPROG              zg30
TD                  65536
SOLVENT              DMSO
NS                     16
DS                      2
SWH              8196.722 Hz
FIDRES           0.250144 Hz
AQ              3.9976959 sec
RG                    101
DW                 61.000 usec
DE                  12.35 usec
TE                  295.2 K
D1             2.00000000 sec
TD0                     1
SFO1          400.1324708 MHz
NUC1                   1H
P0                   5.67 usec
P1                  17.00 usec
PLW1          10.22500038 W

F2 - Processing parameters
SI                  65536
SF            400.1300000 MHz
WDW                    EM
SSB      0
LB                   0.30 Hz
GB       0
PC                   1.00
```

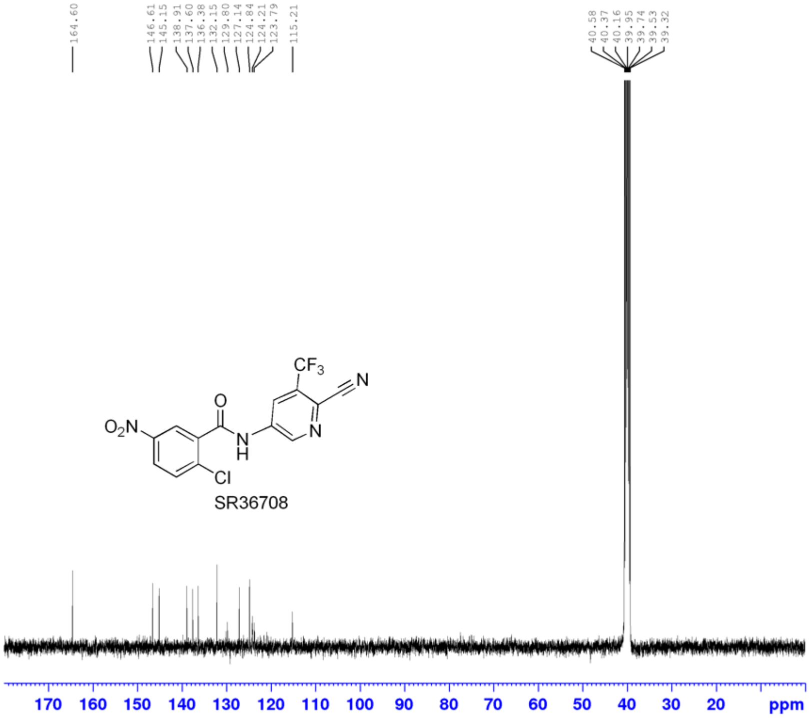


```
Current Data Parameters
NAME        zd-05-132-3
EXPNO                20
PROCNO                1

F2 - Acquisition Parameters
Date_          20220119
Time               0.43 h
INSTRUM   CAB AV4 400 MHZ BASIC
PROBHD    Z863001_0028 (
PULPROG          zgpg30
TD                65536
SOLVENT            DMSO
NS                 4500
DS                    4
SWH           23809.523 Hz
FIDRES         0.726609 Hz
AQ            1.3762560 sec
RG                  101
DW               21.000 usec
DE                 6.50 usec
TE                295.8 K
D1           2.00000000 sec
D11          0.03000000 sec
TD0                   1
SFO1        100.6228298 MHz
NUC1                13C
P0                 3.33 usec
P1                10.00 usec
PLW1        53.28699875 W
SFO2        400.1316005 MHz
NUC2                 1H
CPDPRG[2        waltz65
PCPD2             90.00 usec
PLW2        10.22500038 W
PLW12        0.36482000 W
PLW13        0.18350001 W

F2 - Processing parameters
SI                32768
SF          100.6127685 MHz
WDW                  EM
SSB      0
LB                 1.00 Hz
GB       0
PC                 1.40
```

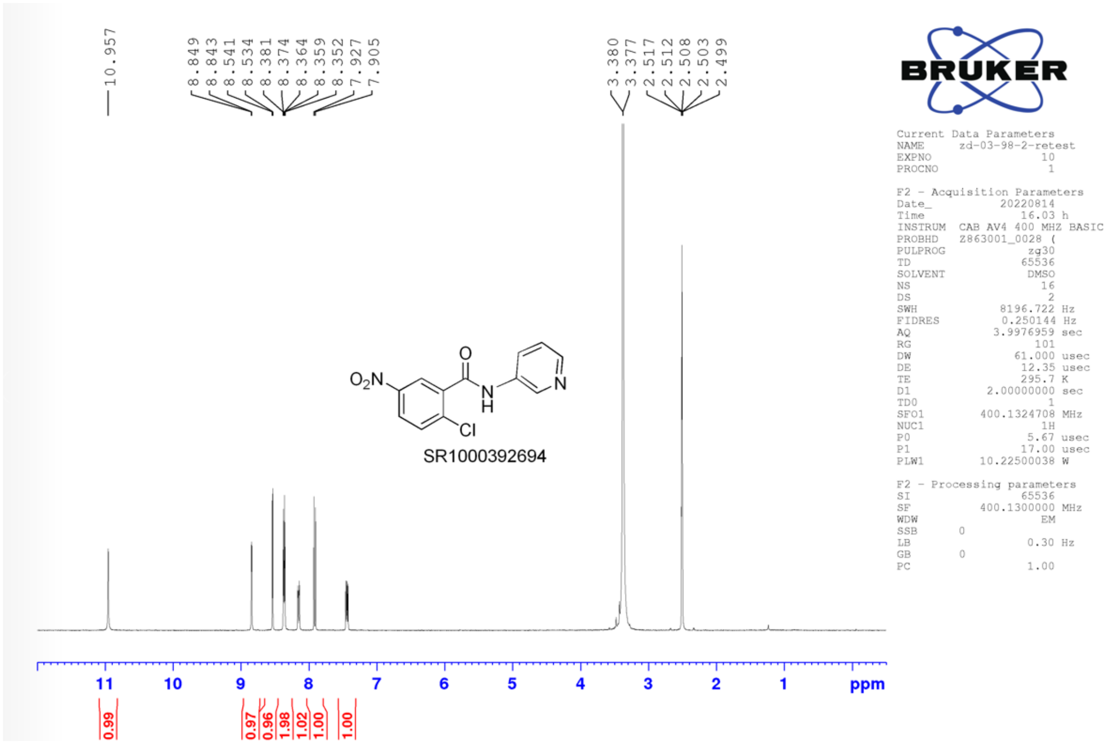
10.957
8.849
8.843
8.541
8.534
8.381
8.374
8.364
8.359
8.352
7.927
7.905
3.380
3.377
2.517
2.512
2.508
2.503
2.499
O_2N
O
N
H
N
Cl
SR1000392694
11 10 9 8 7 6 5 4 3 2 1 ppm
0.99
0.97
0.96
1.98
1.02
1.00
1.00
BRUKER
Current Data Parameters
NAME zd-03-98-2-retest
EXPNO 10
PROCNO 1
F2 - Acquisition Parameters
Date_ 20220814
Time 16.03 h
INSTRUM CAB AV4 400 MHZ BASIC
PROBHD Z863001_0028 (
PULPROG zg30
TD 65536
SOLVENT DMSO
NS 16
DS 2
SWH 8196.722 Hz
FIDRES 0.250144 Hz
AQ 3.9976959 sec
RG 101
DW 61.000 usec
DE 12.35 usec
TE 295.7 K
D1 2.00000000 sec
TD0 1
SFO1 400.1324708 MHz
NUC1 1H
P0 5.67 usec
P1 17.00 usec
PLW1 10.22500038 W
F2 - Processing parameters
SI 65536
SF 400.1300000 MHz
WDW EM
SSB 0
LB 0.30 Hz
GB 0
PC 1.00

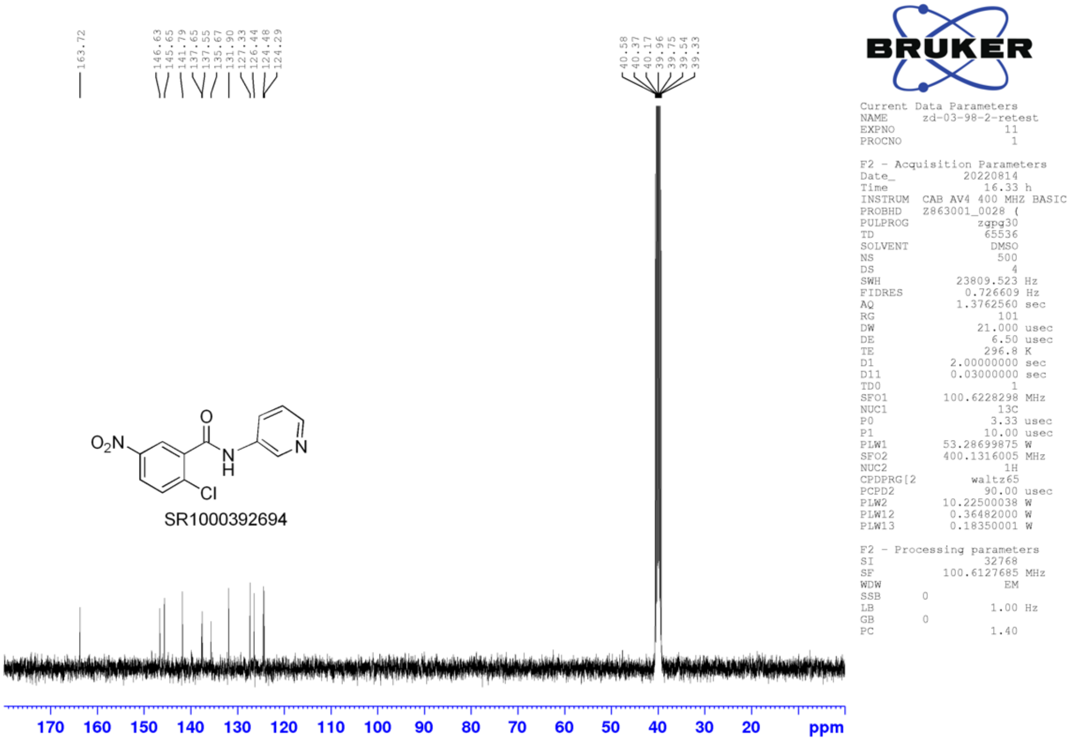
163.72
146.63
145.65
141.79
137.65
137.55
135.67
131.90
127.33
126.44
124.48
124.29
40.58
40.37
40.17
39.96
39.75
39.54
39.33
O_2N
O
N
H
N
Cl
SR1000392694
170 160 150 140 130 120 110 100 90 80 70 60 50 40 30 20 ppm
BRUKER
Current Data Parameters
NAME zd-03-98-2-retest
EXPNO 11
PROCNO 1
F2 - Acquisition Parameters
Date_ 20220814
Time 16.33 h
INSTRUM CAB AV4 400 MHZ BASIC
PROBHD Z863001_0028 (
PULPROG zgpg30
TD 65536
SOLVENT DMSO
NS 500
DS 4
SWH 23809.523 Hz
FIDRES 0.726609 Hz
AQ 1.3762560 sec
RG 101
DW 21.000 usec
DE 6.50 usec
TE 296.8 K
D1 2.00000000 sec
D11 0.03000000 sec
TD0 1
SFO1 100.6228298 MHz
NUC1 13C
P0 3.33 usec
P1 10.00 usec
PLW1 53.28699875 W
SFO2 400.1316005 MHz
NUC2 1H
CPDPRG[2 waltz65
PCPD2 90.00 usec
PLW2 10.22500038 W
PLW12 0.36482000 W
PLW13 0.18350001 W
F2 - Processing parameters
SI 32768
SF 100.6127685 MHz
WDW EM
SSB 0
LB 1.00 Hz
GB 0
PC 1.40

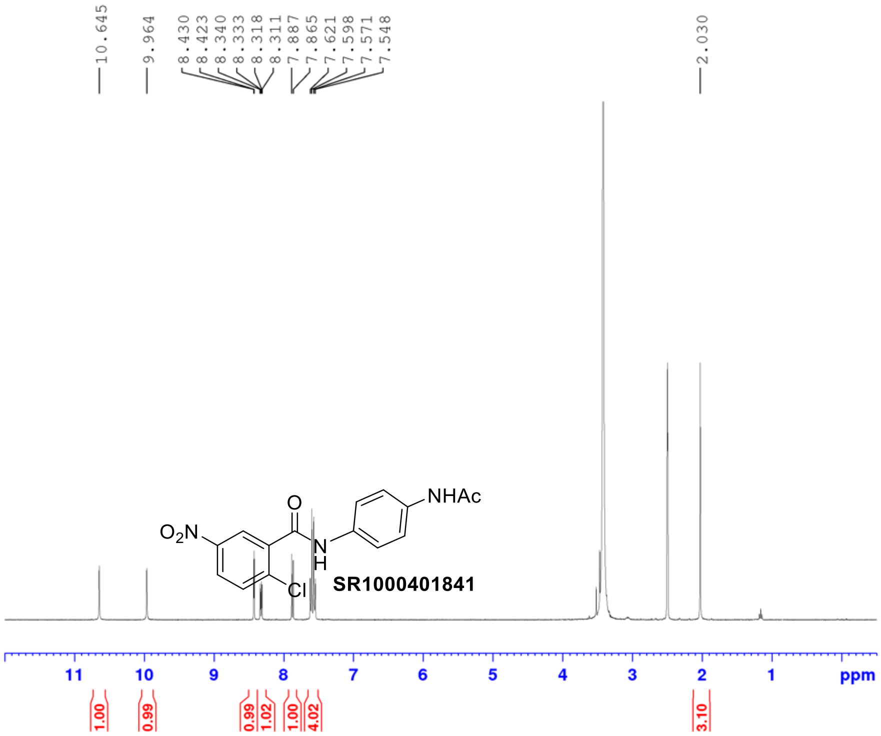


```
Current Data Parameters
NAME      zd-SR1000401841
EXPNO                  10
PROCNO                  1

F2 - Acquisition Parameters
Date_            20221026
Time                16.01 h
INSTRUM  CAB AV4 400 MHZ BASIC
PROBHD   Z863001_0028 (
PULPROG              zg30
TD                  65536
SOLVENT              DMSO
NS                     16
DS                      2
SWH              8196.722 Hz
FIDRES           0.250144 Hz
AQ              3.9976959 sec
RG                    101
DW                 61.000 usec
DE                  12.35 usec
TE                  295.2 K
D1             2.00000000 sec
TD0                     1
SFO1          400.1324708 MHz
NUC1                   1H
P0                   5.67 usec
P1                  17.00 usec
PLW1          10.22500038 W

F2 - Processing parameters
SI                  65536
SF            400.1300029 MHz
WDW                    EM
SSB      0
LB                   0.30 Hz
GB       0
PC                   1.00
```

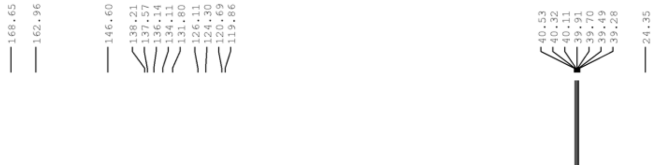


```
Current Data Parameters
NAME      zd-SR1000401841
EXPNO                  11
PROCNO                  1

F2 - Acquisition Parameters
Date_            20221026
Time                16.31 h
INSTRUM  CAB AV4 400 MHZ BASIC
```

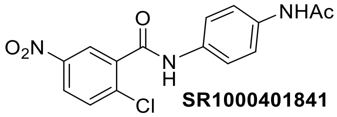

SR1000401841

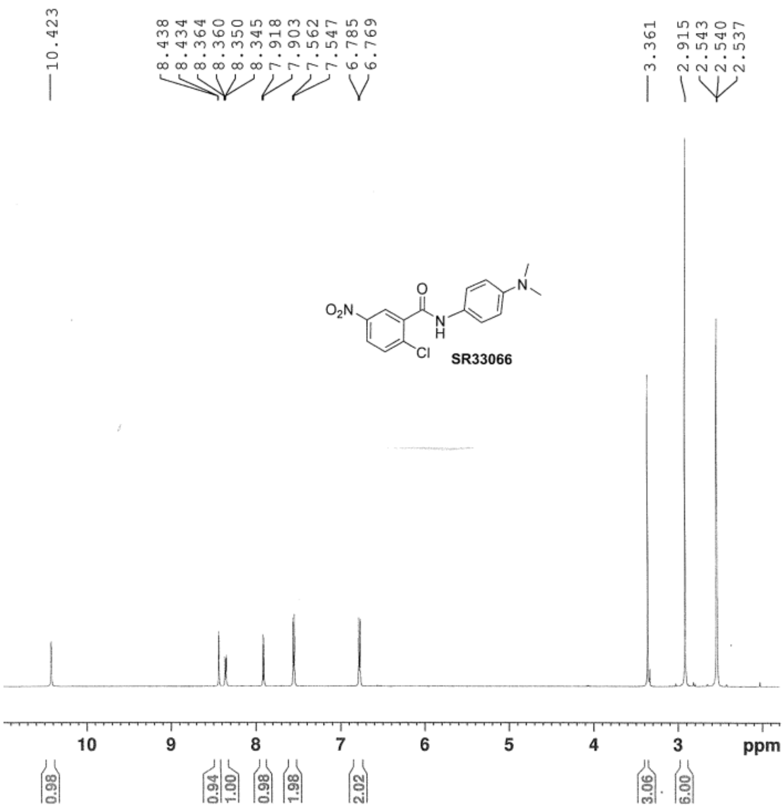

Current Data Parameters
NAME SR33066
EXPNO 10
PROCNO 1

F2 - Acquisition Parameters
Date_ 20241125
Time 16.27 h
INSTRUM CAB AV4 600 MHZ BASIC
PROBHD Z161159_0005 (
PULPROG zg30
TD 65536
SOLVENT DMSO
NS 16
DS 2
SWH 11904.762 Hz
FIDRES 0.363304 Hz
AQ 2.7525120 sec
RG 25.4
DW 42.000 usec
DE 14.42 usec
TE 298.1 K
D1 2.00000000 sec
TD0 1
SFO1 600.1837061 MHz
NUC1 1H
P0 2.60 usec
P1 7.80 usec
PLW1 5.68470001 W

F2 - Processing parameters
SI 65536
SF 600.1799813 MHz
WDW EM
SSB 0
LB 0.30 Hz
GB 0
PC 1.00

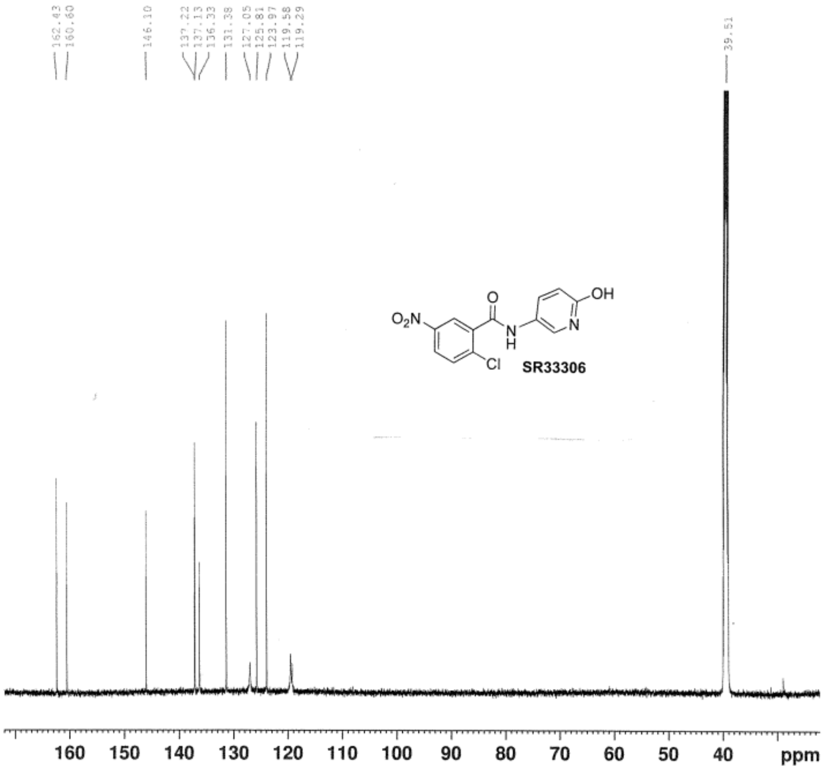

Current Data Parameters
NAME SR33306
EXPNO 11
PROCNO 1

F2 - Acquisition Parameters
Date_ 20241127
Time 7.29
INSTRUM CAB AV4 600 MHZ BASIC
PROBHD Z161159_0005 (
PULPROG zgpg30
TD 65536
SOLVENT DMSO
NS 2048
DS 4
SWH 35714.285 Hz
FIDRES 0.544957 Hz
AQ 0.9175040 sec
RG 32
DW 14.000 usec
DE 18.00 usec
TE 298.1 K
D1 2.00000000 sec
d11 0.03000000 sec
DELTA 1.89999998 sec
TD0 1
SFO1 150.9304726 MHz
NUC1 13C
P1 11.90 usec
PLW1 87.09600067 W
SFO2 600.1824007 MHz
NUC2 1H
CPDPRG[2 waltz65
PCPD2 70.00 usec
PLW2 5.68470001 W
PLW12 0.07058300 W
PLW13 0.03550300 W

F2 - Processing parameters
SI 32768
SF 150.9154537 MHz
WDW EM
SSB 0
LB 1.00 Hz
GB 0
PC 1.40

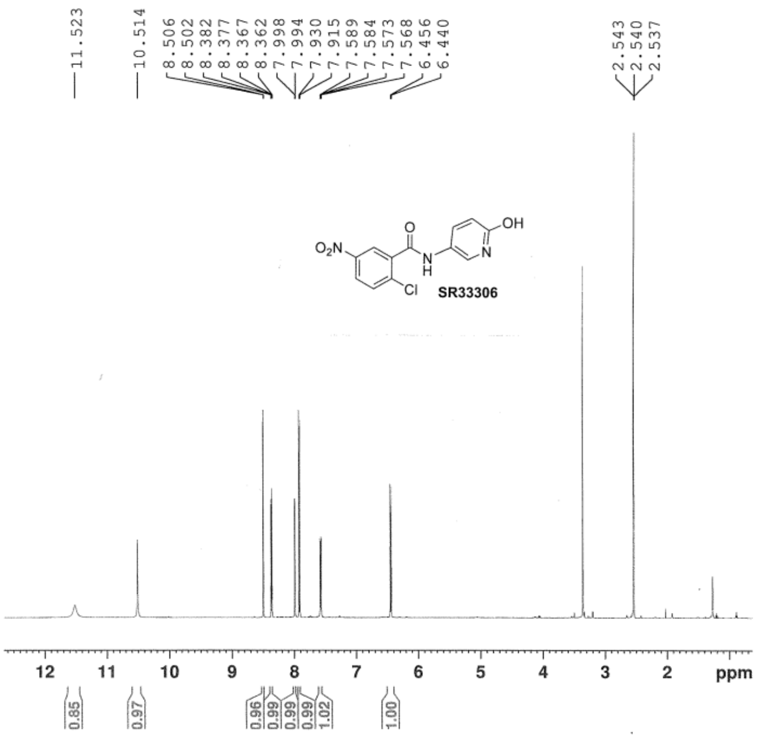

BRUKER

```
Current Data Parameters
NAME              SR33306
EXPNO                  10
PROCNO                  1

F2 - Acquisition Parameters
Date_           20241127
Time              5.45 h
INSTRUM   CAB AV4 600 MHZ BASIC
PROBHD    Z161159_0005 (
PULPROG              zg30
TD                  65536
SOLVENT              DMSO
NS                     16
DS                      2
SWH             11904.762 Hz
FIDRES           0.363304 Hz
AQ              2.7525120 sec
RG                   22.6
DW                 42.000 usec
DE                  14.42 usec
TE                  298.1 K
D1             2.00000000 sec
TD0                     1
SFO1          600.1837061 MHz
NUC1                   1H
P0                   2.60 usec
P1                   7.80 usec
PLW1           5.68470001 W

F2 - Processing parameters
SI                  65536
SF            600.1799815 MHz
WDW                    EM
SSB      0
LB                   0.30 Hz
GB       0
PC                   1.00
```

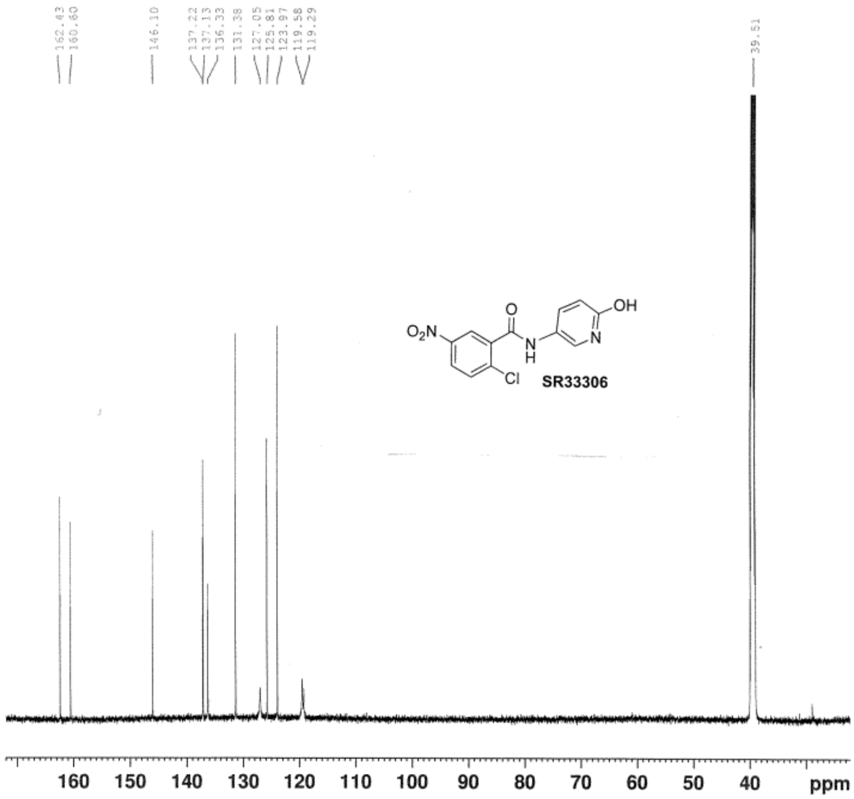

BRUKER

```
Current Data Parameters
NAME              SR33306
EXPNO                  11
PROCNO                  1

F2 - Acquisition Parameters
Date_           20241127
Time                 7.29
INSTRUM   CAB AV4 600 MHZ BASIC
PROBHD    Z161159_0005 (
PULPROG            zgpg30
TD                  65536
SOLVENT              DMSO
NS                   2048
DS                      4
SWH             35714.285 Hz
FIDRES           0.544957 Hz
AQ              0.9175040 sec
RG                     32
DW                 14.000 usec
DE                  18.00 usec
TE                  298.1 K
D1             2.00000000 sec
d11            0.03000000 sec
DELTA          1.89999998 sec
TD0                     1
SFO1          150.9304726 MHz
NUC1                  13C
P1                  11.90 usec
PLW1          87.09600067 W
SFO2          600.1824007 MHz
NUC2                   1H
CPDPRG[2           waltz65
PCPD2               70.00 usec
PLW2           5.68470001 W
PLW12          0.07058300 W
PLW13          0.03550300 W

F2 - Processing parameters
SI                  32768
SF            150.9154537 MHz
WDW                    EM
SSB      0
LB                   1.00 Hz
GB       0
PC                   1.40
```
