# Supplementary material for: Ligand efficacy shifts a nuclear receptor conformational ensemble between transcriptionally active and repressive states
Source: Nat Commun. 2025 Feb 28;16:2065. doi: 10.1038/s41467-025-57325-4 (PMC11871303; doi:10.1038/s41467-025-57325-4)
Supplement: Supplementary file 3 [file 41467_2025_57325_MOESM4_ESM.zip › SOURCE DATA/Figure 4/correlation analysis for Figure 6b.pdf]

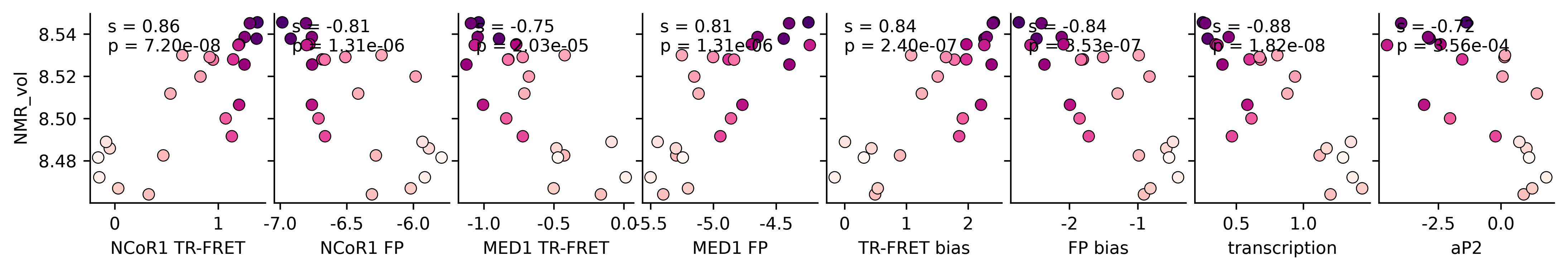
NMR_vol
s = 0.86
p = 7.20e-08
NCoR1 TR-FRET
s = -0.81
p = 1.31e-06
NCoR1 FP
s = -0.75
p = 2.03e-05
MED1 TR-FRET
s = 0.81
p = 1.31e-06
MED1 FP
s = 0.84
p = 2.40e-07
TR-FRET bias
s = -0.84
p = 3.53e-07
FP bias
s = -0.88
p = 1.82e-08
transcription
s = -0.72
p = 3.56e-04
aP2
