# Supplementary material for: Ligand efficacy shifts a nuclear receptor conformational ensemble between transcriptionally active and repressive states
Source: Nat Commun. 2025 Feb 28;16:2065. doi: 10.1038/s41467-025-57325-4 (PMC11871303; doi:10.1038/s41467-025-57325-4)
Supplement: Supplementary file 3 [file 41467_2025_57325_MOESM4_ESM.zip › SOURCE DATA/Figure 4/correlation analysis for Figure 4.pdf]

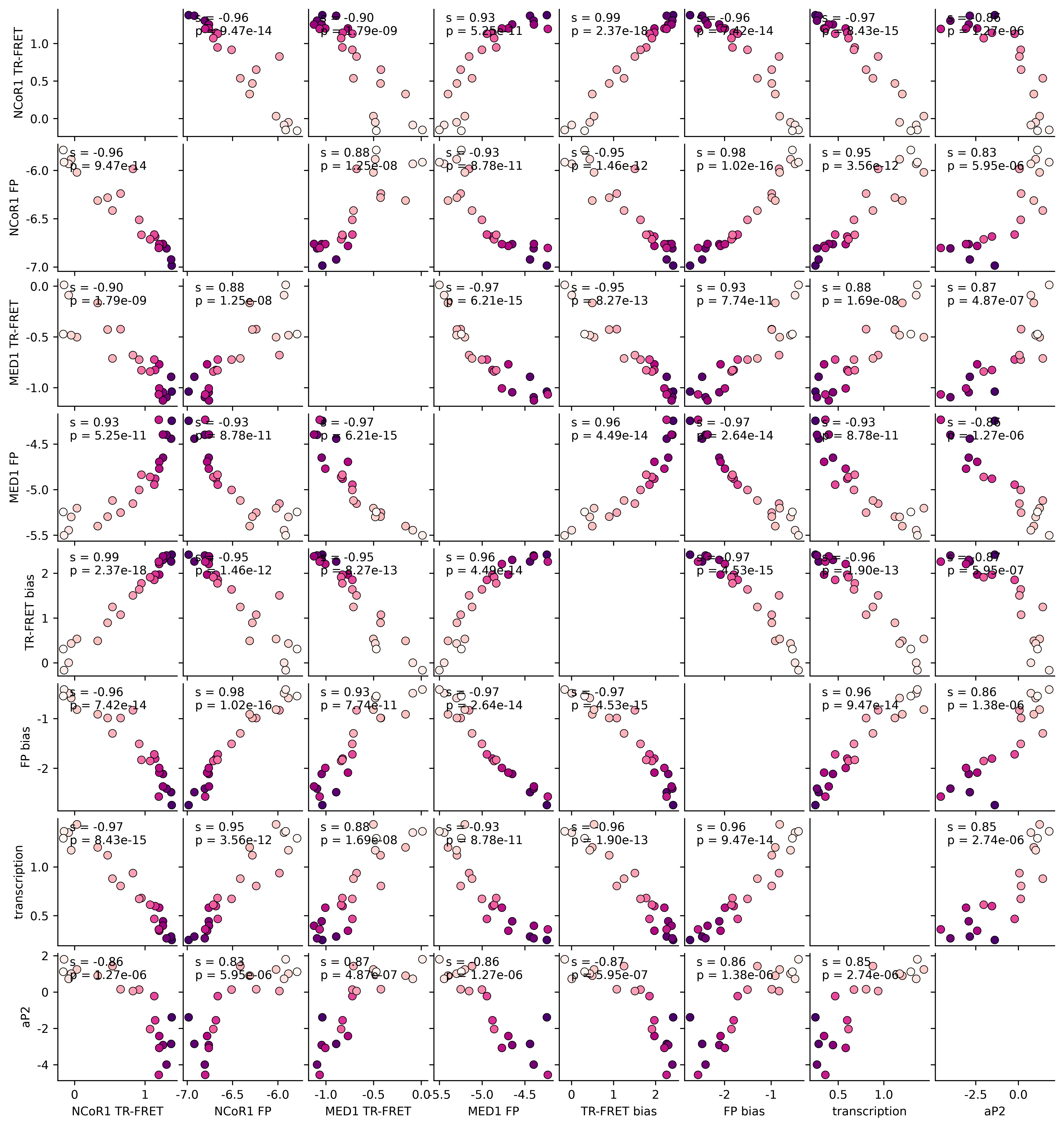
NCoR1 TR-FRET
NCoR1 FP
MED1 TR-FRET
MED1 FP
TR-FRET bias
FP bias
transcription
aP2
s = -0.96 p = 9.47e-14
s = -0.90 p = 1.79e-09
s = 0.93 p = 5.25e-11
s = 0.99 p = 2.37e-18
s = -0.96 p = 7.42e-14
s = -0.97 p = 8.43e-15
s = -0.86 p = 1.27e-06
s = 0.88 p = 1.25e-08
s = -0.93 p = 8.78e-11
s = -0.95 p = 1.46e-12
s = 0.98 p = 1.02e-16
s = 0.95 p = 3.56e-12
s = 0.83 p = 5.95e-06
s = -0.97 p = 6.21e-15
s = -0.95 p = 8.27e-13
s = 0.93 p = 7.74e-11
s = 0.88 p = 1.69e-08
s = 0.87 p = 4.87e-07
s = 0.96 p = 4.49e-14
s = -0.97 p = 2.64e-14
s = -0.97 p = 4.53e-15
s = -0.96 p = 1.90e-13
s = -0.87 p = 5.95e-07
s = 0.96 p = 9.47e-14
s = 0.86 p = 1.38e-06
s = 0.85 p = 2.74e-06
