# Supplementary material for: Ligand efficacy shifts a nuclear receptor conformational ensemble between transcriptionally active and repressive states
Source: Nat Commun. 2025 Feb 28;16:2065. doi: 10.1038/s41467-025-57325-4 (PMC11871303; doi:10.1038/s41467-025-57325-4)
Supplement: Supplementary file 3 [file 41467_2025_57325_MOESM4_ESM.zip › SOURCE DATA/Figure 4/correlation analysis for Supplementary Figure 2.pdf]

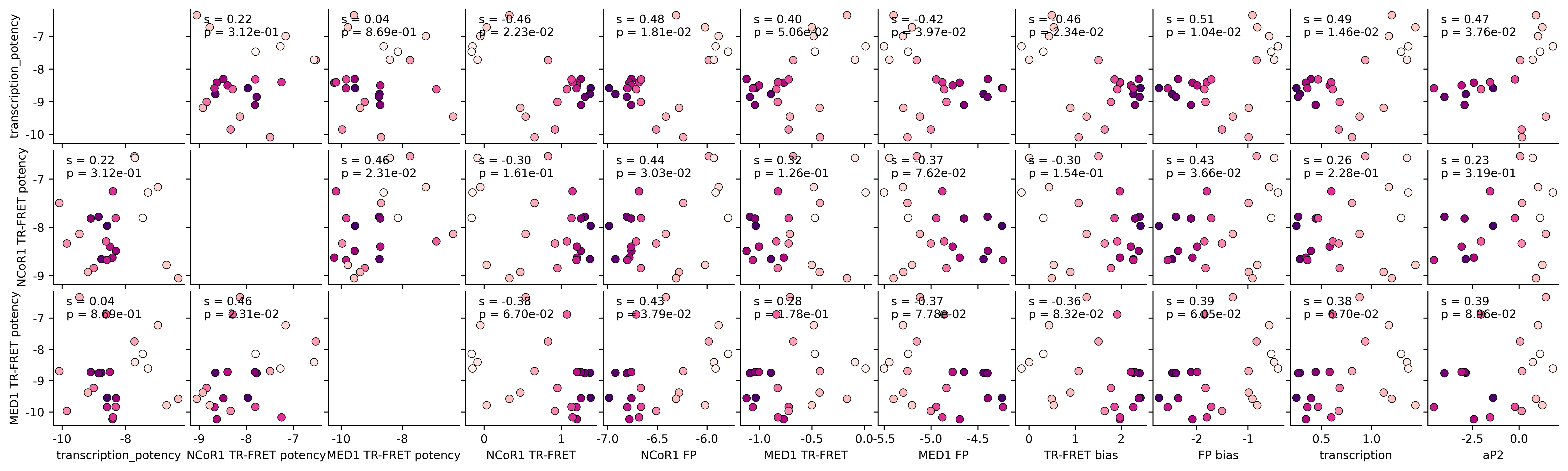
transcription_potency
NCoR1 TR-FRET potency
MED1 TR-FRET potency
s = 0.22
p = 3.12e-01
s = 0.04
p = 8.69e-01
s = -0.46
p = 2.23e-02
s = 0.48
p = 1.81e-02
s = 0.40
p = 5.06e-02
s = -0.42
p = 3.97e-02
s = -0.46
p = 2.34e-02
s = 0.51
p = 1.04e-02
s = 0.49
p = 1.46e-02
s = 0.47
p = 3.76e-02
s = 0.22
p = 3.12e-01
s = 0.46
p = 2.31e-02
s = -0.30
p = 1.61e-01
s = 0.44
p = 3.03e-02
s = 0.32
p = 1.26e-01
s = -0.37
p = 7.62e-02
s = -0.30
p = 1.54e-01
s = 0.43
p = 3.66e-02
s = 0.26
p = 2.28e-01
s = 0.23
p = 3.19e-01
s = 0.04
p = 8.69e-01
s = 0.46
p = 2.31e-02
s = -0.38
p = 6.70e-02
s = 0.43
p = 3.79e-02
s = 0.28
p = 1.78e-01
s = -0.37
p = 7.78e-02
s = -0.36
p = 8.32e-02
s = 0.39
p = 6.05e-02
s = 0.38
p = 6.70e-02
s = 0.39
p = 8.96e-02
transcription_potency
NCoR1 TR-FRET potency
MED1 TR-FRET potency
NCoR1 TR-FRET
NCoR1 FP
MED1 TR-FRET
MED1 FP
TR-FRET bias
FP bias
transcription
aP2
